# Hierarchical Chiral Self-Assembly of Nanocylinders Composed of Sequence-Defined Mesogenic Dimers

**DOI:** 10.1021/jacs.6c05456

**Published:** 2026-06-17

**Authors:** Emily C. Ostermann, Chun Lam Clement Chan, Justin Bendesky, Jacob S. Votava, Eva S. M. Reed, Shawn M. Maguire, Michael A. Webb, Stephanie S. Lee, Bart Kahr, Emily C. Davidson

**Affiliations:** † Department of Chemical and Biological Engineering, 6740 Princeton University, Princeton, New Jersey 08540, United States; ‡ Department of Chemistry and Molecular Design Institute, 5894 New York University, New York, New York 10003, United States

## Abstract

Chiral ensembles can arise through supramolecular curvature that resolves geometric frustrations in the packing of bent, achiral molecular or colloidal building blocks. Here, we leverage orthogonal protection–deprotection click chemistry to create sequence-defined mesogenic heterodimers exhibiting emergent chirality. We compare the hierarchical self-assembly of the synthesized asymmetric, achiral heterodimers, which differ only in the position of a methyl substituent. Both dimers form chiral spherulites composed of nanocylinders. However, the detailed arrangement of nanocylinders depends on the position of the methyl substituent and the crystallization conditions. Despite the chemical similarity, in one dimer, two crystalline forms are optically active. They form conglomerates of dextrorotatory and levorotatory spherulites. The other dimer forms more highly anisotropic spherulites that mask circular birefringence arising from the misorientation of nanocylinders, while mapping of nanocylinder directors reveals a sense at the spherulite surface. We propose that differences in nanocylinder arrangements may arise from changes in nanocylinder curvature and dimensions dictated by the methyl substituent position, inducing chirality. These results demonstrate multiscale hierarchical assembly relevant to dense systems of tubular structures and highlight the role of sequence and molecular design in directing the bottom-up hierarchical self-assembly and chirality of mesogenic systems.

## Graphical abstract

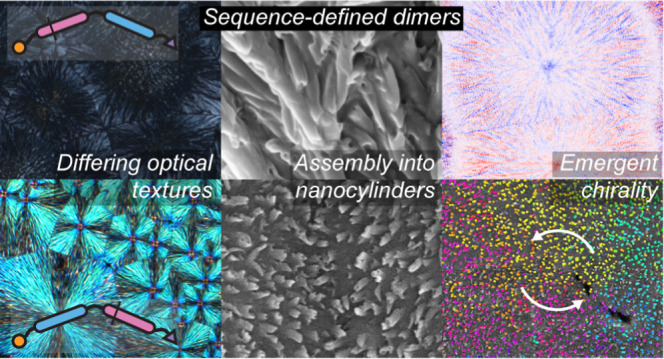

## Introduction

Chiral supramolecular assemblies are significant for biology^1–3^ and for synthetic applications, such as circularly polarized emission,^4,5^ chiral sensing,^6,7^ and catalysis.^8,9^ In most systems, handedness is introduced by using inherently chiral building blocks or chiral dopants^10,11^ or through self-assembly with a dissymmetric influence.^12–15^ Supramolecular chirality can also be induced spontaneously, driven by molecular conformation and intermolecular interactions^16–19^ or by particle shape and colloidal interactions.^20^ Understanding emergent chirality and its influence on the bulk is important for the design of new materials systems.

Liquid crystalline (LC) materials in which the building block has a bent shape can adopt a range of assemblies inaccessible to conventional rod and disc-like liquid crystals. While a bent molecular conformation is not the only factor driving supramolecular curvature, as evidenced by the twisting and bending mechanisms in molecular crystals,^21–24^ a bent molecular geometry plays a substantial role in the supramolecular order of LC phases. Bending can reduce rotational degrees of freedom resulting in packing frustrations and chiral, curved assemblies. These supramolecular assemblies, which include the “banana” *B* phases^25^ and modulated nematic phases,^16,26^ have been demonstrated across a large range of building block length scales, from small molecules,^19,27–29^ to oligomers^30,31^ and polymers,^32^ and extending to colloids.^20,33,34^ The “bend” within the building blocks can be well-defined by a discrete angle (e.g., kinked colloids^34^ or rigid bent-core mesogens), vary smoothly (e.g., curved colloidal rods),^33^ or result from an ensemble-averaged bent conformation, most studied in the case of mesogenic dimers.^35–37^

The self-assembly of these materials featuring a “bent” geometry often results in phases defined by supramolecular curvature, such as crystalline helical nanofilaments (HNF)^28^ and modulated nematic phases. These assemblies can adopt both chiral and achiral configurations, for example (chiral) twist-bend^38^ versus (achiral) splay-bend nematics.^39^ Whether the resulting configuration is chiral or achiral depends on how the frustration induced by the bent shape of the building block manifests in the supramolecular assembly.

One mechanism through which bent-shaped LC materials are known to exhibit chirality is a twist deformation. Notably, rigid bent-core mesogens^28,40^ and mesogenic dimers^41–43^ have formed HNFs, often classified as a crystalline manifestation of a *B*_4_ phase. In these systems, layer frustration arising from a bent intermesogen geometry has been postulated to lead to the adoption of symmetry-breaking saddle-splay curvature at a molecular level.^28^ However, a saddle-splay motif cannot fill space uniformly, necessitating that at a supramolecular level, the material must exhibit additional curvature, leading to a twist deformation and the formation of helical filaments. HNF formation can occur in systems of either chiral or achiral mesogens, with the former producing HNFs of a single twist sense and the latter producing a racemate of homochiral HNF domains.^40,44^

Such chiral LC assemblies encompass both highly ordered smectic or crystalline phases with limited molecular mobility, such as HNFs^45^ and discrete helical nanofilaments,^46^ and more fluid phases, including sponge morphologies such as the “plumber’s nightmare”.^47,48^ Despite the structural differences between these systems, they can all form a conglomerate of homochiral domains. In the absence of molecular chirality, this conglomerate is racemic, resulting in an optical texture in which the handedness of each domain can be qualitatively revealed by decrossing the polarizer/analyzer, or quantitatively analyzed via polarimetry techniques such as Mueller matrix imaging (MMI).^49^ In some cases, these conglomerates possess very low observed linear retardance (LR), such that they appear dark under crossed polarizers. This optical texture is commonly referred to as a “dark conglomerate” (DC).^47,50^

Besides saddle-splay and twist deformations, the frustration induced by a bent geometry can also be relieved through cylindrical curvature, including nanocylinder formation. In general, nanocylinders form when thin sheets experience asymmetric surface stresses. These stresses can arise from a number of factors, including mismatched unit-cell dimension^51^ and imbalanced surface tensions due to solvent–crystal interactions.^52^ In bent mesogenic systems, mesogen asymmetry and geometry are considered key parameters in driving the adoption of microstructures with cylindrical curvature over twisted structures with Gaussian (e.g., saddle-splay) curvature. For example, in a system of rigid bent-core mesogens, the placement of a stereogenic center with a methyl substituent on the longer aliphatic tail resulted in heliconical layered nanocylinders (HLNCs). In contrast, its placement on the shorter tail induced twist curvature and the formation of helical microfilaments.^53^ The methyl substituent position in these chiral bent-core mesogens has been postulated to modify the angle between the mesogenic core and the flexible tail chains, thereby determining the adopted curvature.^54,55^ It has also been shown in flexible azobenzene dimers that longer terminal chain lengths promote the formation of heliconical-layered microcylinders (HLμC) over HNFs.^43^ However, compared with HNF systems, the supramolecular chirality of the nanocylinder structures is not well established. While optical rotation has been observed in a system of coexisting HNFs and HLμCs,^43^ DC optical textures in a nanocylindrical system have only been observed in achiral mesogenic trimers, where the emergent chirality was attributed to a helical “wrapping” deformation of layers.^56^ Despite progress in understanding the local assembly mechanisms of bent mesogenic systems, these models alone cannot explain the observed bulk behavior of these hierarchically assembled materials. These bulk properties are not solely contingent on molecular assembly within a single helical nanofilament or nanocylinder and depend on higher-order arrangements.^40,55,57^

Beyond LC materials, self-assembly into filamentous and tubular structures is observed in a wide range of systems, with particular relevance to biological environments. Many functional biological structures consist of self-assembled fibers, such as those composed of collagen,^58^ actin,^59^ or fibrin,^60^ for which their growth processes and hierarchical arrangements are crucial for maintaining cell structural integrity and directing functions such as cell motility and coagulation. Similar structures are also observed in synthesized biological or biocompatible molecules,^61–66^ organic small molecules,^67,68^ polymers,^69^ replicators,^70^ and organogels.^71,72^ Often the chirality of subunits and the influence of their packing modes leads to self-assembled filaments or tubules with supramolecular chirality,^73^ and these structures can further exhibit chiral hierarchical order such as twisted bundle formation.^74,75^ The interplay of molecular structure and growth mechanisms plays a key role in the formation of supramolecular curvature and multiscale chiral self-assembly.

Here, we synthesize two sequence-defined mesogenic dimers that possess similar chemical structures and assemble locally into nanocylinders of similar dimensions with hierarchical arrangements (Figure 1A). However, these dimers exhibit dramatically different optical responses on crystallization. Although they differ in chemical structure only by the position of a methyl substituent, one dimer exhibits optical rotation with sense-specific spherulites, while the other displays a strong chaotic signal for circular birefringence due to the misoverlap of nanocylinders. While the chaotic circular birefringence of the latter dimer does not indicate sense-specific spherulites, mapping of nanocylinder orientations reveals a cyclic sense at the spherulite surface. To investigate how such a small molecular difference can induce this discrepancy, we characterized the hierarchical assembly of these dimers, from molecular arrangement to nanocylinder orientation, under controlled crystallization and geometric confinement conditions. We then correlated their optical properties, including optical rotation, with the higher-order arrangement of nanocylinders, characterized by twisted bundles, periodic splay, and varying degrees of orientational coherence. This work reveals that the manifestation of supramolecular chirality results not only from simple geometric considerations, but also a delicate interplay between changes in molecular structure, confinement, and the thermodynamics and kinetics of crystallization.

**Figure 1:**
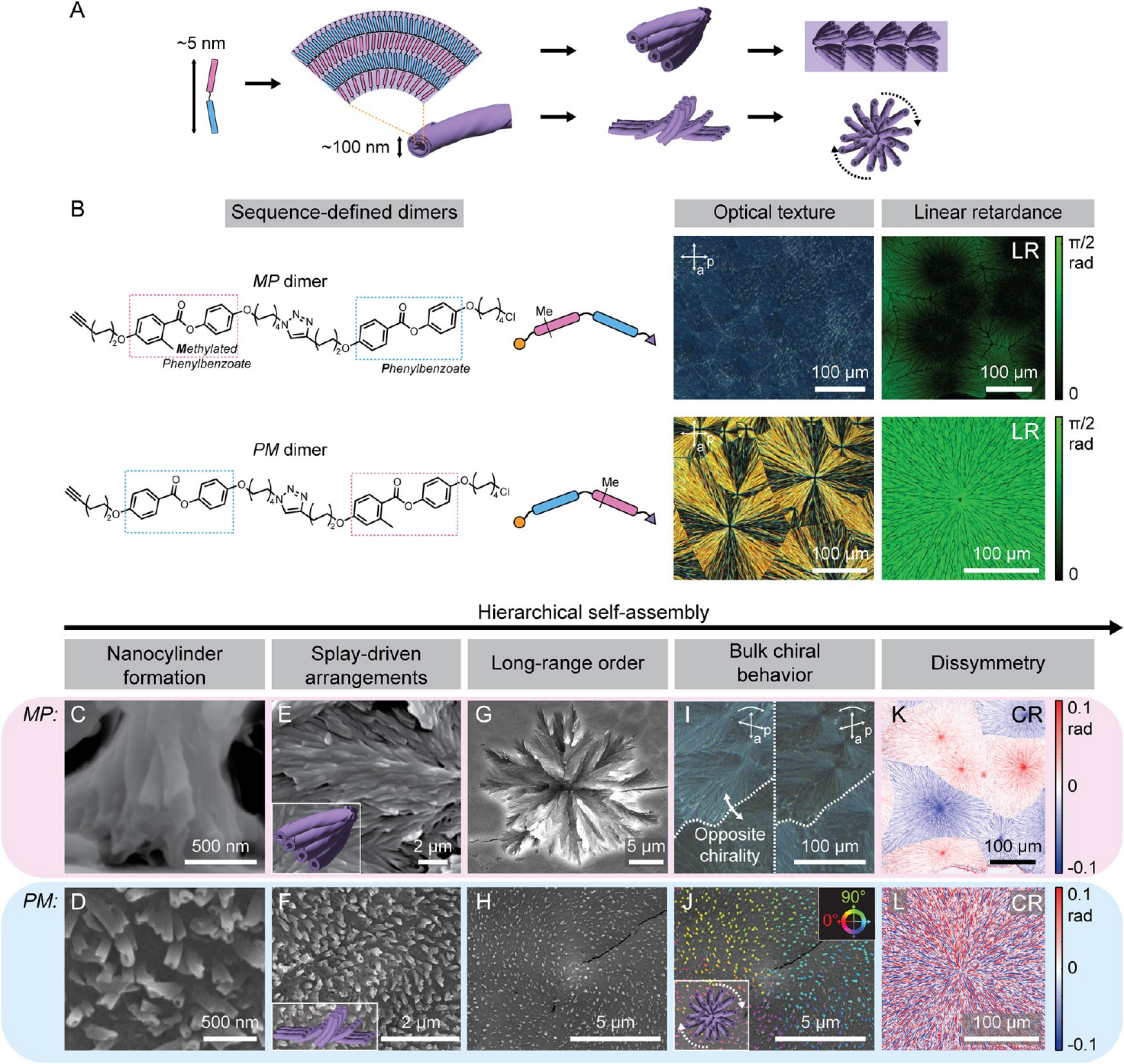
Multiscale self-assembly of nanocylinder-forming mesogenic heterodimers. (A) Schematic depicting the hierarchical self-assembly of dimers into nanocylinders composed of smectic layers, which then adopt different arrangements, some of which are sense-specific. (B) Chemical structures of *MP* (top left) and *PM* (bottom left) dimers, with schematic representations shown to the right of the chemical structures. The methyl substituent position is highlighted by vertical lines. These dimers exhibit differing optical textures, shown by micrographs captured between crossed polarizers (POM) and linear retardance micrographs of the crystalline phase of *MP* (top right, brightness and contrast of POM enhanced for visibility) and *PM* (bottom right). (C,D) Scanning electron micrographs (SEM) of curved nanocylinders formed by the (C) *MP* and (D) *PM* dimers. (E,F) SEM and schematics (inset) of (E) periodic splay and twist arrangements adopted by nanocylinders formed by the *MP* dimer and (F) periodic splay of nanocylinders formed by the *PM* dimer. (G–H) SEM of spherulite centers illustrating long-range order of nanocylinders in (G) *MP* and (H) *PM*. (I,J) Nanocylinder arrangements are associated with bulk chirality observed as (I) optical rotation in *MP*, shown by POM with slightly decrossed polarizers, and (J) the cyclic sense of the nanocylinder arrangement at the spherulite surface in *PM*, shown by SEM and a schematic (inset). The color of each nanocylinder in **J** corresponds to the orientational angle of the projection of the nanocylinder onto the surface plane (legend shown in top right). (K,L) Circular retardance (CR) micrographs indicate (K) an ensemble of sense-specific spherulites (each spherulite is either dextro or levorotatory) in *MP* and (L) an absence of CR-specific spherulites in *PM*.

## Results and Discussion

### Supramolecular Chirality Arises from Hierarchical Self-Assembly of Bent Mesogenic Dimers

The self-assembly of mesogenic dimers depends on the precise combination of the mesogenic core, spacer unit, and tail groups.^76^ Here, we compare two sequence-defined mesogenic dimers with identical spacer and tail groups that differ only in the position of a methyl substituent on a phenylbenzoate-based mesogenic core (chemical structures shown in Figure 1B, synthetic details in Section S1 and Schemes S1–S7, and chemical characterization in Section S2 and Figures S1–S25). These dimers were synthesized by orthogonal protection–deprotection click chemistry, which enables precise control over the sequence of monodisperse dimers and longer oligomers.^77,78^ The dimers studied here, schematically represented in Figure 1B, are respectively denoted *MP* and *PM*, labeled first by the mesogen closest to the alkynyl terminus of the asymmetric dimer, where “*P*” denotes the “phenylbenzoate” monomer and “*M*” denotes the “methyl-substituted” monomer.

The ensemble-averaged conformation arising from the specific dimer molecular structure dictates the self-assembly. When the mesogens are collinear, the dimers behave similarly to a calamitic liquid crystal.^76^ When a bent intermesogen geometry is adopted, often quantified by a bend angle, mesogenic dimers are considered flexible bent-core mesogens. The conformation (collinear or bent) is typically considered to be determined by the spacer unit between mesogens.^79^ In “classical” mesogenic dimers (such as the canonical CB*n*CB family of molecules), the intermesogen geometry can be predicted from the parity of the methylene units in the linker.^36^ For the dimers studied here, the rigid 1,2,3-triazole group in the linker prevents us from applying conventional odd–even rules to infer the conformation. Therefore, calculations are necessary to predict the molecular conformation.

To assess whether triazole-containing linkers influence the manifestation of odd–even effects, bend-angle geometries were analyzed from conformational ensembles generated using density functional theory calculations and a one-dimensional rotational isomeric state (RIS) approximation (Section S3). Section S3 discusses the scope, limitations, and additional considerations involved in relating these isolated-molecule conformational ensembles to crystalline-phase behavior. Similar approaches have been applied to estimate the bend angle of different cyanobiphenyl dimer series. However, these methods often rely on semiempirical or lower levels of theory and consider only the aliphatic segments of alkylbiphenyl liquid crystals in the nematic phase.^80^ The dimers studied here contain a flexible linker with a triazole ring and phenylbenzoate mesogenic cores that introduce additional complexity when sampling the conformational space. To address this complexity, we identified eight fragments of interest (Figure S26) in an alkyl-linked phenylbenzoate liquid crystal (Figure S27) as well as the triazole-linked dimer (Figure S28). This approach allows for more comprehensive sampling of the conformational space and demonstrates two additional degenerate minima on either side of the triazole fragments when sampled at a low temperature (Figure S26D,E). In the alkyl-linked phenylbenzoate mesogen we observe a clear odd–even effect in the bend angle (Figure S27A) caused by the geometry induced by the single minima of the alkyl linkers. The odd–even behavior is more complex in the triazole-containing linkers, as the triazole can adopt four possible conformational states, which are sorted into distinct plots in Figure S28A–D. These calculations indicate that dimers with different triazole conformers can exhibit different ranges of possible bend angles and in some cases inverted odd–even effects. Of course, understanding whether such conformational ensembles manifest odd–even effects in self-assembly would require the synthesis and experimental study of these additional molecules.

In previous molecular dynamics (MD) simulations of the nematic (*N*) phase of the triazole-linked dimers studied here, two conformations dominate: a smaller population adopting a collinear hairpin conformation, and a second population adopting a bent intermesogen geometry with local energy minima at bend angles of ∼140°–150°.^81^ Although the conformations in the nematic phase cannot be directly applied to a crystalline state, the MD simulations suggest that *MP* and *PM* energetically prefer to adopt a bent conformation, and this preference to adopt a bent intermesogen geometry likely induces frustration that favors the curved microstructures adopted in the crystalline phase of both dimers.

Upon crystallization, both *MP* and *PM* exhibit different optical textures (Figure 1B) and assemble into nanocylinders (cylindrical curvature), which can be visualized through freeze-fracture scanning electron microscopy (SEM) (Figure 1C,D). Such cylindrical curvature is similar to the larger scrolled sheets observed in the homodimer (*P*_2_) described previously (multiple 100s nm outer diameter for *P*_2_ vs 100–200 nm outer diameter for *MP* and *PM*).^82^ However, despite similar chemical structure and nanocylinder formation, the arrangement of the nanocylinders for *MP* and *PM* diverges substantially (Figure 1E–H), resulting in their distinct chiroptical responses (Figure 1I–L). *MP* forms an ensemble of spherulites, some of which are dextrorotatory and others of which are levorotatory (Figure 1I, K). The circular retardance (CR) signal is largest at the nuclei where many fibrils (in this case, nanocylinders) grow normal to the substrate.^83^ *PM* displays CR that arises from a disorganized misoverlap of highly linearly anisotropic regions of nanocylinders (Figure 1L). Dissymmetry of the *PM* nanocylinders manifests in a cyclic sense by SEM, but this sense varies among spherulites (Figure 1J). By comparing the hierarchical assembly of these two dimers, this work aims to articulate how changes in molecular structure lead to divergence in long-range organization.

### *MP* and *PM* Heterodimers Exhibit Divergent Optical Textures

In the *MP* dimer, the methyl group is positioned further (in terms of bond lengths) from the central flexible spacer compared to that in *PM* (see schematics in Figure 2A,B). Atomistic MD simulations suggest that in the *N* phase, the position of the methyl group does not substantially affect the minimum energy bend angle but does impact the energy landscape of torsional angles formed by the mesogenic core.^81^ While a wide range of mechanisms can influence crystallization, we anticipate that, within a tightly packed crystalline matrix, the position of the methyl substituent can dictate differences in intermesogen geometry and molecular-level packing.

**Figure 2:**
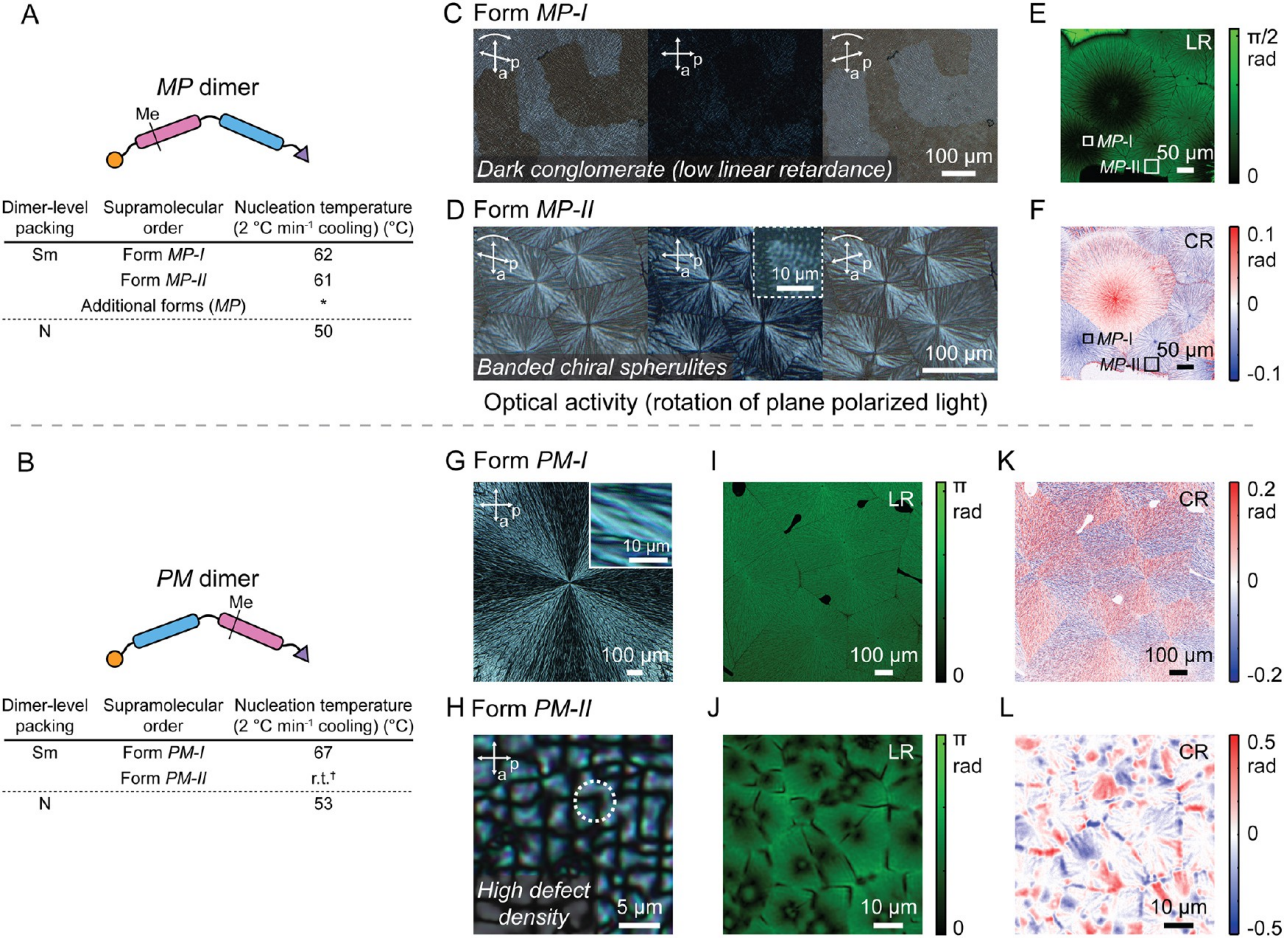
Phase transitions and polarized optical micrographs of heterodimers. (A, B) Nucleation temperatures (obtained by a time series of polarized optical micrographs during 2 °C min^–1^ cooling) from the *Iso* melt of phases with smectic or nematic ordering of dimers formed by the (A) *MP* and (B) *PM* dimers. *PM*-II nucleation occurs at room temperature in a quenched nematic. Schematic representations of the molecular structures are presented for (A) *MP* and (B) *PM*. (C, D) Optical micrographs recorded between crossed polarizers (POM) and decrossed polarizers in opposite directions of (C) *MP*-I and (D) *MP*-II. The emergence of alternating light and dark regions upon decrossing the polarizers is characteristic of optical rotation, indicating the presence of homochiral domains of opposite chiral sense. *MP*-II additionally exhibits concentric banding (D, inset). (E) Linear retardance (LR) and (F) circular retardance (CR) micrographs of *MP*-II spherulites and spherulites nucleated as *MP*-I that transition to *MP*-II. *MP*-I exhibits lower LR than *MP*-II, and both form sense-specific spherulites as shown by CR (regions for calculations shown by squares). (G, H) POM of (G) *PM*-I (inset: The banding in *PM*-I is observed as radial rays) and (H) *PM*-II, which exhibits a high defect density, including right-angle defect lines (dotted white circle). (I, J) LR micrographs of (I) *PM*-I and (J) *PM*-II show larger variations in LR in *PM*-II. (K, L) CR micrographs of (K) *PM*-I and (L) *PM*-II. All POM images were collected for samples sandwiched between glass slides with ∼5 μm spacers, except **G**, which were thin cells without spacers, and the inset of **G**, which was prepared between indium tin oxide coated glass without spacers and freeze-fractured prior to imaging. The * symbol in **A** indicates that *MP* exhibits other spherulite types each with their own nucleation temperature, referred to as *MP*-III and other *MP* crystalline forms as shown in Figures S37 and S38. The † symbol in **B** indicates that *PM*-II nucleates from the *N* at room temperature.

To understand the effect of the methyl substituent, we first compare a methylated monomer (*M*_1_) and homodimer (*M*_2_) to their nonmethylated equivalents (*P*_1_ and *P*_2_ respectively).^82^ A comparison of their phase behavior during 2 °C min^–1^ cooling reveals that the methyl substituent suppresses the isotropic-to-nematic transition temperature (*T*_ni_) and crystallization temperatures. In the case of the monomers, methylation decreases *T*_ni_ from 64 °C (*P*_1_) to 8 °C (*M*_1_), while the crystalline nucleation temperature (*T*_nucleation_) drops from 38 °C (*P*_1_) to 14 °C (*M*_1_) (Figure S29). The effect of the methyl group is more substantial for the homodimers; during 2 °C min^–1^ cooling, only crystallization is observed in *P*_2_, while only an isotropic-to-nematic (*Iso*-*N*) transition is observed in *M*_2_ (*T*_ni_ = 23 °C). An isothermal hold (∼1 h) at or above room temperature after cooling is required for *M*_2_ to crystallize (Figure S30). The transition temperature suppression and change in the crystallization kinetics relative to *T*_ni_ may be due to reduced packing efficiency induced by the methyl substituent. Both homodimers, *M*_2_ and *P*_2_, display chaotic CR signal that is not indicative of sense-specific spherulites (Figures S31 and S32). However, when isothermally crystallized at 52 °C, *P*_2_ shows limited formation of spherulites with periodic (Figure S32E,F) and aperiodic (Figure S32G,H) banding arising from a twisted microstructure (Figure S33), where individual spherulites may nucleate with heterochiral twists.^84^

In the heterodimers *MP* and *PM*, we explore the role of the methyl group by examining the phases and crystalline forms that develop during cooling (2 °C min^–1^). Under these conditions, both heterodimers crystallize directly from the melt into spherulites. Within the undercooling window, the remaining isotropic (*Iso*) material forms a metastable (“monotropic”) *N* phase (Figure 2A,B). *T*_nucleation_ and *T*_ni_ (and melting temperatures for *MP*-I and *MP*-II only) obtained from a time series of polarized optical micrographs enables a visual deconvolution of crystallization and *Iso*-*N* transitions. In contrast, using differential scanning calorimetry, crystallization and *Iso*-*N* transition peaks may be overlaid and difficult to deconvolute (Figures S34–S36). The small difference in the *T*_ni_ between *PM* and *MP* (Δ*T*_ni_ = ± 3 °C) reflects the conformational similarities between these two dimers in their *Iso* and *N* phases and suggests that the differences between the crystalline phases of these two dimers arise primarily from packing effects on crystallization.

Despite similar *T*_ni_’s, heterodimers crystallize into distinct spherulites that differ in supramolecular structure and optical properties (Figure 2C–L). The crystalline phase of *MP* exhibits multiple spherulite types (Figures 2C–F, S37 and S38), of which this study will focus on the two most commonly observed forms when subjected to 2 °C min^–1^ cooling. (Although we refer to different spherulite types as “crystalline forms,” they are not strictly polymorphs.) These two spherulites, referred to as Form *MP*-I and Form *MP*-II (Figure 2A, **C–F**), exhibit higher *T*_nucleation_. Since *T*_nucleation_ and the melting temperatures (*T*_m_) during 2 °C min^–1^ cooling and heating, respectively, are similar between *MP*-I (*T*_nucleation_ = 62 °C, *T*_m_ = 84 °C) and *MP*-II (*T*_nucleation_ = 61 °C, *T*_m_ = 85 °C) (Figures 2A, S39), we have not been able to fully isolate either by isothermal crystallization. Note that in some cases we have isothermally crystallized *MP* at temperatures above the *T*_nucleation_ measured during 2 °C min^–1^ cooling to obtain a larger fractional area of *MP*-I. Furthermore, during both isothermal crystallization and cooling at a fixed rate, a spherulite can nucleate as *MP*-I and, during growth, transition to *MP*-II (Figure S40). *MP*-I does not cross-nucleate on *MP*-II.

As the molecules of the same dimer would be expected to have comparable polarizabilities, the optical features of these crystalline forms enable inferences about differences in molecular arrangement. Dendritic *MP*-I spherulites have an average linear retardance (LR) of |0.039| rad (Figure 2E) for films ∼5 μm in thickness. An ensemble of dextrorotatory and levorotatory spherulites are revealed by bright and dark regions alternating with the sense of slightly decrossed polarizers (Figure 2C). Individual spherulites are homochiral possessing an average CR with magnitude of ±0.038 rad (Figure 2F). “Dark conglomerate” homochiral domains were reported previously for rigid bent-core mesogens,^46,47,85,86^ mesogenic trimers,^48,56,87,88^ and isotropic liquids.^18,89^ Although the term “dark conglomerate” has been used to refer to a specific LC phase,^47^ in this work we use it more broadly to describe all optical textures with minimal LR and chiroptical domains. Similar to *MP*-I, *MP*-II exhibits an ensemble of spherulites of opposite CR, but with lower average CR of ±0.019 rad (Figure 2F) and a higher average LR of |0.30| rad (Figure 2E). This optical behavior has previously been observed in HNFs formed by both chiral^40,90^ and achiral^28,91^ rigid bent-core mesogens. In both *MP*-I and *MP*-II, while each domain is optically homochiral, there is an equal probability of forming right and left-handed domains.

*MP* also crystallizes into less common spherulite types with higher LR and a chaotic CR signal indicating a lack of sense-specific spherulites. These spherulite types include *MP*-III (average LR of |0.41| rad), which has a similar nucleation temperature to *MP*-I and *MP*-II, and multiple distinct forms with nucleation temperatures below the *T*_ni_, which we collectively refer to as “other *MP* crystalline forms” (Figures S37 and S38). The other *MP* crystalline forms are often observed as the outer rings of *MP*-I and *MP*-II spherulites following 2 °C min^–1^ cooling and can be directly nucleated below the *T*_ni_. Unlike *MP*-I and *MP*-II, which form nanocylinders of consistent dimensions when examined through freeze-fracture SEM, the higher LR spherulites display nanocylinders with wider ranges of outer diameters, with all except *MP*-III additionally showing a surrounding microstructure without defined features (Figure S41). These featureless regions can result from different fracture planes with respect to *MP*-I and *MP*-II. The spherulite types of *MP* can be easily distinguished with a first order red plate waveplate. *MP*-I spherulites are negative (the larger refractive index is tangential) and *MP*-II spherulites are positive (the larger refractive index is radial), while *MP*-III and certain other *MP* forms are negative (Figure S42).

The *PM* dimer exhibits two crystalline forms with optical textures distinct from each other and from *MP*. Form *PM*-I nucleates at a higher temperature than the crystalline phases of *MP*, while Form *PM*-II nucleates at room temperature from the metastable *N* (Figure 2B, G–L). These two forms (abbreviated hereafter as *PM*-I and *PM*-II) can be respectively isolated by isothermal crystallization above the *T*_ni_ or by quenching to room temperature to nucleate crystals directly from the *N* melt. *PM*-I forms rays originating at the spherulite centers occasionally separated by disclination lines (Figure 2G–I). Notably, the spherulites of *PM* do not exhibit optical rotation systematically upon decrossing the polarizers and their chaotic CR signal does not correspond to sense-specific spherulites (Figure 2K).

*PM*-II, which displays an optical texture similar to that of a pseudofocal conic structure,^92^ exhibits larger variations in LR than *PM*-I and a high defect density (Figure 2H–J). The large number of defects originates from the small size of the spherulites, each of which are marked by a defect with a strength of +1 at the nucleation center. At the point where three or more spherulites converge, a defect with a strength of −1/2 is observed. The magnitude and sign of each defect type can be mapped by observing the sample between rotating polarizer and analyzers (Figure S43).^93^ Unusually, the domains between spherulites are not delineated by strict boundaries. The −1/2 defects appear as right-angle dark brushes. This defect type requires a large bend or splay in the director field (as illustrated in the model director field in Figure S43A). *PM*-II exhibits CR signal that is characteristic of spherulites with overlapping nanocylinders at different heights at boundaries (Figure 2L). The CR sense is determined by the angle with which the misoriented nanocylinders overlap at the boundaries of spherulites.^94^ We next seek to articulate the hierarchically ordered microstructures of these self-assembled systems, thereby elucidating how small changes in molecular structure and crystallization conditions lead to this wealth of unusual forms.

### Dimer Sequence Dictates Smectic Layer Curvature within Nanocylinders

The local molecular packing within the crystalline phases can be analyzed through X-ray scattering on “bulk” (∼0.5 mm thick) samples (Figures 3A,B and S44–S47). For *MP*, the isothermally crystallized bulk sample contains an uncontrolled mixture of *MP*-I, *MP*-II, and *MP*-III. To ensure that the bulk X-ray scattering profile reflects both *MP*-I and *MP*-II, the measurement is benchmarked against localized X-ray scattering profiles of a single optically verified spherulite type in thin (<5 μm) isothermally crystallized samples, revealing negligible differences between the molecular packing of *MP*-I and *MP*-II (Figure S48). In *PM*, the bulk X-ray scattering profile reflects the molecular packing of *PM*-I (Figure S49). In comparison to *PM*-I, the lowest *q* peak of *PM*-II is broader and shifted to a lower *q*-value, suggesting that kinetic trapping leads to lower smectic layer coherence. Aside from the shift in this peak’s *q*-value and the overall broadness of peaks in the spectrum, the features observed in the X-ray scattering spectra are similar between *PM*-I and *PM*-II.

**Figure 3:**
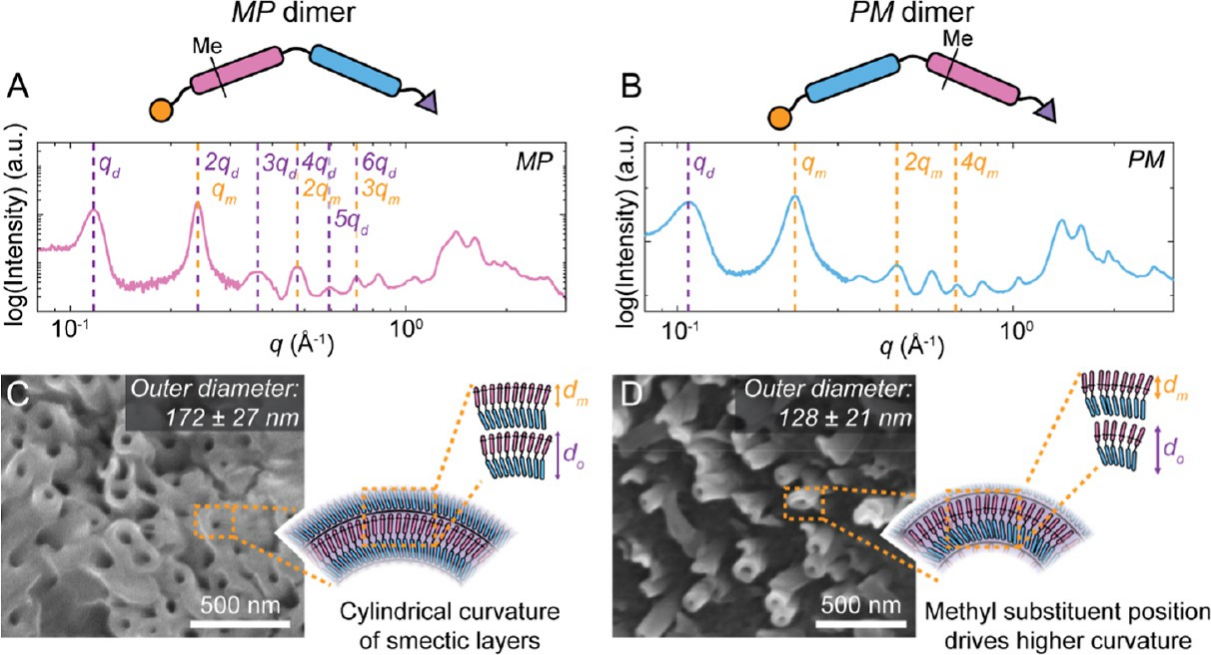
Molecular packing and nanocylinder formation of mesogenic heterodimers. (A, B) Azimuthally integrated 1D X-ray scattering spectra of bulk samples (containing multiple supramolecular order types) of (A) *MP* isothermally crystallized at 67 °C and (B) *PM* isothermally crystallized at 58 °C. Scattering peaks associated with smectic layer spacing of dimers are indicated by purple dotted lines (*q*_d_), while scattering peaks associated with monomer unit spacing along the smectic layer normal are indicated by orange dotted lines (*q*_m_). (C, D) Top-down scanning electron micrographs of nanocylinders and associated average nanocylinder outer diameters in (C) a *MP*-II spherulite and (D) a *PM*-I spherulite formed by 2 °C min^–1^ cooling from the *Iso* melt. Schematics are presented for the proposed arrangement of dimers and differing nanocylinder outer diameters in (C) *MP* and (D) *PM*.

The bulk scattering profiles of the heterodimers are characterized by smectic layers with crystalline in-plane ordering. The lowest *q*-value peak (*q*_d_) is associated with a *d*-spacing corresponding to the total molecular contour length of the dimer (*d*_d_), which can be estimated using a previously reported 2D geometric approximation.^82^ The organization of dimers into layers is further supported by a first-order peak (*q*_m_) with a *d*-spacing corresponding to the length of a monomer unit (*d*_m_). These two peaks, for which *q*_m_ matches or exceeds the intensity of *q*_d_, are known to occur in a lamellar organization of dimers with a bent intermesogen geometry. When the lengths of the flexible tails and linker are approximately equal, synchronized electron density fluctuations associated with the length of a dimer and a monomer unit can be observed.^42^ The high-*q* region of the X-ray scattering profiles of the heterodimers studied here exhibits sharp high-intensity peaks, indicating crystalline in-plane ordering of monomer units within a layer.

While the presence of the methyl substituent plays an important role in determining the molecular packing of each dimer, the steric mismatch between the two constituent monomers, dictated by the methyl substituent’s position, also affects the layer spacing. The general pattern of scattering peaks is comparable between *MP* and *PM*. They primarily differ in their smectic layer spacing (represented by *d*_m_ and *d*_d_). The values of *d*_m_ and *d*_d_ are both slightly larger for *PM* (*d*_m_ = 2.8 nm, *d*_d_ = 5.8 nm) than for *MP* (*d*_m_ = 2.6 nm, *d*_d_ = 5.4 nm) (Figure 3A,B, Table S1). A possible reason for the larger layer spacing in *PM* is that positioning the methyl substituent closer to the central flexible spacer results in a more extended average chain conformation (i.e., larger intermesogen bend angle). A similar effect has been observed in rigid bent-core mesogens stereoisomers with a methyl substituent on flexible tails, where the substituent position affects the core-chain angle.^53–55^ However, in the system studied here, MD simulations have suggested that the position of the methyl substituent does not significantly change the conformational ensemble of the dimers in their *N* phase.^81^ In the crystalline phase, *inter*molecular rather than *intra*molecular interactions may play a role in determining the average chain conformation and resultant molecular packing. The role of the methyl substituent in increasing the intermesogen bend angle and thereby smectic layer spacing is supported by the smaller layer spacing of *P*_2_, which has no methyl substituents (*d*_d_ = 5.3 nm).^82^ The fully methylated systems (*M*_1_ and *M*_2_) possess both the *q*_m_ peak (and *q*_d_ in *M*_2_) and additional peaks between *q*_m_ and 2*q*_m_ not observed in their nonmethylated or heterodimer counterparts (Figure S50). Additionally, the smectic layer spacing of *M*_2_ is intermediate between that of *PM* and *MP* (*d*_d_ = 5.5 nm), rather than being the expected largest layer spacing. We attribute this deviation to the decreased steric mismatch between the two monomers of *M*_2_ relative to the heterodimers, indicating more complex factors at play in determining the molecular packing.

In addition to increasing the layer spacing, the methyl substituent dictates smectic layer coherence and in-plane crystalline order. Compared to *P*_2_, which exhibits sharp *q*_d_ and *q*_m_ peaks coupled with strong in-plane crystalline ordering, the less ordered heterodimers exhibit broader peaks (Figure S50, Table S1). Between the two heterodimers, the centrally positioned methyl group in *PM* has a larger steric effect and thus a greater impact on the crystalline order. Accordingly, the X-ray scattering spectrum for *PM* exhibits fewer higher-order reflections and greater peak breadth compared to *MP*, as indicated by the larger full-width-at-half-maximum values for the primary *q*_d_ and *q*_m_ peaks (Figure 3A,B, Table S1). The smectic layer coherence and the intermesogen bend angle may also be correlated, as smaller bend angles can reduce rotational degrees of freedom and thereby induce stronger smectic ordering.^95^ Additionally, *P*_2_ shows a greater number of sharp peaks in the high-*q* region, corresponding to greater short-range in-plane crystalline order. Differences in the in-plane crystalline order is supported by calorimetry. From differential scanning calorimetry during 2 °C min^–1^ cooling from the *Iso* melt, *MP* and *PM* have comparable melting temperatures (*T*_m_ of *MP* = 82.6 °C, *T*_m_ of *PM* = 82.9 °C) and specific melting enthalpies (77.6 J g^–1^ for *MP*, 74.7 J g^–1^ for *PM*), while the values for *P*_2_ (*T*_m_ = 110.1 °C, specific melting enthalpy of 86.7 J g^–1^)^82^ are much higher (Figure S51). Thus, the methyl substituent increases the enthalpy of the heterodimers relative to *P*_2_. The trend in *T*_m_ continues with *M*_2_, which has two methyl substituents that further suppress the transition temperature (*T*_m_ = 80.8 °C) (Figure S51). However, the specific melting enthalpy of *M*_2_ (82.2 J g^–1^) falls between that of *P*_2_ and the heterodimers.

The influence of the methyl substituent on molecular packing is also key to dictating the microstructural curvature and resultant nanocylinder dimensions. We propose that dimers exhibiting a bent intermesogen geometry pack within smectic layers. As previously explored for *P*_2_,^82^ within a nanocylinder cross-section, the dimers align with their long axis along the nanocylinder radius (Figure 3C,D). This arrangement of dimers is verified in *MP*-II by the 2D X-ray scattering pattern of a localized region of a spherulite in which the nanocylinders are known to orient along the spherulite radius (Figure S52). In *P*_2_, the bend curvature of smectic layers results in the formation of scrolled sheets with variable outer diameters on the scale of several hundred nanometers. In comparison, the heterodimers form nanocylinders with much smaller average outer diameters (172 ± 27 nm for *MP* and 128 ± 21 nm for *PM*) (Figure 3C,D). We attribute the narrower range of curvature in the heterodimers to the methyl substituent introducing additional intermolecular splay within a layer (schematic in Figure 3C,D). Note that we assume that the *M* monomers on adjacent heterodimers are aligned within the layer plane, which enables alignment of flexible tails with the same lengths and functional groups and flexible spacers with the same number of carbons on each side of the triazole ring. Under this proposed molecular packing scheme, the intralayer splay induced by the methyl substituent restricts the layers to high-curvature deformations. Given that the curvature scales inversely with the diameter of the nanocylinders, the induced splay limits radial growth, resulting in nanocylinders of relatively uniform size. In the absence of this additional intermolecular splay, *P*_2_ can access a broad range of curvatures and therefore form scrolled sheets with variable larger outer diameters. We further compare the microstructures formed by the heterodimers to *M*_2_, which exhibits cylindrical curvature but does not form nanocylinders that are as regular as those in *MP*-I, *MP*-II, and both crystalline forms of *PM* (Figure S53). The cylinders in *M*_2_ (∼200 nm or greater in outer diameter) are larger than those of the heterodimers but smaller than the *P*_2_ scrolls. A possible reason for this intermediate outer diameter is the comparable size of the two constituent monomers of *M*_2_ relative to the heterodimers, thereby decreasing the intermolecular splay within a layer.

Steric differences defined by the methyl substituent position also play a role when comparing the nanocylinder dimensions of the heterodimers to each other. Placing the methyl substituent closer to the central flexible spacer, as in *PM*, is expected to increase the effective splay, thereby increasing the curvature and reducing the average outer diameter. The inner diameter is more affected by the methyl substituent position, with the inner diameter of *PM* (37 ± 11 nm) almost half that of *MP* (56 ± 14 nm). As a result, the nanocylinder wall thickness is greater for *MP* (58 ± 21 nm) than for *PM* (45 ± 16 nm). Taking *d*_d_ as the layer spacing, we estimate that the nanocylinders comprise 11 ± 4 smectic layers in *MP* and 7 ± 3 smectic layers in *PM*. As such, the methyl substituent position and resultant steric differences influence the maximum and minimum layer curvature and ultimately the nanocylinder dimensions. These disparities in nanocylinder dimensions may be the origin of differences in nanocylinder arrangements between *MP* and *PM*.

### *MP* Dimer Nanocylinders Arrange into Chiral Structures Exhibiting Optical Rotation

Optical differences between *PM* and *MP* likely arise from the supramolecular assembly of these heterodimers. The most striking difference between the two heterodimers is that some *MP* spherulite forms (*MP*-I and *MP*-II) display spherulite-specific optical activity, whereas no systematic chiroptical behavior is discernible in *PM*. Given that *MP* and *PM* both form similar nanocylinders and present comparable X-ray scattering patterns, the differences remain a puzzle.

We propose that the observed optical rotation in *MP*-I and *MP*-II may arise from a chiral hierarchical arrangement of nanocylinders. A spherulite consisting of nanocylinders in twisted bundles with the same handedness can produce the optical rotation observed in the crystalline forms of *MP*. An example of this mechanism has been demonstrated in semicrystalline polymer spherulites, where a twisted macroscopic arrangement of lamellae can produce circular birefringence.^96^ Similarly, in HNF systems, optical rotation has been attributed to a secondary twist of nanofilaments, in which the orientation of stacked nanofilaments is offset by a constant angle.^40,97^ This secondary twist is also proposed as a source of the blue structural color exhibited by HNF systems.^97^ Notably, *MP*-I and *MP*-II also exhibit blue-light scattering (Figure S54).

The hierarchical nanocylinder arrangements can arise from the curved, rod-like shape of the nanocylinders. For the curved nanocylinders to uniformly fill space, the bend deformation present in their shape must be accompanied by additional deformations during their growth (i.e., twist and/or splay) and secondary nucleation events to insert new nanocylinders. An analogy can be made with symmetry-breaking in colloidal systems, as curved and bent colloidal rods can self-assemble into nonuniaxial nematic phases^33^ or smectic phases resembling those formed by bent-core mesogens.^20^ The nanocylinders here grow cooperatively from a nucleation center in the melt to form spherulites and therefore adopt different assembly mechanisms than discrete colloidal rods that do not grow as spherulites. Nevertheless, the tendency of the nanocylinders to bend during growth may induce shape effects on their packing, similar to those occurring in systems of curved and bent colloidal rods. Similar hierarchical assembly of nanocylinders into arrangements such as braids and nest-like structures has been observed previously in a system of HLNCs formed from chiral rigid bent-core mesogens.^53^

During spherulite growth, radial splay of nanocylinders is required to fill space as the spherulite diameter increases. Under these conditions, a chiral arrangement of nanocylinders may be adopted if, due to the curved shape of the nanocylinders, the radial splay of nanocylinder orientations is coupled to a twist deformation. SEM of *MP*-I and *MP*-II show that the nanocylinders formed by *MP* assemble into structures resembling twisted bundles (Figure 4A). Due to the limitations of standard cross-sectional SEM analysis imposed by preferential fracture planes, it is challenging to definitively assign a twist sense to each bundle or quantitatively determine a twisting pitch. Nevertheless, this hypothesis is supported by the absence of clear twisted bundles in *MP*-III and the other *MP* crystalline forms (Figure S41), which do not exhibit sense-specific spherulites (Figure S38). The crystalline forms of *PM* also do not exhibit any coherent optical rotation but rather a chaotic texture that arises from overlapping nanocylinders (Figures 1L and 2K); no twisted bundles are observed by SEM (Figure 5A). The formation of twisted bundles here can be compared to the literature; in a system of folded chiral azobenzene dyads, enantiopure nanotubes exhibited tighter packing and resultant internal strain relieved by helical coiling of nanotubes compared to racemic nanotubes with a greater degree of steric mismatch and no observed coiling.^67^ In our system, it is possible that the key difference in assembly of *MP* versus *PM* spherulites is simply the shape of the nanocylinders themselves: slight differences in smectic layer deformation may cause *MP* nanocylinders to adopt a geometry that drives assembly into twisted chiral domains, whereas *PM* nanocylinders do not. However, direct measurement of the long-axis curvature of nanocylinders is not directly accessible from cross-sectional SEM.

**Figure 4:**
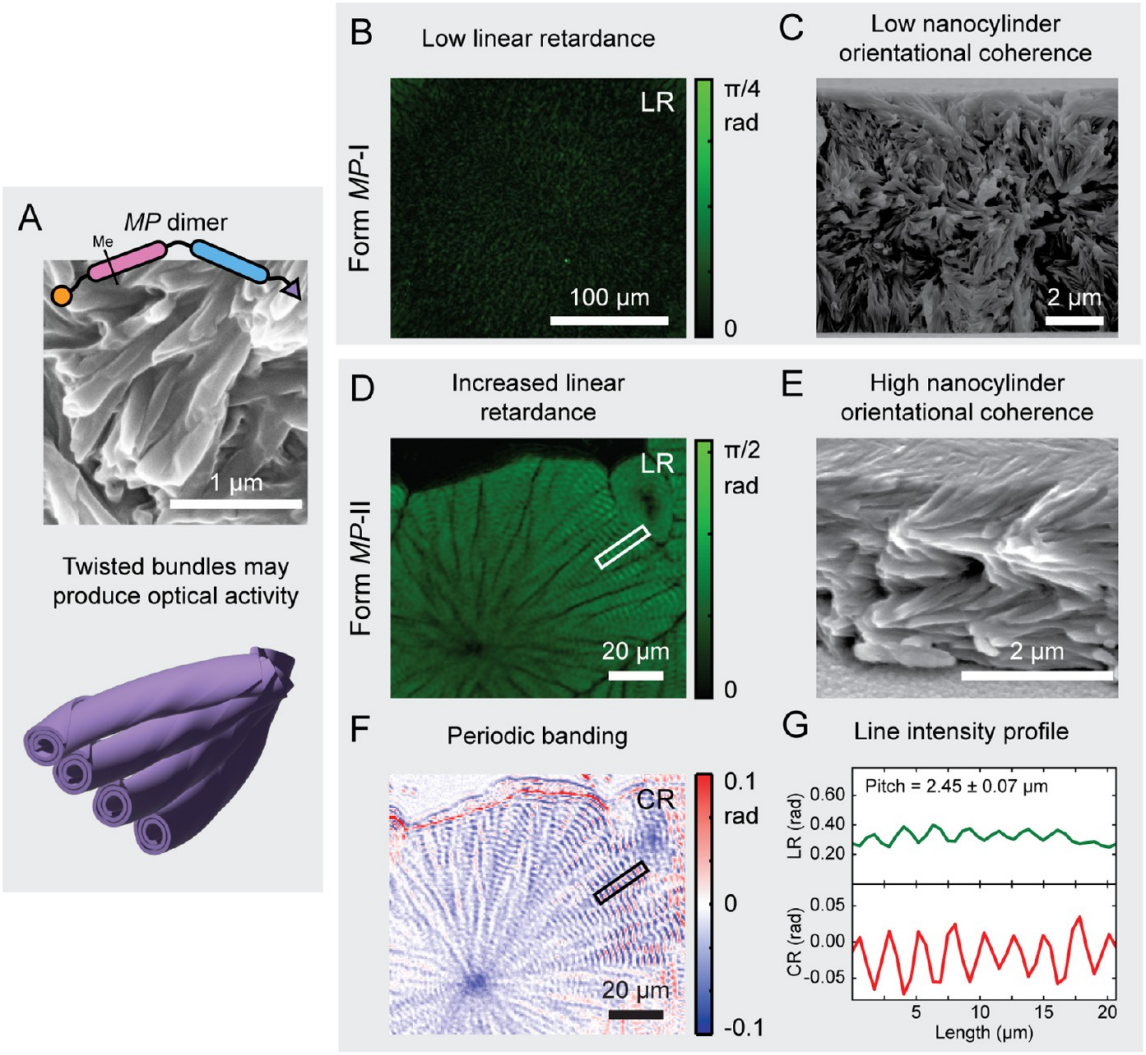
Nanocylinder arrangements correlated with the optical texture of *MP*-I and *MP*-II. (A) Scanning electron micrograph (SEM, top) and schematic (bottom) of a nanocylinder bundle in *MP*-I. (B) Linear retardance (LR) micrograph and (C) SEM of *MP*-I. Low LR corresponds to low nanocylinder orientational coherence in *MP*-I. (D) LR micrograph and (E) SEM of *MP*-II indicate higher LR corresponding to higher nanocylinder orientational coherence. (F) Circular retardance (CR) micrograph of *MP*-II showing periodic banding. (G) Line intensity profile of LR (top) and CR (bottom) of the periodic banding in *MP*-II. The region used to calculate the line intensity profile is shown by the white box in **D** and the black box in **F** for LR and CR, respectively. The SEM in **A** was collected using a Verios 460 Extreme High Resolution SEM.

**Figure 5:**
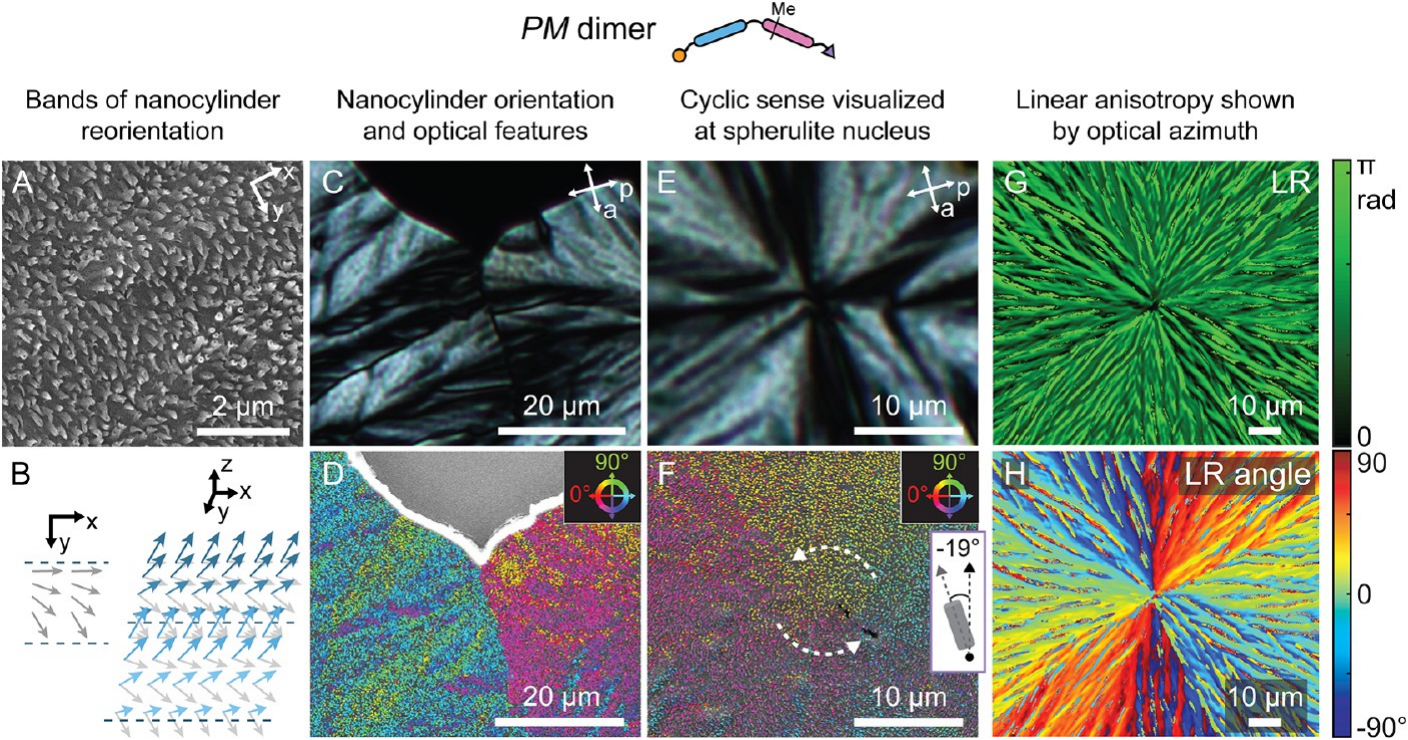
Nanocylinder arrangements and optical banding of *PM*-I. (A) Scanning electron micrograph (SEM) depicting reorientation of nanocylinders. (B) Schematic demonstrating periodic nanocylinder splay (represented by arrows) separated by domain walls (dotted lines). The projection into the 2D surface plane is shown on the left. On the right, the reorientation domains are shown in 3D, where the gray arrows represent the 2D projection. (C) Polarized optical micrograph (POM) of the domain boundary between two spherulites of *PM*-I. (D) SEM of the domain boundary shown in **C**. Nanocylinders are overlaid with labels with colors corresponding to the orientational angle made by the projection of the nanocylinder orientation onto the 2D surface plane with respect to the horizontal (legend in top right). The spherulite exhibits an azimuthal directionality of nanocylinders. (E) POM of the spherulite center of *PM*-I. (F) SEM of the spherulite center shown in **E**. Nanocylinders are overlaid with colored orientation labels as in **D**. The nanocylinders are oriented at an average angle of 19° ± 41° anticlockwise with respect to the radial vector, as demonstrated by the schematic on the left. The anticlockwise direction around the center of this spherulite is illustrated by the dotted white arrows. (G) Linear retardance (LR) micrograph of a spherulite center (different from that shown in (E,F). (H) LR azimuthal orientation of the spherulite shown in **H**. The larger refractive index is plotted clockwise from the horizon. While the nanocylinder orientation at the spherulite surface possesses a sense (F), the LR does not possess a significant sense (H). The polarized optical micrographs in **C** and **E** were collected from samples cooled at a rate of 2 °C min^–1^ from the isotropic melt in a sandwich cell of uncontrolled thickness, which were freeze-fractured and imaged by SEM prior to optical image collection. The brightness and contrast have been modified for **C** and **E** for improved visibility of the optical texture.

### Splay-Driven Nanocylinder Arrangement is Modified by Geometric Confinement

In addition to an apparent correlation between optical rotation and a twisting nanocylinder arrangement in *MP*, differences in nanocylinder arrangement between the different forms of *MP* and *PM* can also be visualized through LR. In this system, two of the key parameters to describe the nanocylinder arrangement are orientational coherence and the extent of splay in the nanocylinder orientation. While freeze-fracture SEM limits our ability to quantify these parameters (for example, through methods such as an autocorrelation function for orientational coherence and divergence of nanocylinder orientation for extent of splay), the observation of broad peaks in ultrasmall X-ray scattering (USAXS) of these heterodimers provides evidence for coherence in the long-range order of nanocylinders (Figure S55). Beyond this measurement, we can articulate qualitative differences between spherulite types and relate them to the optical properties. The correlation between coherence of nanocylinder orientation and LR can be understood by considering each nanocylinder as a flexible rod. Thus, a stronger alignment of these “rods” results in a greater LR for a given collective rod orientation.

*MP*-I exhibits average LR of |0.039| radians (Figure 2E), mirroring the short coherence length for nanocylinder orientation revealed by cross-sectional SEM (Figure 4C). However, due to the size of the nanocylinders, locally coherent regions are large enough to obviate optical isotropy. In *MP*-II, the average LR is |0.30| radians, larger than the LR in *MP*-I. A higher degree of coherence in the nanocylinder orientation is observed in SEM (Figures 4E and S56). In *MP*-II, splay of the twisted nanocylinder bundles observed by SEM causes an oscillation in the LR and CR. These appear as concentric rings centered at each spherulite nucleus with an average pitch of 2.45 ± 0.07 μm (Figure 4D, F–G).

*PM*-I grows as coarse spherulites with distinct radial striations (Figure 2G). SEM of the surface of *PM*-I spherulites reveals that the nanocylinder directors form arcs as they reorient between alignment with the surface normal (pointing up) and within the surface plane (lying flat) (Figure 5A,B). POM of a given spherulite can be compared to a map of the nanocylinder orientations determined from SEM of the same region (Figure 5C,D). Within a single spherulite, the periodic reorientation of the nanocylinder director always occurs with the same handedness. In Figure 5D, in the spherulite on the left, the director splay adopts a clockwise direction (characterized by green/blue directors), whereas it is anticlockwise (characterized by red/yellow directors) in the adjacent spherulite across the domain boundary. As such, the orientation of nanocylinders in *PM*-I possess a sense, which can be visualized by mapping the nanocylinder orientation at the spherulite center (Figure 5E,F). The nanocylinder directors are on average offset from the radial vector originating at the spherulite center. However, periodic reorientation of nanocylinders occurs, resulting in a distribution of nanocylinder orientations centered at an average angle of 19° ± 41° anticlockwise (Figures S57–58A). *PM*-I forms a racemic system of these spherulites; similar analysis of another *PM*-I spherulite reveals an average clockwise angle between the nanocylinder orientation and the radial vector (Figure S59). Direct correlation between the optical characteristics and the nanocylinder orientation from freeze-fracture SEM is not viable. While an optical measurement captures the LR through the depth of the sample, freeze-fracture SEM only visualizes a top projection of the nanocylinders dependent on fracture planes (Figure S60). Neither technique can fully characterize the nanocylinder arrangement throughout the depth of the sample. Therefore, although the chiral nanocylinder arrangement observed in SEM may be indicative of a 3D chiral structure, we can only definitively characterize the sense observed at the surface of a spherulite. An analysis of the orientation of the LR optical azimuth with respect to the radial vector does not indicate a significant offset (4° ± 30° anticlockwise, Figure S61).

The effect of external geometric constraints on the twist and splay of nanocylinders is important for dictating the nanocylinder arrangement. In *MP*, the tendency for nanocylinders to form bundles with a possible twist arising from the curved nanocylinder shape, is demonstrated in the sample crystallized with a free surface (Figure 6A,B). Similar twisted bundles are also observed when *MP* is crystallized in sandwiched cells (Figure 4A). However, confinement impacts the orientation of the twisted bundles and the effect of secondary nucleation. With higher coherence of nanocylinder orientation, *MP*-II exhibits geometrically frustrated splay of nanocylinders in the form of periodic splay events of twisted nanocylinder bundles. Splay events are likely due to the twisted bundles approaching a threshold diameter at which the boundary conditions (imposed by the substrates) and confinement associated with the growing spherulite (imposed by adjacent bundles) prevent the bundle from growing to a larger diameter and induce periodic secondary nucleation. Beyond imposed boundary conditions, intrinsic properties of spontaneously twisted bundles, such as the interplay between surface energy and the elastic costs of twisted bundle formation, have been suggested by theoretical studies to lead to self-limiting growth mechanisms.^98^ These factors could also influence the bundle dimensions observed in this system.

**Figure 6:**
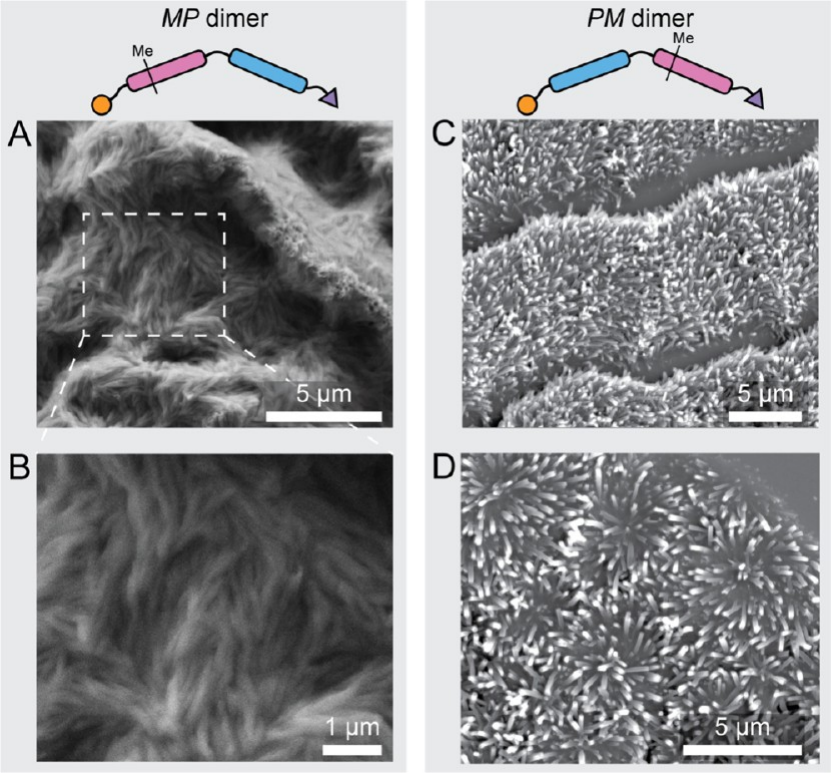
Scanning electron micrographs of heterodimers crystallized with a free surface. (A) Scanning electron micrograph of free surface *MP* demonstrating nanocylinder bundles. (B) Magnified region of **A**. (C,D) Scanning electron micrographs of free surface *PM* demonstrating (C) periodic nanocylinder splay and (D) small, highly splayed nanocylinder spherulites. All samples were cooled 2 °C min^–1^.

For *PM*, the splay of the nanocylinder director likely competes with the geometric constraints imposed by the substrates. When crystallized both in sandwiched cells and in open-surface conditions, splayed nanocylinder arrangements are observed. The formation of highly splayed structures in *PM* in the absence of the geometric constraints imposed by the top sample–glass interface indicates a driving force for splay of nanocylinders during crystallization, which may be due to their curved shape (Figure 6C,D). In sandwiched cells, the competition between this driving force for splay and geometric constraints may lead to regions of director reorientation separated by domain walls; this arrangement can be justified if the driving force for splay outweighs the energetic cost of producing domain walls. Periodically reorienting directors have been observed in mesogenic systems as a result of the competition between a twist or splay driving force and boundary conditions.^99^ Notably, optical banding arising from this competition has been observed in thin achiral smectic and hexatic systems^100,101^ and Langmuir monolayers of amphiphiles,^102^ in which splay is induced by polar (up–down) asymmetry from surface interactions. We note an important distinction in our system from these simple models; while many of these systems are dynamic and their director fields represent a local equilibrium state,^101^ the periodic reorientation of the nanocylinders here develops as part of a growing spherulite, and the nanocylinders are unable to rearrange after crystallization.

In a system with bands of director reorientation, either an achiral or chiral configuration can be adopted. If adjacent bands possess alternating reorientation directions, the system has an achiral configuration. If all bands exhibit the same reorientation direction, the system is chiral (Figure S58B). Theoretical calculations for smectic monolayers have suggested that the achiral configurations are preferred.^99,103^ However, in this system, the crystalline nanocylinders are unable to rearrange and relax into the most energetically favorable configuration during growth, likely resulting in kinetic trapping into a chiral configuration at the surface. Additionally, the radial growth of the spherulite necessitates that, for the nanocylinder reorientation to maintain a relatively constant splay and therefore domain thickness, new bands must be introduced during growth. Such a splay-driven defect patterns in the arrangement of nanocylinders can be observed as dislocations in the *B*_7_ phase^104^ and the “dense branching morphology” used to describe Langmuir monolayers.^105^ As such, a similar defect pattern may produce the dark chevron-like disclination lines observed in *PM*-I under crossed polarizers (Figure 2G).

Despite the kinetically trapped state of *PM*-II, SEM of the surface of a *PM*-II sample suggests the existence of a degree of long-range order with some similarities to that in *PM*-I (Figure S62). *PM*-II is formed at low temperatures (below the *T*_ni_) and consists of small spherulites that rapidly impinge upon each other. Some spherulites show evidence of a circular arrangement of nanocylinders consistent with a nanocylinder arrangement similar to the splay-driven orientation rearrangement observed in *PM*-I (Figure S62A). However, the spherulites of *PM*-II form a high defect density optical texture with −1/2 defects at the intersection between spherulites and do not exhibit observable optical banding (Figures 2H and S43).

## Conclusion

Understanding the origins of emergent chirality is important for exploiting and developing new materials toward controlled and biomimetic self-assembly, asymmetric synthesis, and chiroptical applications. In this study, we explore two structurally similar achiral mesogenic dimers that differ only in the position of a methyl group. Although both systems display supramolecular chirality (one through the bulk and one at the surface), the differences in their hierarchical assembly result in drastically different manifestations of chirality. Both dimers assemble to form nanocylinders with curvature as a function of methyl group placement. We propose that subtle differences in nanocylinder dimensions drive their distinct higher-order assemblies and associated chiroptical properties. Changes in the chemical structure and processing temperature also tune the relative rates of nucleation, growth, and secondary nucleation. We further propose that nanocylinders of both structures exhibit a tendency to splay, with optically banded structures arising from a balance of nanocylinder splay and geometric confinement.

The characteristics of these nanocylinder arrangements bear resemblance to colloidal liquid crystals, including their alignment, defects, deformations (i.e., bend, twist, and splay), and emergent chirality in their hierarchical assembly. While the well-established language of liquid crystals can be helpful for describing these nanocylinder arrangements, this system cannot be solely understood by the same principles. Here, nanocylinder growth and assembly occur simultaneously and cooperatively in a crowded environment, influenced by the dynamics of small-molecule diffusion and secondary nucleation in the growing crystalline structure. These results invite the development of theory and simulations that can describe the forces guiding the assembly of nanocylinders into hierarchical structures while accounting for the kinetic effects that are key to this nonequilibrium crystallization process. We anticipate that such theories may be of interest in other self-assembled systems of tubular subunits and other crystalline structures with significant curvature that are highly sensitive to precise molecular structure,^62,63^ such as in dense biological systems.

This work highlights complex nonequilibrium processes driven by molecular structure, microstructural curvature, and crystallization conditions. Through subtle changes to these driving factors, we demonstrate control over supramolecular order in these unique systems composed of hierarchically arranged nanocylinders. Ultimately, we emerge with new insights into how small changes in molecular design, tuned by sequence and extendable to a variety of mesogens and oligomer lengths, can drive dramatic effects in molecular self-assembly.

## Experimental Section

### Synthesis and Materials

The heterodimers were synthesized through copper-catalyzed azide–alkyne coupling (CuAAC) of a methylated and nonmethylated mesogenic monomer. The protocol for *P*_1_, *P*_2_, and precursor fragments for *M*_1_ followed a previously reported strategy.^82^ Successful synthesis was confirmed through NMR. Detailed synthetic information is provided in Section S1, and chemical characterization is provided in Section S2. All chemicals were purchased commercially and used without additional purification.

### Nuclear Magnetic Resonance (NMR)

^1^H nuclear magnetic resonance (NMR) was performed using a 500 MHz NMR spectrometer (Bruker). ^13^C NMR was performed at 126 MHz. Samples were dissolved in CDCl_3_, and the solvent peaks (CDCl_3_ for ^13^C NMR, residual CHCl_3_ for ^1^H NMR) were used as the reference.

### Gel Permeation Chromatography

Gel permeation chromatography (GPC) was measured using tetrahydrofuran (with 2 vol % ethanol as a stabilizer) as the eluent at a 1 mL min^–1^ flow rate on a Shimadzu GPC system equipped with two columns (PSS 10000 Å SDV) in series and a refractive index detector (RID-20A). Monodisperse polystyrene (PS) standards were used to determine the PS-equivalent molecular weight of the dimers from the elution times.

### High-Resolution Mass Spectrometry (HRMS)

High-resolution mass spectrometry (HRMS) was performed on a liquid chromatography/mass spectrometry system (Agilent 6230 Time-of-Flight LC/MS 1260LC) with electrospray ionization (ESI) in positive ionization mode. The compounds were dissolved in a 1:1 mixture of acetonitrile and water with 0.1% formic acid. HRMS analysis was limited to compounds soluble in this chromatography solvent.

### Quantum Chemistry Calculations and Conformational Analysis

Conformational ensembles of alkyl and triazole-linked phenyl benzoate dimers were generated using 1D rotational-isomeric-state (RIS) sampling from dihedral scans of representative rotor fragments. Each molecule was classified as one of eight possible rotors, which were scanned in Psi4 ^106^ on a 15° grid using torsiondrive^107^ via QCEngine.^108^ Scans were performed at the ωB97XD/6-31G­(d,p) level of theory, which has previously applied to other LC systems including the *P*_2_ dimer.^82,109,110^ Boltzmann weighting at 10 K was then used to generate probability distributions at the energy minima most likely to be populated in a crystal. For each spacer length, 500 conformers were embedded using the ETKDGv3 method implanted in RDKit,^111^ drawing each rotor from its Boltzmann distribution. The resulting structures were relaxed first with UFF^112^ and then with the UMA-small 1.2 machine-learned interatomic potential^113^ to give the final bend angles and energies. Mean bend angles from these ensembles are reported in Figures S27 and S28, and further computational details are given in Section S3.

### Differential Scanning Calorimetry (DSC)

Differential scanning calorimetry (DSC) was performed using a TA Discovery DSC 2500. Samples were hermetically sealed in aluminum DSC pans (TA Tzero), with a small puncture hole to facilitate nitrogen flow during heating/cooling cycles to minimize thermal degradation. Heating/cooling cycles were performed at a rate of 2 °C min^–1^. Enthalpies and peak temperatures were calculated within the TRIOS software.

### Preparation of Glass Sandwich Cells

Samples were sandwiched between two glass substrates for polarized optical microscopy. Cells of both controlled thickness and uncontrolled thickness were prepared. Thickness was controlled by two methods. To prepare cells with ∼5 μm thickness, silica beads obtained from a water suspension (Sigma-Aldrich, 5.16 ± 0.15 μm diameter determined by the supplier using a Beckman Coulter Multisizer III) was used as a spacer, and the sample was melted and wicked into the glass sandwich by capillary action, as described previously.^82^ To prepare cells with uncontrolled thickness, samples were melted on a glass substrate, and another substrate was placed on top to gently compress the sample melt. Glass coverslips and indium tin oxide-coated glass were used as substrates for uncontrolled thickness cells, while only glass coverslips were used for controlled thickness cells. Thermal processing was performed using one of two controlled heating/cooling stages: a Linkam FTIRSP600 equipped with Linkam T95PE and active cooling with liquid nitrogen (Linkam LNP95) or an Instec HCS421VXY equipped with Instec mK2000B and active cooling with liquid nitrogen (Instec LN2-P). A nitrogen atmosphere was maintained in the heating chamber to reduce thermal degradation.

### Polarized Optical Microscopy (POM)

POM was performed in transmission under a bright field configuration. Two Zeiss Axioscope upright optical microscopes were used. One setup (Zeiss Axioscope A1) was equipped with 5× (EC Epiplan 5×/0.13 HD), 10× (EC Epiplan Neofluar 10×/0.25 HD DIC), 50× (EC Epiplan 50×/0.75), and a long-working-distance 50× (LD Epiplan-Neofluar 50×/0.55 DIC) objective. The other system (Zeiss Axioscope A5) was equipped with 5× (EC Epiplan-Neofluar 5×/0.13 DIC), 10× (EC Epiplan-Neofluar 10×/0.25 DIC), long working distance 20× (LD EC Epiplan-Neofluar 20×/0.22 DIC), and 50× (LD EC Epiplan-Neofluar 50×/0.55 DIC). In the Zeiss Axioscope A1 system, the analyzer orientation is fixed, while the polarizer can be rotated. Both the analyzer and the polarizer can be rotated independently in the Zeiss Axioscope A5 system. In situ heating/cooling was performed using a controlled heating stage (Linkam FTIRSP600 equipped with Linkam T95PE) and active cooling with liquid nitrogen (Linkam LNP95) positioned on the sample stage. A nitrogen atmosphere was maintained in the heating chamber to reduce thermal degradation.

### Mueller Matrix Microscopy

Mueller matrix imaging (MMI) was achieved using a microscope with a polarization-sensitive camera.^114^ The camera (Flir Blackfly BFS–U3–51S5P–C with a Sony IMX250MZR polarization sensor) contains an on-sensor polarization array (0°, 45°, 90° and 135° per superpixel) that, when combined with a discretely rotating compensator (QWP polymer retarder film, Edmund Optics, WP140HE), measures three of the four Stokes vectors (*S*_0_, *S*_1_, and *S*_2_), allowing complete characterization of nonpolarizing films. The pixel-wise intensities projected onto the camera are inverted to reveal the first three rows of the Mueller matrix. Recovery of the fourth row is determined analytically from the 12 elements.^115^ Micrographs were recorded at 617 nm. Only samples with uncontrolled thickness (<5 μm) were used for MMI to maximize feature resolution. Due to the unquantified thickness, we report linear and circular retardance rather than birefringence.

### Ultrasmall-, Small-, and Wide-Angle X-ray Scattering (USAXS/SAXS/WAXS)

“Bulk” X-ray scattering samples were prepared between two polyimide sheets (Kapton, 8 μm thick) with a brass washer (M3, 0.5 mm thick) as a spacer. In situ heating and cooling were performed using a Linkam FTIRSP600 heating stage with a Linkam T95PE controller and liquid nitrogen cooling. The effective heating and cooling rate of in situ measurements was 2 °C min^–1^. Ex situ samples were prepared using the controlled heating stage previously described. “Thin” X-ray scattering samples were prepared by melting and sandwiching them between two polyimide sheets without a spacer.

SAXS and WAXS were performed using a Xenocs Xeuss 3.0 with a high-intensity Cu K_α_ X-ray source and a 2D detector (Dectris Eiger 2R 1M-pixel 2D). The beam size was 0.3 mm × 0.3 mm. The sample-to-detector (STD) distance was set by a calibration relative to the (100) reflection of a lanthanum hexaboride (LaB6) standard (*d*-spacing = 4.157 Å). To further ensure accurate *q*-values, the STD distance was calibrated postmeasurement relative to the lowest *q* peak of the polyimide substrate (*d*-spacing = 15.62 Å) as described previously.^82^ For in situ heating and cooling measurements, the calibration of all spectra in a series was determined using the spectrum collected at the highest temperature. The SAXS and WAXS data are presented as a single spectrum, resulting in differing noise levels at the cutoff between the data collected at the two detector positions. USAXS was performed using a 9.65 keV X-ray beam at the 11-ID CHX beamline of Brookhaven National Laboratory.

### Scanning Electron Microscopy (SEM)

SEM samples were prepared by freeze-fracturing glass coverslip sandwiches of uncontrolled thickness after controlled cooling or isothermal crystallization using the controlled heating stage described in the section on polarized optical microscopy. The samples were then affixed to a stub using conductive carbon tape and coated with ∼4 nm iridium using a sputter coater (Leica EM ACE600 Sputter Coater). Unless stated otherwise, SEM was performed using a Quanta 200 FEG Environmental-SEM. For some images, SEM was performed using a Verios 460 Extreme High Resolution SEM.

### Image Analysis

Nanocylinder inner and outer diameters were determined from SEM images using ImageJ software. For each nanocylinder, the sizes of the horizontal cross section and hollow region were estimated by manually mapping the contour using the polygonal selection tool followed by fitting the selection to an ellipse. To account for tilt in the nanocylinder orientation and a nonhorizontal cross section cut of the nanocylinder face, the major axis of the fitted ellipse was taken as the diameter. A sample size of >30 nanocylinders was used for each diameter calculation.

Nanocylinder orientations were manually mapped using ImageJ software and MATLAB scripts. Each nanocylinder was represented by an end-to-end vector approximated by the closest and farthest points of the nanocylinder (from the perspective of the viewer) using the Point tool in ImageJ. The end points were identified by the brightness gradient within each nanocylinder, where the brightest point indicates the accumulation of charges at the edge of a nanocylinder. A MATLAB script was used to calculate the angle formed by each vector with respect to the horizontal and overlay these vectors onto the original SEM image, where the nanocylinder orientation is represented by a color heatmap.

Optical banding pitch was determined by calculating and averaging the distance between peaks and troughs in the line intensity of LR and CR.

## Supplementary Material



## Data Availability

All data relating to this publication are openly available from Princeton DataSpace (DOI: 10.34770/j3cz-3j92).

## Notes

Synthetic schemes and methods, chemical characterization, additional details for quantum chemistry calculations and conformational analysis, and additional results including differential scanning calorimetry, polarized optical micrographs, Mueller matrix images, scanning electron micrographs, and X-ray scattering (PDF)

## §

C.L.C.C: Stratingh Institute for Chemistry, University of Groningen, 9700 AB Groningen, The Netherlands
