# Supplementary material for: Hierarchical Chiral Self-Assembly of Nanocylinders Composed of Sequence-Defined Mesogenic Dimers
Source: J Am Chem Soc. 2026 Jun 17;148(25):26192–208. doi: 10.1021/jacs.6c05456 (PMC13339157; doi:10.1021/jacs.6c05456)
Supplement: Supplementary file 1 [file ja6c05456_si_001.pdf]

Hierarchical chiral self-assembly of nanocylinders composed of sequence-defined mesogenic dimers

Supplementary Information

Emily C. Ostermann[1⊥], Chun Lam Clement Chan[1⊥#], Justin Bendesky[2], Jacob S. Votava[1], Eva S. M. Reed[1], Shawn M. Maguire[1], Michael A. Webb[1], Stephanie S. Lee[2], Bart Kahr[2], Emily C. Davidson[1*]

⊥Denotes equal contribution

[1]Department of Chemical and Biological Engineering, Princeton University, Princeton, NJ, 08540

[2]Department of Chemistry and Molecular Design Institute, New York University, New York, NY, 10003

[*]*Corresponding Author* Email: edavidson@princeton.edu (E.C.D.)

Table of Contents

Section S1: Synthetic schemes and methods

Section S2: Chemical characterization

Section S3: Additional details for quantum chemistry calculations and conformational analysis

Section S4: Supplementary tables and figures

Table S1

Figures S1-S62

References

Section S1: Synthetic schemes and methods

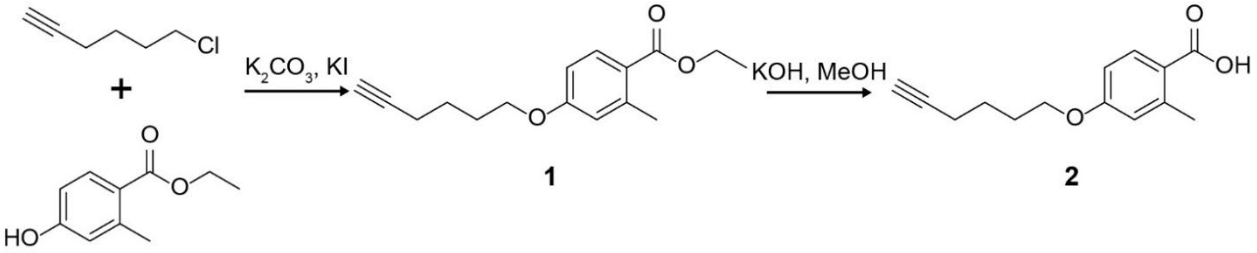

Scheme S1: Chemical scheme to form a fragment towards the synthesis of *M*₁

Ethyl 4-(hex-5-yn-1-yloxy)-2-methylbenzoate **1**: To a solution of ethyl 4-hydroxy-2-methylbenzoate (10 g, 55 mmol, 1 eq) in acetonitrile (250 mL) was added K_2CO_3 (20.7 g, 150 mmol, 2.7 eq) and KI (12.0 g, 72 mmol, 1.3 eq). 6-chloro-1-hexyne (8.7 mL, 8.4 g, ρ = 0.96 g mL^{-1}, 72 mmol, 1.3 eq) was subsequently added, and the mixture was stirred under gentle reflux at 90 ºC for 3 days. The mixture was quenched with water (200 mL), and the product was extracted from the aqueous layer using EtOAc (200 mL, 2 × 100 mL). The organic fractions were combined, washed with brine (200 mL), dried over $MgSO_4$ and concentrated *in vacuo*. The crude product was subsequently purified using flash column chromatography (Hex, then 1:4 EtOAc:Hex) and dried *in vacuo* to yield **1** as a yellow liquid (15.7 g). At this stage, some 6-chloro-1-hexyne starting material (~ 16 mol% of product) remained in the product, resulting in an apparent yield of 60 mmol and 109 %. This starting material does not interfere with the subsequent hydrolysis step and is removed during the purification of that step. *Anal.*: R_f = 0.65 (1:4 EtOAc:Hex); ^{1}H NMR (500 MHz, $CDCl_3$) δ / ppm, 7.95 – 7.89 (m, 1H), 6.75 – 6.69 (m, 2H), 4.32 (q, J = 7.2 Hz, 2H), 4.02 (t, J = 6.3 Hz, 2H), 2.59 (s, 3H), 2.28 (td, J = 7.1, 2.7 Hz, 2H), 2.00 – 1.93 (m, 1H), 1.91 (dt, J = 8.2, 6.5 Hz, 2H), 1.77 – 1.68 (m, 2H), 1.37 (t, J = 7.1 Hz, 3H); ^{13}C NMR (126 MHz, $CDCl_3$) δ / ppm, 167.32, 161.77, 143.08, 133.03, 122.13, 117.53, 111.46, 84.12, 68.87, 67.42, 60.46, 28.29, 25.11, 22.48, 18.28, 14.54; HRMS-ESI, Calcd. for $C_{16}H_{20}O$: *m*/*z* = 261.1485 [**3** + H]$^+$; Found: *m*/*z* = 261.1484 [**3** + H]$^+$.

4-(Hex-5-yn-1-yloxy)-2-methylbenzoic acid **2**: **1** (15.4 g, 59 mmol, 1 eq) was dissolved in methanol (150 mL) and potassium hydroxide (26.6 g, 474 mmol, 8 eq) was added. The mixture was stirred at 35 ºC overnight. The mixture was quenched with water (400 mL) and acidified *via* portion-wise addition of concentrated hydrochloric acid (HCl) until pH < 3. The suspension was then extracted with DCM (200 mL, 2 × 100 mL). The organic fractions were collected, washed with brine (300 mL), dried over $MgSO_4$, and concentrated *in vacuo*. The crude solid was then suspended in hexanes, collected through vacuum filtration, washed on the filter with hexanes, and dried *in vacuo* to yield **2** as a pale yellow solid (10.6 g, 46 mmol, 77 %). *Anal.*: R_f = 0.52 (3:2 EtOAc:Hex); ^{1}H NMR (500 MHz, $CDCl_3$) δ / ppm, 8.08 – 8.02 (m, 1H), 6.76 (dd, J = 6.4, 2.7 Hz, 2H), 4.04 (t, J = 6.2 Hz, 2H), 2.64 (s, 3H), 2.29 (td, J = 7.0, 2.6 Hz, 2H), 2.00 – 1.89 (m, 3H), 1.78 – 1.68 (m, 2H); ^{13}C NMR (126 MHz, $CDCl_3$) δ / ppm, 171.54, 162.64, 144.39, 134.20, 120.39, 117.79, 111.60, 84.09, 68.90, 67.51, 28.26, 25.10, 22.78, 18.28.

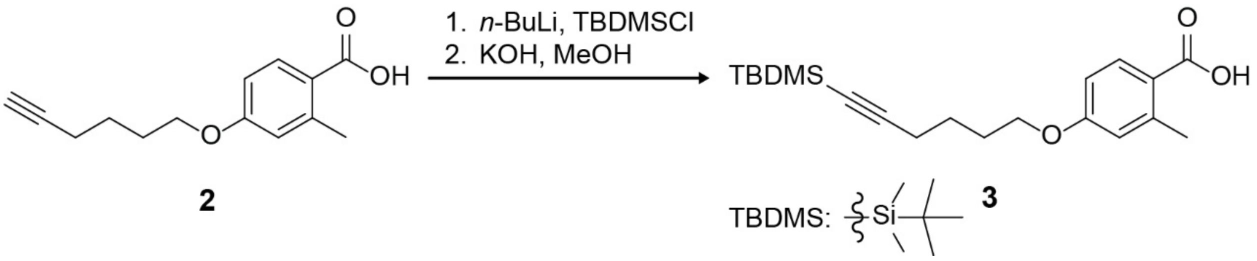

Scheme S2: Chemical scheme to form a fragment towards the synthesis of TBDMS-protected *M*₁

4-((6-(Tert-butyldimethylsilyl)hex-5-yn-1-yl)oxy)-2-methylbenzoic acid **3**: **2** (5.0 g, 21.5 mmol, 1 eq) was dissolved in dry THF (200 mL) under a nitrogen environment and cooled to -78 ºC in a mixture of dry ice and acetone. *n*-butyl lithium (*n*-BuLi, 2.5 mol dm^{-3} in hexane, 21 mL, 52 mmol, 2.4 eq) was added dropwise *via* a pressure-equalized dropping funnel. The dropping funnel was washed with an additional 50 mL dry THF to remove any remaining *n*-BuLi, and the mixture was stirred at -78 ºC for 30 mins before a solution of *tert*-butyldimethylsilyl chloride (TBDMSCl, 9.7 g, 65 mmol, 3 eq) in dry THF (20 mL) was added dropwise. The mixture was then allowed to return to room temperature and stirred for an additional 5 hrs. Subsequently, the reaction mixture was quenched with cold brine (200 mL). The organic products were extracted from the mixture using EtOAc (3 × 100 mL) and concentrated to yield a mixture containing the intermediate tert-*butyldimethylsilyl 4-((6-(*tert-*butyldimethylsilyl)hex-5-yn-1-yl)oxy)-2-methylbenzoate*, which was used without further purification. The intermediate mixture was subsequently redissolved in methanol (50 mL). Potassium hydroxide (9.7 g, 173 mmol, 8.0 eq rel. to **2**) was added, and the reaction was stirred for 1 hr at 35 ºC. The mixture was diluted with water (200 mL) and acidified *via* portion-wise addition of concentrated hydrochloric acid (HCl) until pH < 3. The organic fractions were then extracted from the mixture with DCM (3 × 100 mL), washed with brine (100 mL), dried over $MgSO_4$, and concentrated *in vacuo*. The crude product was purified by flash chromatography (1:4 EtOAc:Hex, then 1:1 EtOAc:Hex) and dried in a vacuum oven (at 50 °C) to yield **3** as a yellow solid (3.5 g, 10.1 mmol, 47 %). *Anal.*: R_f = 0.64 (1:1 EtOAc:Hex); ^1H NMR (500 MHz, $CDCl_3$) δ / ppm, 10.57 (s, 1H), 8.07 – 8.01 (m, 1H), 6.76 (dd, *J* = 6.2, 2.7 Hz, 2H), 4.04 (t, *J* = 6.3 Hz, 2H), 2.63 (s, 2H), 2.33 (t, *J* = 7.0 Hz, 2H), 1.97 – 1.88 (m, 2H), 1.76 – 1.67 (m, 2H), 0.93 (s, 1H*), 0.93 (s, 7H*), 0.08 (s, 5H*); ^{13}C NMR (126 MHz, $CDCl_3$) δ / ppm, 171.00, 162.66, 144.37, 134.17, 120.26, 117.80, 111.61, 107.42, 83.26, 77.41, 77.36, 77.16, 76.91, 67.57, 28.35, 26.24, 25.36, 22.76, 19.71, 16.67, -4.29.

It should be noted that the increased integral (labelled for Fragment **3** with a *) rel. to the predicted no. of protons (respectively 8H and 5H) at 0.93 and 0.08 ppm, which correspond to the methyl protons on the TBDMS protecting group, is likely due to additional TBDMS groups arising from the starting material or cleaved during the hydrolysis step. The large added excess of TBDMSCl starting material was removed *via* sublimation *in vacuo*, and remaining TBDMSCl is removed in the coupling step to produce ***M*₁**.

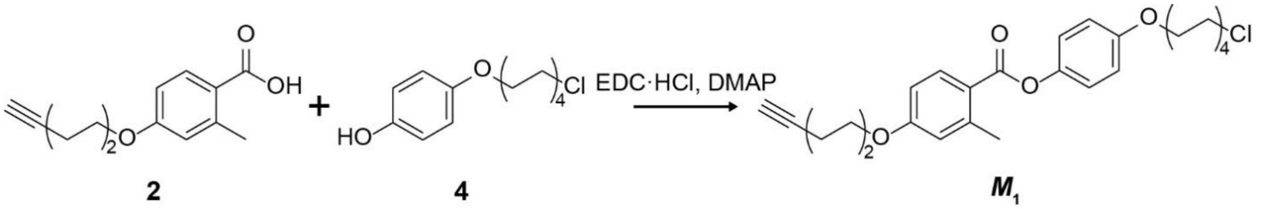

Scheme S3: Chemical scheme towards the coupling reaction to prepare *M*1

4-((8-chlorooctyl)oxy)phenyl 4-(hex-5-yn-1-yloxy)-2-methylbenzoate ***M*1**: To a solution of **2** (3.2 g, 14 mmol, 1 eq) in DCM (250 mL) was added *4-((8-chlorooctyl)oxy)phenol* **4** (3.0 g, 14 mmol, 1 eq) for which the synthesis has been previously described,[1] and the mixture was cooled to 0 °C in an ice bath. N-(3-Dimethylaminopropyl)-N′-ethylcarbodiimide hydrochloride (EDC·HCl, 3.2 g, 16 mmol, 1.2 eq) and 4-(dimethylamino)pyridine (DMAP, 170 mg, 1.4 mmol, 0.1 eq) were added. The mixture was subsequently allowed to warm to room temperature slowly (by removing the ice bath) and stirred overnight. The crude product was concentrated *in vacuo* and then redissolved in EtOAc (200 mL) and water (200 mL). The organic layer was separated from the aqueous layer, and the aqueous layer was further extracted with EtOAc (2 × 100 mL). The combined organic layers were washed with brine (200 mL), dried over $MgSO_4$, and concentrated *in vacuo*. The crude product was purified via flash chromatography (1:4 EtOAc:Hex) and dried *in vacuo* to yield ***M*1** as a pale orange solid (4.7 g, 13 mmol, 94 %). *Anal.*: R_f = 0.35 (1:4 EtOAc:Hex); ^{1}H NMR (500 MHz, $CDCl_3$) δ / ppm, 8.18 – 8.12 (m, 1H), 7.08 (d, *J* = 8.8 Hz, 2H), 6.92 (d, *J* = 8.8 Hz, 2H), 6.80 (t, *J* = 3.5 Hz, 1H), 6.79 (s, 1H), 4.06 (t, *J* = 6.3 Hz, 2H), 3.95 (t, *J* = 6.5 Hz, 2H), 3.54 (t, *J* = 6.8 Hz, 2H), 2.65 (s, 3H), 2.30 (td, *J* = 7.0, 2.7 Hz, 2H), 2.01 – 1.89 (m, 3H), 1.80 (d, *J* = 6.8 Hz, 1H), 1.80 – 1.69 (m, 5H), 1.52 – 1.31 (m, 9H); ^{13}C NMR (126 MHz, $CDCl_3$) δ / ppm, 165.88, 162.42, 156.83, 144.48, 144.39, 133.71, 122.76, 120.76, 117.78, 115.23, 111.67, 84.09, 68.91, 68.48, 67.53, 45.30, 32.75, 29.36, 29.35, 28.96, 28.26, 26.96, 26.09, 25.09, 22.67, 18.28.

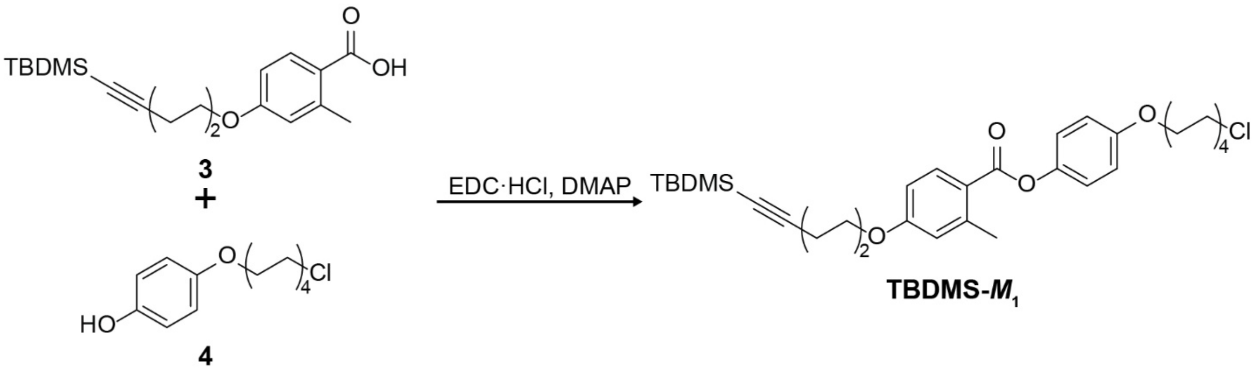

Scheme S4: Chemical scheme towards the coupling reaction to prepare TBDMS-protected *M*1

4-((8-chlorooctyl)oxy)phenyl 4-((6-(tert-butyldimethylsilyl)hex-5-yn-1-yl)oxy)-2-methylbenzoate **TBDMS-*M*1**: To a solution of **3** (3.2 g, 9.2 mmol, 1 eq) in DCM (200 mL) was added **4** (2.0 g, 7.8 mmol, 0.8 eq), and the mixture was cooled to 0 °C in an ice bath. N-(3-Dimethylaminopropyl)-N′-ethylcarbodiimide hydrochloride (EDC·HCl, 2.1 g, 11 mmol, 1.2 eq) and 4-(dimethylamino)pyridine (DMAP, 110 mg, 0.9 mmol, 0.1 eq) was added. The mixture was subsequently allowed to warm to room temperature slowly (by removing the ice bath) and stirred overnight. The crude product was concentrated *in vacuo*, then redissolved in EtOAc (200 mL) and water (200 mL). The organic layer was separated from the aqueous layer, and the organic layer was further extracted with EtOAc (2 × 100 mL). The combined organic layers were washed with brine (200 mL), dried over $MgSO_4$, and concentrated *in vacuo*. The crude product was purified *via* flash chromatography (1:6 EtOAc:Hex), followed by a second purification step *via* flash chromatography (1:7 EtOAc:Hex), and dried *in vacuo* to yield **TBDMS-*M*1** as a viscous yellow liquid (3.4 g, 5.8 mmol, 63 %). *Anal.*: R_f = 0.44 (1:7 EtOAc:Hex); ^{1}H NMR (500 MHz, $CDCl_3$) δ / ppm, 8.18 – 8.13 (m, 0H), 7.12 – 7.05 (m, 1H), 6.96 – 6.89 (m, 1H), 6.83 – 6.77 (m, 1H), 4.06 (t, *J* = 6.2 Hz, 1H), 3.96 (t, *J* = 6.5 Hz, 1H), 3.54 (t, *J* = 6.7 Hz, 1H), 2.65 (s, 1H), 2.34 (t, *J* = 7.0 Hz, 1H), 1.94 (dq, *J* = 8.5, 6.4 Hz, 1H), 1.83 – 1.74 (m, 2H), 1.77 – 1.69 (m, 1H), 1.52 – 1.42 (m, 2H), 1.44 – 1.31 (m, 2H), 0.94 (s, 4H), 0.09 (s, 2H); ^{13}C NMR (126 MHz, $CDCl_3$) δ / ppm, 165.84, 162.43, 156.82, 144.48, 144.34, 133.68, 122.74, 120.72, 117.79, 115.21, 111.67, 107.40, 83.23, 68.46, 67.56, 45.25, 32.73, 29.35, 29.33, 28.94, 28.33, 26.94, 26.22, 26.07, 25.35, 22.64, 19.69, 16.64, -4.30.

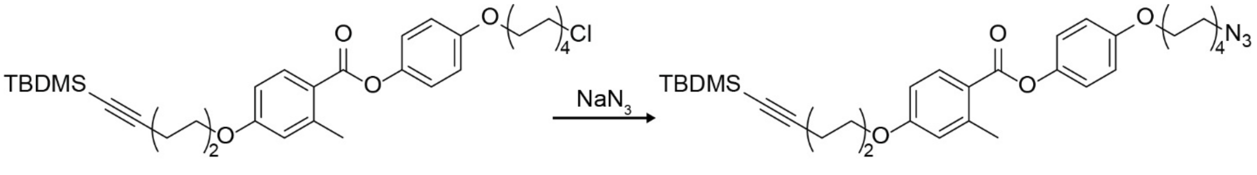

Scheme S5: Chemical scheme to prepare TBDMS-protected M_1 with an azide moiety

4-((8-azidooctyl)oxy)phenyl 4-((6-(tert-butyldimethylsilyl)hex-5-yn-1-yl)oxy)-2-methylbenzoate **TBDMS-*M*$_1$-N$_3$**: **TBDMS-*M*$_1$** (140 mg, 0.24 mmol, 1 eq) was dissolved in DMF (4 mL), and sodium azide (24 mg, 0.36 mmol, 1.5 eq) was added to the solution before the mixture was stirred at 60 °C overnight. The solution was quenched with 10 mL water, and the product was extracted using EtOAc (10 mL, 2 × 5 mL). The combined organic fractions were washed with brine (10 mL), dried over Na_2SO_4. The crude product was then concentrated *in vacuo* to yield **TBDMS-*M*$_1$-N$_3$** as a viscous amber liquid (120 mg, 0.21 mmol, 88 %). *Anal.*: R_f = 0.38 (1:8 EtOAc:Hex); ^{1}H NMR (500 MHz, $CDCl_3$) δ / ppm, 8.15 (d, *J* = 9.5 Hz, 0H), 7.12 – 7.05 (m, 1H), 6.95 – 6.88 (m, 1H), 6.79 (s, 1H), 4.06 (t, *J* = 6.3 Hz, 1H), 3.96 (t, *J* = 6.5 Hz, 1H), 3.27 (t, *J* = 7.0 Hz, 1H), 2.65 (s, 1H), 2.33 (t, *J* = 7.0 Hz, 1H), 1.93 (dq, *J* = 7.9, 6.4 Hz, 1H), 1.83 – 1.68 (m, 2H), 1.66 – 1.57 (m, 1H), 1.47 (dd, *J* = 11.0, 4.7 Hz, 1H), 1.46 – 1.33 (m, 1H), 1.37 (s, 1H), 0.93 (s, 3H), 0.09 (s, 2H); ^{13}C NMR (126 MHz, $CDCl_3$) δ / ppm, 165.89, 162.47, 156.85, 144.53, 144.38, 133.71, 122.77, 120.77, 117.82, 115.26, 111.71, 107.42, 83.27, 68.51, 67.61, 51.63, 29.38, 29.37, 29.24, 28.98, 28.36, 26.81, 26.24, 26.10, 25.38, 22.66, 19.72, 16.67, -4.28.

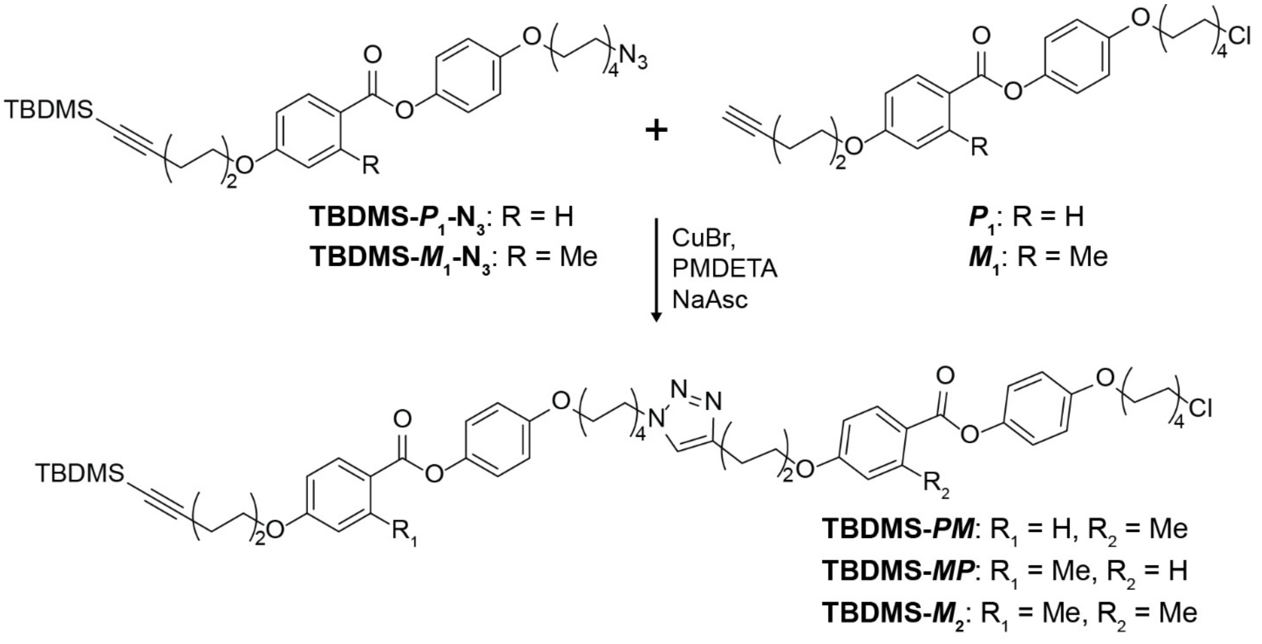

Scheme S6: Chemical scheme to prepare TBDMS-protected *PM*, *MP*, and *M*$_2$

Experimental note: To prevention oxidation, copper (I) bromide, which was supplied as an ampoule, was stored within a glove box, from which an aliquot taken prior to each CuAAC reaction.

TBDMS-*PM*: TBDMS-*P*$_1$-N$_3$ (1.6 g, 2.7 mmol, 1 eq) for which the synthesis is previously described[1] and ***M*$_1$** (1.3 g, 2.7 mmol, 1 eq) were dissolved in DMF (50 mL) in an oven-dried Schlenk flask. CuBr (20 mg, 0.14 mmol, 0.05 eq) and NaAsc (55 mg, 0.28 mmol, 0.1 eq) were subsequently added. PMDETA (58 µL, 48 mg, $\rho = 0.83$ g mL^{-1}, 0.28 mmol, 0.1 eq) was added and the solution was mixed quickly by shaking before being rapidly frozen in liquid nitrogen. The solution was then degassed by three cycles of freeze-pump-thaw. After degassing, the reaction was placed in an oil bath preheated at 50 ºC and allowed to stir for 2 hr before the reaction was exposed to air and concentrated *in vacuo*. The crude product was purified *via* flash chromatography (4:1 EtOAc:Hex, then 2:3 EtOAc:Hex) and dried *in vacuo* to yield **TBDMS-*PM*** as a pale yellow solid (2.54 g, 2.42 mmol, 90%). *Anal.*: $R_f = 0.53$ (3:2 EtOAc:Hex); ^{1}H NMR (500 MHz, $CDCl_3$) δ / ppm, 8.17 – 8.10 (m, 1H), 7.29 (s, 0H), 7.13 – 7.04 (m, 2H), 6.99 – 6.92 (m, 1H), 6.95 – 6.87 (m, 2H), 6.81 – 6.74 (m, 1H), 4.31 (t, J = 7.2 Hz, 1H), 4.11 – 4.03 (m, 2H), 3.95 (td, J = 6.5, 4.0 Hz, 2H), 3.54 (t, J = 6.7 Hz, 1H), 2.81 (q, J = 4.3 Hz, 1H), 2.64 (s, 1H), 2.34 (t, J = 6.9 Hz, 1H), 1.99 – 1.89 (m, 1H), 1.92 – 1.84 (m, 1H), 1.83 – 1.76 (m, 2H), 1.76 (d, J = 6.7 Hz, 1H), 1.76 – 1.69 (m, 1H), 1.55 (s, 1H), 1.47 (dtd, J = 14.1, 6.8, 3.4 Hz, 3H), 1.36 (pd, J = 5.2, 2.9 Hz, 5H), 0.93 (s, 4H), 0.09 (s, 3H); ^{13}C NMR (126 MHz, $CDCl_3$) δ / ppm, 165.87, 165.46, 163.45, 162.46, 156.87, 147.87, 144.58, 144.52, 144.38, 133.71, 132.37, 122.76, 122.64, 121.96, 120.77, 120.62, 117.79, 115.25, 115.22, 114.40, 111.70, 107.38, 83.31, 68.51, 68.42, 67.82, 67.79, 50.34, 45.29, 32.76, 30.48, 29.38, 29.35, 29.26, 29.08, 28.97, 28.81, 28.33, 26.97, 26.61, 26.24, 26.11, 26.10, 26.07, 25.51, 25.36, 22.66, 19.72, 16.67, -4.28.

TBDMS-*MP*: TBDMS-*M*$_1$-N$_3$ (1.7 g, 2.8 mmol, 1 eq) and ***P*$_1$** (1.2 g, 2.8 mmol, 1 eq) for which the synthesis is previously described[1] were dissolved in DMF (50 mL) in an oven-dried Schlenk

flask. CuBr (20 mg, 0.14 mmol, 0.05 eq) and NaAsc (56 mg, 0.28 mmol, 0.1 eq) were subsequently added. PMDETA (58 µL, 49 mg, ρ = 0.83 g mL^{-1}, 0.28 mmol, 0.1 eq) was added and the solution was mixed quickly by shaking before being rapidly frozen in liquid nitrogen. The solution was then degassed by three cycles of freeze-pump-thaw. After degassing, the reaction was placed in an oil bath preheated at 50 ºC and allowed to stir for 2 hr before the reaction was exposed to air and concentrated *in vacuo*. The crude product was purified *via* flash chromatography (1:4 EtOAc:Hex, then 3:2 EtOAc:Hex) and dried *in vacuo* to yield **TBDMS-*MP*** as a pale yellow solid (2.36 g, 2.25 mmol, 80%). *Anal.:* R_f = 0.57 (3:2 EtOAc:Hex); ^{1}H NMR (500 MHz, $CDCl_3$) δ / ppm, 8.13 (dd, J = 11.2, 9.1 Hz, 1H), 7.27 (d, J = 13.8 Hz, 1H), 7.13 – 7.04 (m, 2H), 6.95 (d, J = 8.7 Hz, 1H), 6.93 – 6.87 (m, 2H), 6.82 – 6.75 (m, 1H), 4.31 (t, J = 7.2 Hz, 1H), 4.06 (q, J = 6.0 Hz, 2H), 3.95 (q, J = 6.2 Hz, 2H), 3.54 (t, J = 6.7 Hz, 1H), 2.81 (s, 1H), 2.64 (s, 1H), 2.33 (t, J = 6.9 Hz, 1H), 1.98 – 1.89 (m, 2H), 1.92 – 1.87 (m, 2H), 1.83 – 1.73 (m, 3H), 1.76 – 1.68 (m, 1H), 1.52 – 1.41 (m, 3H), 1.41 – 1.31 (m, 5H), 0.93 (s, 1H), 0.93 (s, 4H), 0.09 (s, 1H), 0.09 (s, 2H); ^{13}C NMR (126 MHz, $CDCl_3$) δ / ppm, 165.89, 165.45, 163.43, 162.47, 156.89, 156.81, 147.83, 144.54, 144.52, 144.38, 133.71, 132.37, 122.78, 122.62, 121.96, 120.71, 120.62, 117.81, 115.23, 115.21, 114.37, 111.70, 107.42, 83.26, 77.41, 77.16, 76.91, 68.47, 68.43, 67.99, 67.60, 50.33, 45.29, 32.76, 30.48, 29.38, 29.35, 29.33, 29.25, 29.08, 28.96, 28.79, 28.35, 26.96, 26.60, 26.24, 26.11, 26.10, 26.06, 25.51, 25.37, 22.66, 19.71, 16.66, -4.29.

TBDMS-*M*$_2$: **TBDMS-*M*$_1$-N_3** (1.6 g, 2.7 mmol, 1 eq) and ***M*$_1$** (1.2, 2.7 mmol, 1 eq) were dissolved in DMF (50 mL) in an oven-dried Schlenk flask. CuBr (20 mg, 0.14 mmol, 0.05 eq) and NaAsc (54 mg, 0.27 mmol, 0.1 eq) were subsequently added. PMDETA (57 µL, 47 mg, ρ = 0.83 g mL^{-1}, 0.27 mmol, 0.1 eq) was added and the solution was mixed quickly by shaking before being rapidly frozen in liquid nitrogen. The solution was then degassed by three cycles of freeze-pump-thaw. After degassing, the reaction was placed in an oil bath preheated at 50 ºC and allowed to stir for 2 hr before the reaction was exposed to air and dried *in vacuo* to yield **TBDMS-*M*$_2$** as a pale yellow solid (2.5 g, 2.4 mmol, 88%). *Anal.*: R_f = 0.55 (1:1 EtOAc:Hex); ^{1}H NMR (500 MHz, $CDCl_3$) δ / ppm, 8.17 – 8.11 (m, 1H), 7.29 (s, 0H), 7.11 – 7.04 (m, 2H), 6.95 – 6.87 (m, 2H), 6.79 (dt, J = 9.3, 3.4 Hz, 2H), 4.32 (t, J = 7.2 Hz, 1H), 4.06 (t, J = 6.2 Hz, 2H), 3.95 (td, J = 6.5, 4.7 Hz, 2H), 3.54 (t, J = 6.8 Hz, 1H), 2.82 (t, J = 5.2 Hz, 1H), 2.64 (s, 3H), 2.33 (t, J = 7.0 Hz, 1H), 1.98 – 1.90 (m, 1H), 1.89 (dd, J = 6.7, 3.3 Hz, 3H), 1.78 (dt, J = 14.3, 6.9 Hz, 3H), 1.76 – 1.68 (m, 1H), 1.52 – 1.39 (m, 3H), 1.36 (td, J = 7.2, 4.2 Hz, 5H), 0.93 (s, 4H), 0.09 (s, 3H); ^{13}C NMR (126 MHz, $CDCl_3$) δ / ppm, 165.88, 162.45, 156.84, 144.39, 133.71, 122.78, 122.76, 120.65, 117.82, 117.78, 115.24, 111.70, 68.50, 68.43, 67.81, 67.60, 50.36, 45.30, 32.76, 30.48, 29.37, 29.35, 29.26, 29.09, 28.97, 28.81, 28.36, 26.97, 26.61, 26.24, 26.11, 26.07, 25.49, 25.37, 22.67, 19.72, 16.67, -4.28.

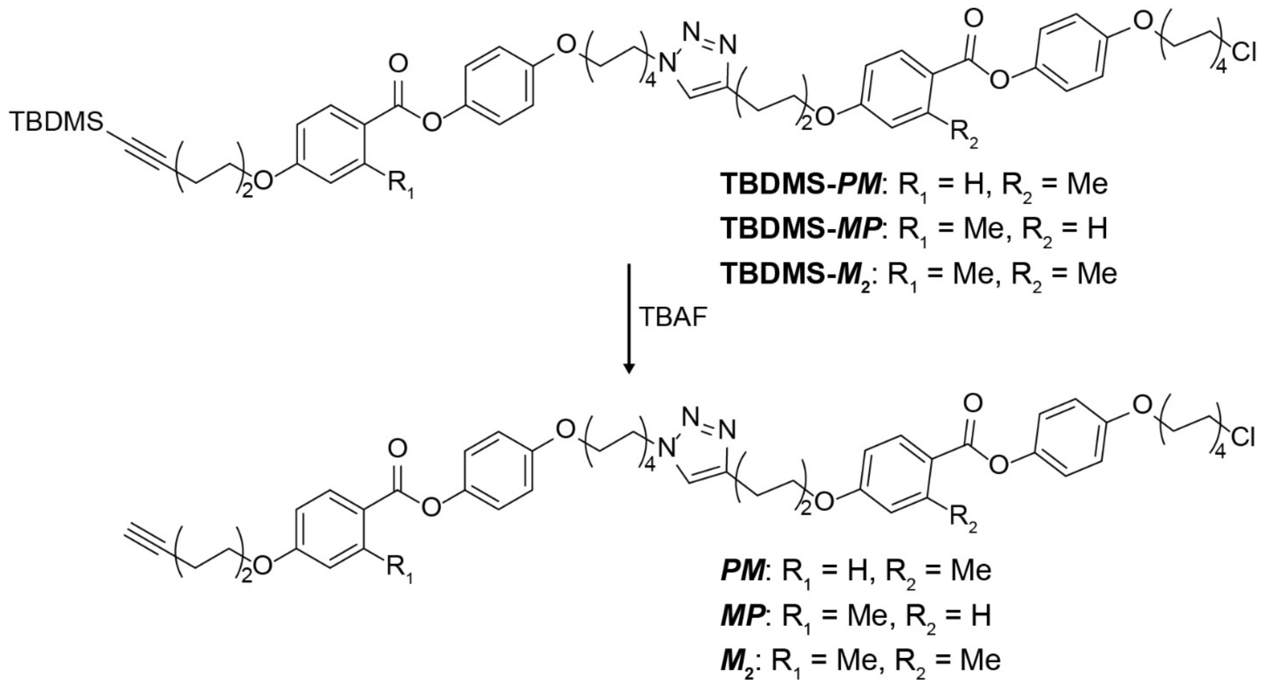

Scheme S7: Chemical scheme towards the deprotection reaction to prepare *PM*, *MP*, and *M*$_2$

PM: **TBDMS-*PM*** (1.3 g, 1.2 mmol, 1 eq) was dissolved in DMAc (78 mL, 16.7 g mL^{-1}) and a TBAF (3.7 mL, 0.35 mol dm^{-3}, 1.3 mmol, 1.05 eq) solution in THF was added. The reaction mixture was stirred at room temperature for 5 mins before being quenched with brine (150 mL). The product was then extracted with DCM (100 mL, 2 × 50 mL), dried over Na_2SO_4 and concentrated *in vacuo*. The crude product was purified thrice *via* flash chromatography (4:1 EtOAc:Hex, then 3:2 EtOAc:Hex during the first column and 3:2 EtOAc:Hex for the second column; note that the last column was necessitated by degradation during an overnight drying step *in vacuo* at 90 °C). The product was then dried *in vacuo* to yield ***PM*** as a white powder (670 mg, 0.71 mmol, 57 %). *Anal.*: R_f = 0.58 (3:2 EtOAc:Hex); ^{1}H NMR (500 MHz, $CDCl_3$) δ / ppm, 8.17 – 8.10 (m, 1H), 7.29 (s, 0H), 7.13 – 7.04 (m, 2H), 6.99 – 6.93 (m, 1H), 6.96 – 6.87 (m, 2H), 6.81 – 6.76 (m, 1H), 4.32 (t, *J* = 7.2 Hz, 1H), 4.11 – 4.02 (m, 2H), 3.95 (td, *J* = 6.5, 4.1 Hz, 2H), 3.54 (t, *J* = 6.7 Hz, 1H), 2.84 – 2.78 (m, 1H), 2.64 (s, 1H), 2.30 (td, *J* = 7.0, 2.7 Hz, 1H), 2.01 – 1.85 (m, 4H), 1.77 (tt, *J* = 10.2, 7.0 Hz, 4H), 1.52 – 1.40 (m, 3H), 1.43 – 1.31 (m, 5H); ^{13}C NMR (126 MHz, $CDCl_3$) δ / ppm, 165.88, 165.46, 163.42, 162.45, 156.86, 147.84, 144.55, 144.50, 144.39, 133.71, 132.37, 122.76, 122.64, 121.99, 120.75, 120.64, 117.78, 115.24, 115.21, 114.38, 111.69, 84.06, 68.94, 68.50, 68.41, 67.81, 67.73, 50.35, 45.30, 32.76, 30.48, 29.37, 29.35, 29.34, 29.26, 29.08, 28.97, 28.81, 28.26, 26.97, 26.60, 26.11, 26.10, 26.07, 25.50, 25.10, 22.67, 18.30.

MP: **TBDMS-*MP*** (1.2 g, 1.1 mmol, 1 eq) was dissolved in DMAc (72 mL, 16.7 g mL^{-1}) and a TBAF (3.4 mL, 0.35 mol dm^{-3}, 1.20 mmol, 1.05 eq) solution in THF was added. The reaction mixture was stirred at room temperature for 5 mins before being quenched with brine (150 mL). The product was then extracted with DCM (100 mL, 2 × 50 mL), dried over Na_2SO_4, and concentrated *in vacuo*. The crude product was purified twice *via* flash chromatography (4:1 EtOAc:Hex, then 3:2 EtOAc:Hex during the first column and 3:2 EtOAc:Hex for the second

column; note that the second column was necessitated by degradation during an overnight drying step *in vacuo* at 90 °C). The product was then dried *in vacuo* to yield ***MP*** as a pale yellow solid (540 mg, 0.58 mmol, 51 %). *Anal.*: R_f = 0.5 (3:2 EtOAc:Hex); ^{1}H NMR (500 MHz, $CDCl_3$) δ / ppm, 8.17 – 8.09 (m, 1H), 7.13 – 7.04 (m, 2H), 6.98 – 6.87 (m, 3H), 6.82 – 6.73 (m, 1H), 4.32 (t, *J* = 7.3 Hz, 1H), 4.06 (q, *J* = 6.1 Hz, 2H), 3.95 (q, *J* = 6.3 Hz, 2H), 3.54 (t, *J* = 6.7 Hz, 1H), 2.84 – 2.78 (m, 1H), 2.64 (s, 1H), 2.30 (td, *J* = 7.0, 2.6 Hz, 1H), 2.01 – 1.93 (m, 1H), 1.93 – 1.87 (m, 3H), 1.83 – 1.69 (m, 4H), 1.50 – 1.40 (m, 3H), 1.39 – 1.33 (m, 4H); ^{13}C NMR (126 MHz, $CDCl_3$) δ / ppm, 165.88, 165.45, 163.43, 162.44, 156.89, 156.82, 147.84, 144.54, 144.52, 144.39, 133.71, 132.37, 122.78, 122.62, 121.97, 120.62, 117.80, 115.24, 115.22, 114.37, 111.69, 84.09, 68.91, 68.48, 68.43, 68.00, 67.54, 50.34, 45.30, 32.76, 30.48, 29.38, 29.36, 29.33, 29.26, 29.08, 28.97, 28.80, 28.27, 26.97, 26.61, 26.11, 26.11, 26.06, 25.51, 25.10, 22.66, 18.29.

***M*2**: **TBDMS-*M*2** (1.2 g, 1.2 mmol, 1 eq) was dissolved in DMAc (74 mL, 16.7 g mL^{-1}) and a TBAF (3.5 mL, 0.35 mol dm^{-3}, 1.2 mmol, 1.05 eq) solution in THF was added. The reaction mixture was stirred at room temperature for 5 mins before being quenched with brine (150 mL). The product was then extracted with DCM (100 mL, 2 × 50 mL), dried over Na_2SO_4 and concentrated *in vacuo*. The crude product was purified thrice *via* flash chromatography (1:4 EtOAc:Hex, then 3:2 EtOAc:Hex during the first column and 3:2 EtOAc:Hex for the second column; note that the last column was necessitated by degradation during an overnight drying step *in vacuo* at 90 °C). The product was then dried *in vacuo* to yield ***M*2** as a yellow liquid that slowly crystallized into a pale yellow powder (565 mg, 0.59 mmol, 51 %). *Anal.*: R_f = 0.56 (3:2 EtOAc:Hex); ^{1}H NMR (500 MHz, $CDCl_3$) δ / ppm, 8.17 – 8.11 (m, 1H), 7.33 (s, 0H), 7.11 – 7.04 (m, 2H), 6.95 – 6.87 (m, 2H), 6.79 (td, J = 6.2, 2.6 Hz, 2H), 4.33 (t, J = 7.2 Hz, 1H), 4.06 (t, J = 6.2 Hz, 2H), 3.95 (td, J = 6.5, 3.8 Hz, 2H), 3.54 (t, J = 6.7 Hz, 1H), 2.86 (t, J = 7.0 Hz, 1H), 2.64 (s, 3H), 2.30 (td, J = 7.0, 2.6 Hz, 1H), 2.00 – 1.88 (m, 4H), 1.83 – 1.69 (m, 4H), 1.45 (dh, J = 13.0, 6.9 Hz, 3H), 1.41 – 1.33 (m, 5H); ^{13}C NMR (126 MHz, $CDCl_3$) δ / ppm, 165.89, 165.87, 162.45, 162.41, 156.85, 156.81, 147.41, 144.52, 144.50, 144.39, 133.72, 122.78, 122.76, 120.79, 120.75, 117.80, 117.77, 115.24, 111.69, 84.09, 68.91, 68.50, 68.41, 67.74, 67.54, 50.77, 45.30, 32.76, 30.36, 29.37, 29.35, 29.31, 29.22, 29.05, 28.97, 28.77, 28.27, 26.97, 26.55, 26.10, 26.05, 26.02, 25.19, 25.10, 22.66, 18.29.

Section S2: Chemical characterization

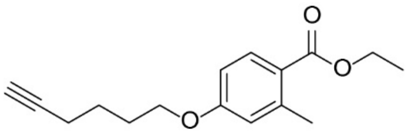

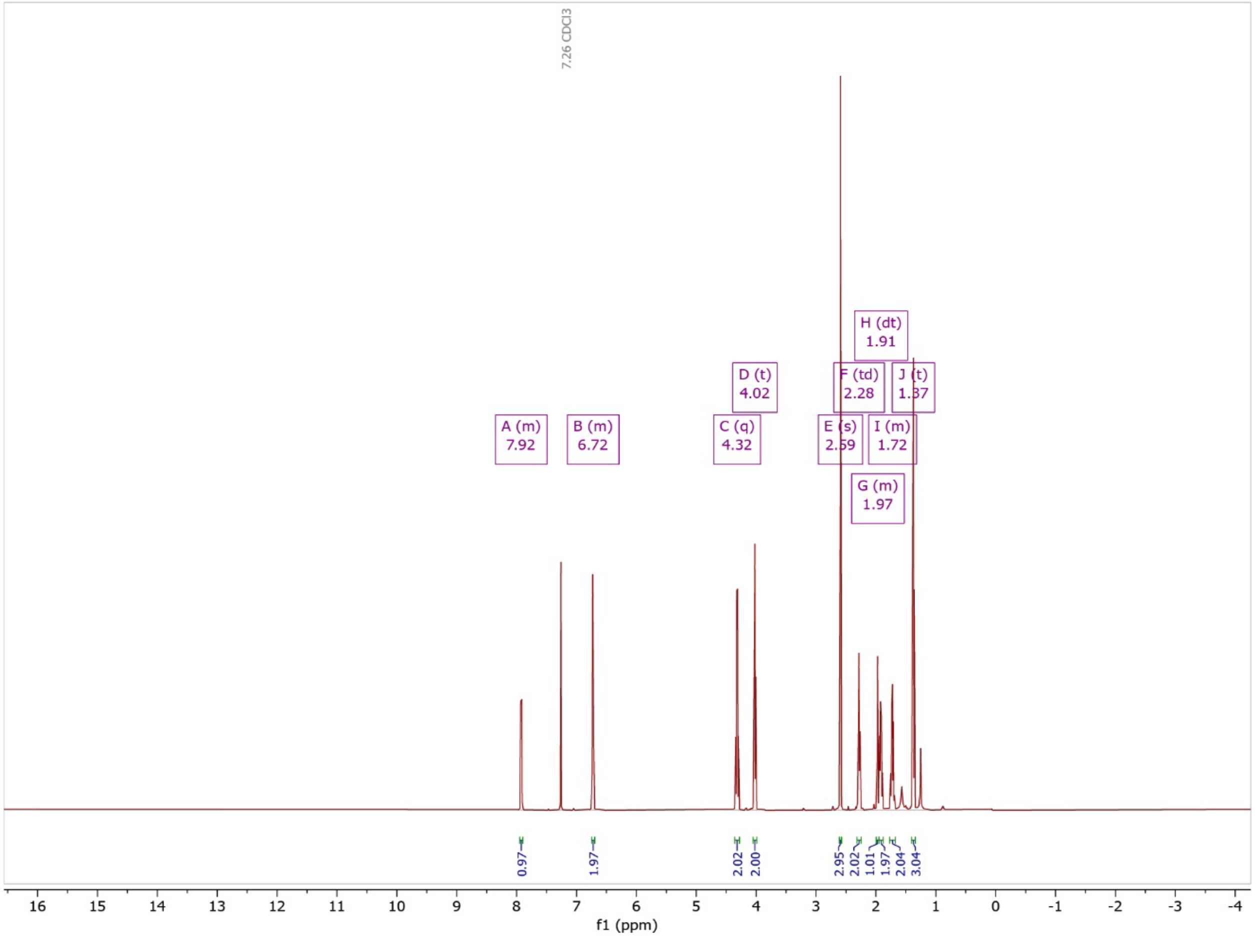

Figure S1: ^{1}H NMR of the first fragment towards the synthesis of M_1

^{1}H NMR spectrum (500 MHz, $CDCl_3$) of a fragment corresponding to **1** in **Scheme S1**.

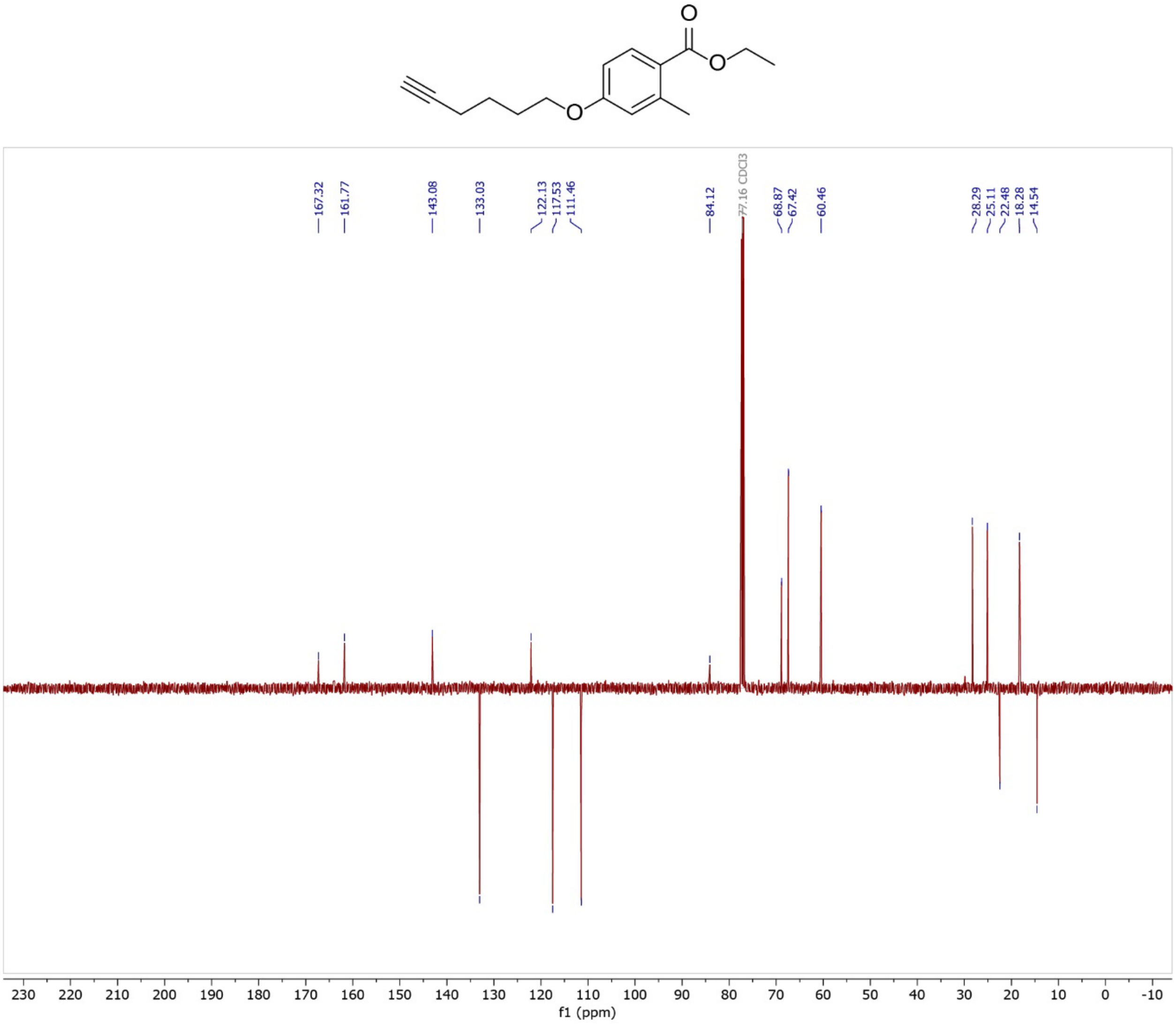

Figure S2: ^{13}C NMR of the first fragment towards the synthesis of *M*$_1$

Attached proton test (APT) ^{13}C NMR spectrum (126 MHz, $CDCl_3$) of a fragment corresponding to **1** in **Scheme S1**.

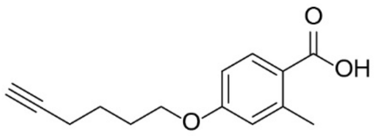

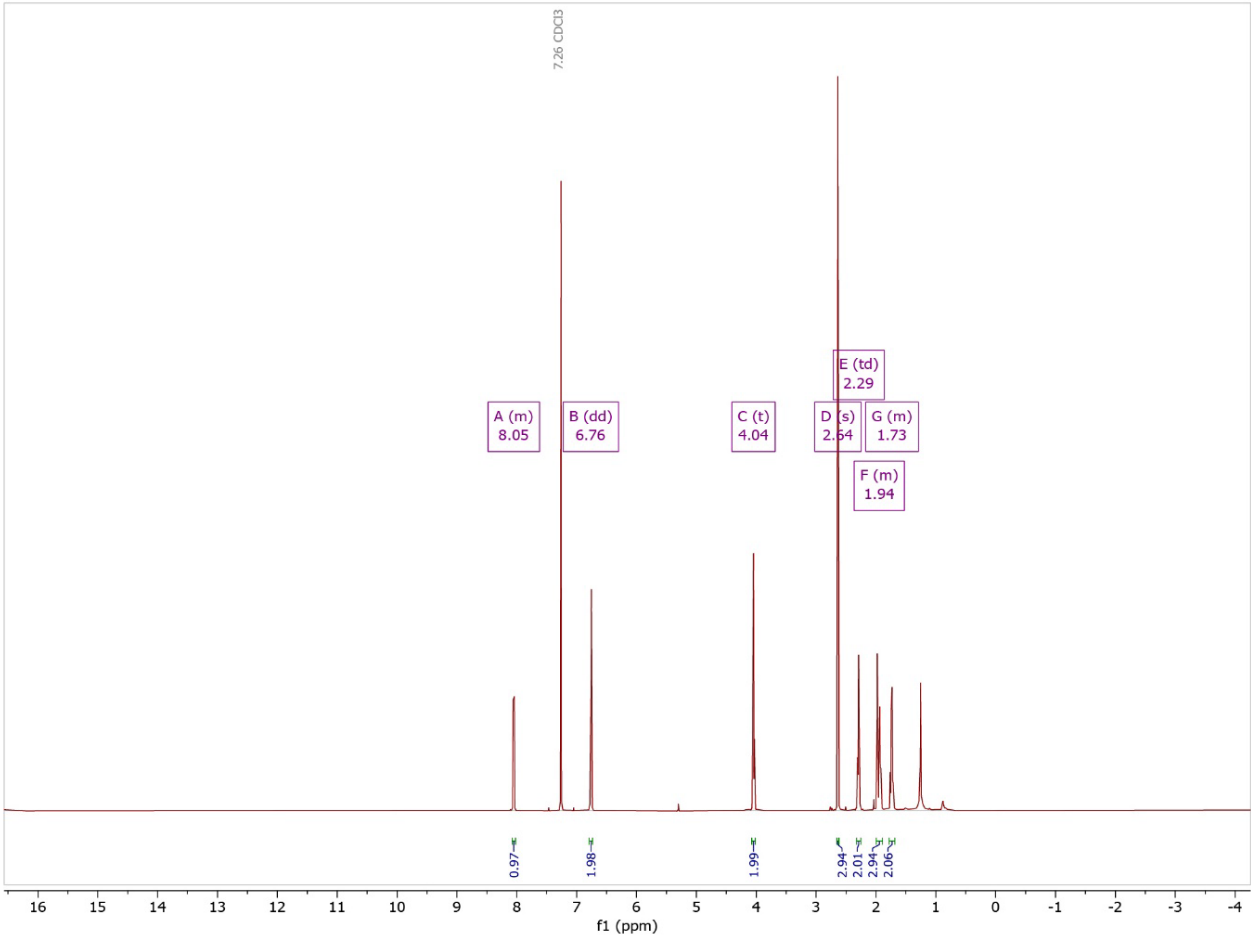

Figure S3: ^{1}H NMR of the second fragment towards the synthesis of *M*$_1$

^{1}H NMR spectrum (500 MHz, $CDCl_3$) of a fragment corresponding to **2** in **Scheme S1**.

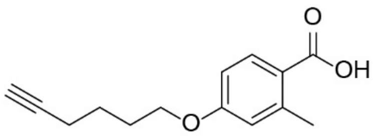

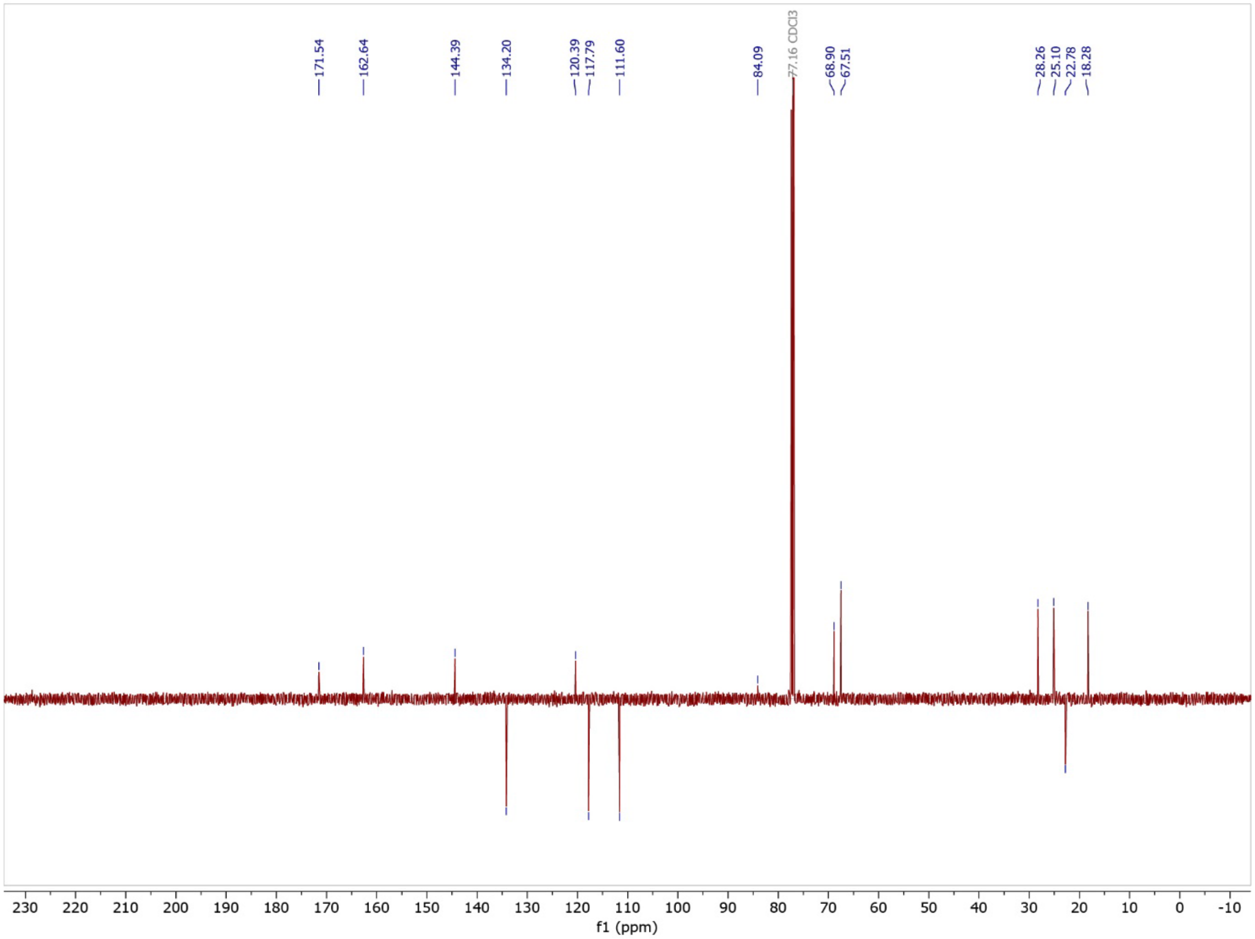

Figure S4: ^{13}C NMR of the second fragment towards the synthesis of M_1

^{13}C NMR spectrum (APT, 126 MHz, $CDCl_3$) of a fragment corresponding to **2** in **Scheme S1**.

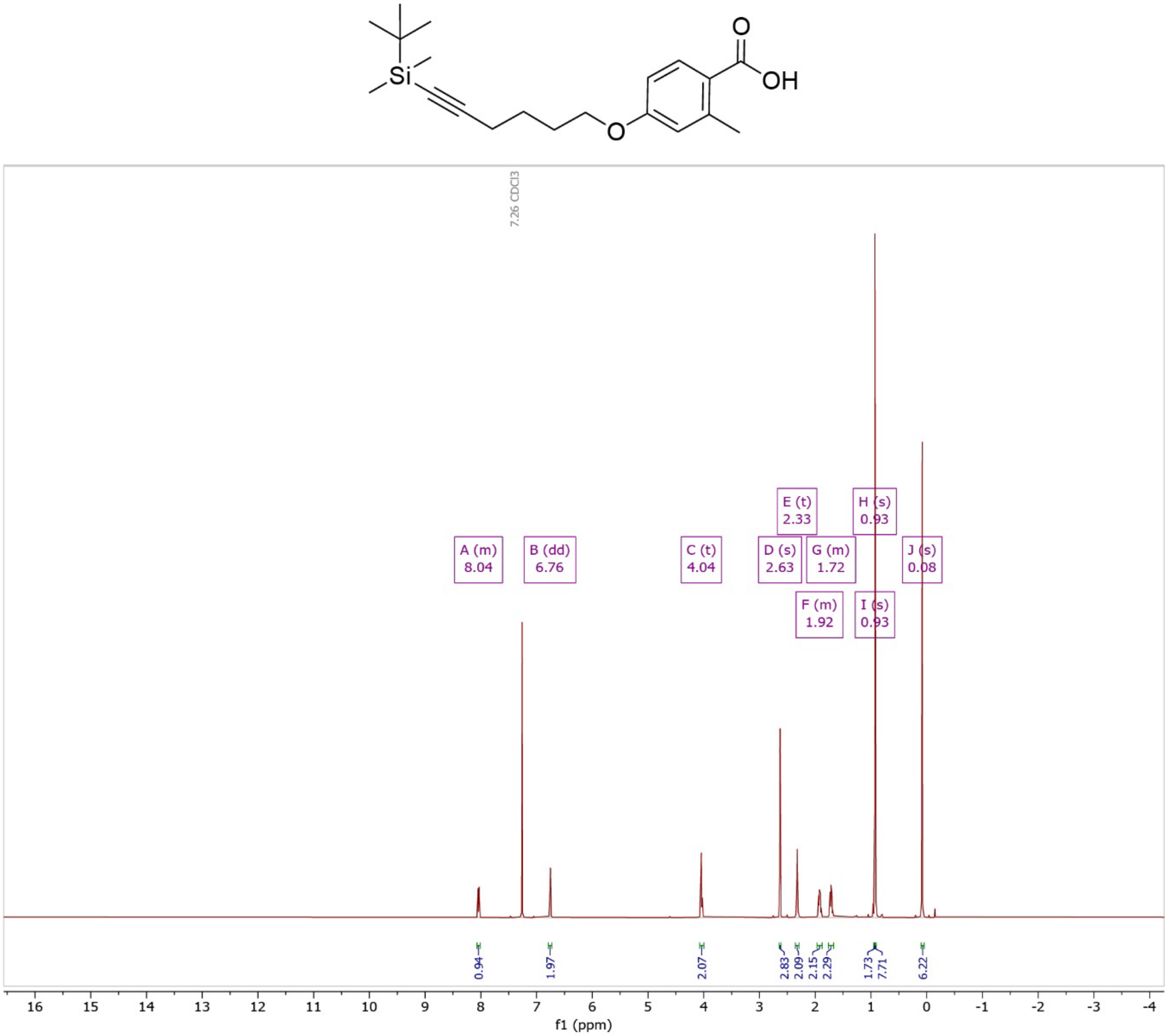

Figure S5: ^{1}H NMR of a TBDMS-protected fragment towards the synthesis of TBDMS-protected M_1

^{1}H NMR spectrum (500 MHz, $CDCl_3$) of a fragment corresponding to **3** in **Scheme S2**.

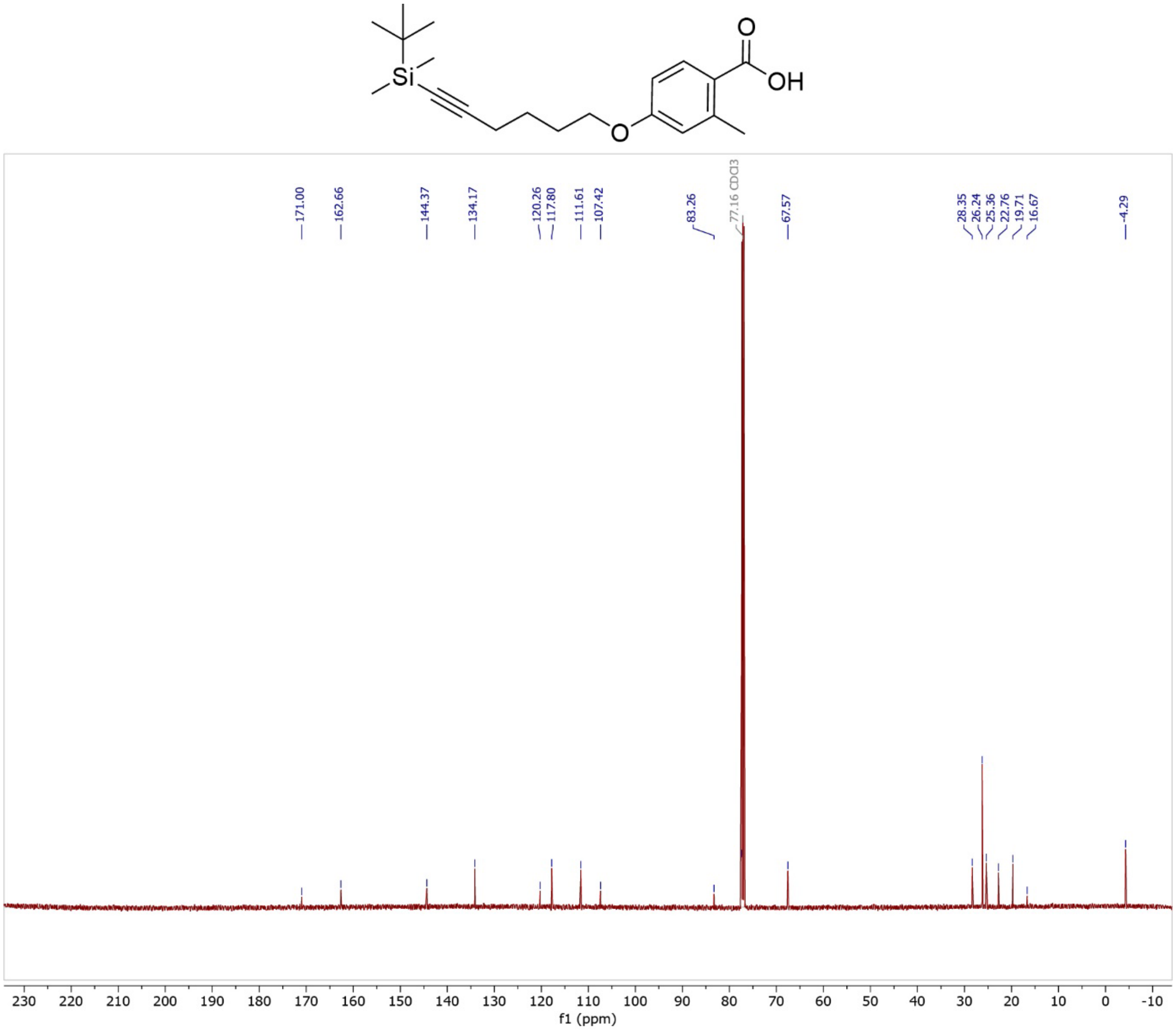

Figure S6: ^{13}C NMR of a fragment towards the synthesis of TBDMS-protected *M*$_1$

^{13}C NMR spectrum (126 MHz, $CDCl_3$) of a fragment corresponding to **3** in **Scheme S2**.

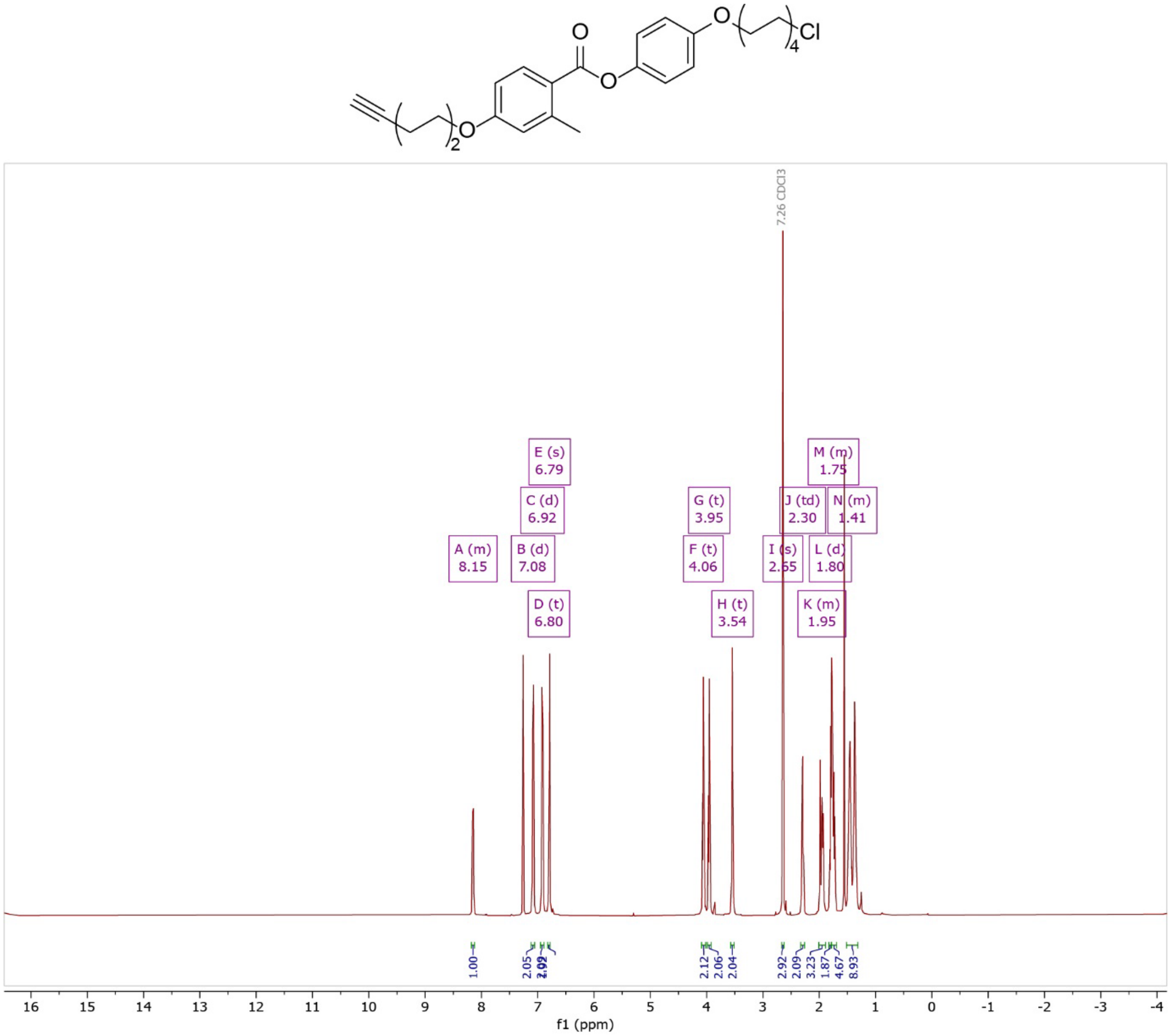

Figure S7: ^{1}H NMR of M_1

^{1}H NMR spectrum (500 MHz, $CDCl_3$) of M_1.

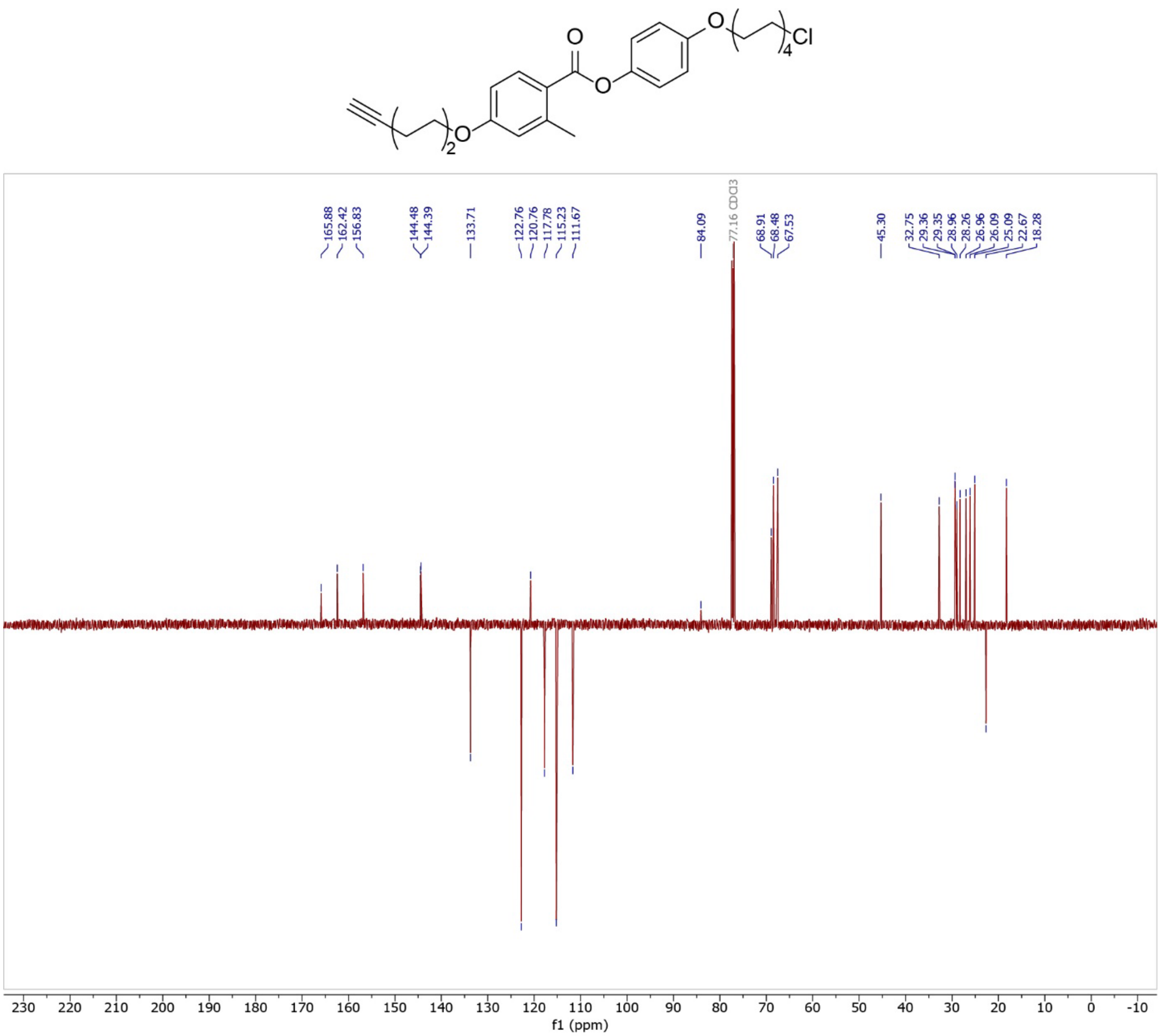

Figure S8: ^{13}C NMR of *M*$_1$

^{13}C NMR spectrum (APT, 126 MHz, $CDCl_3$) of *M*$_1$.

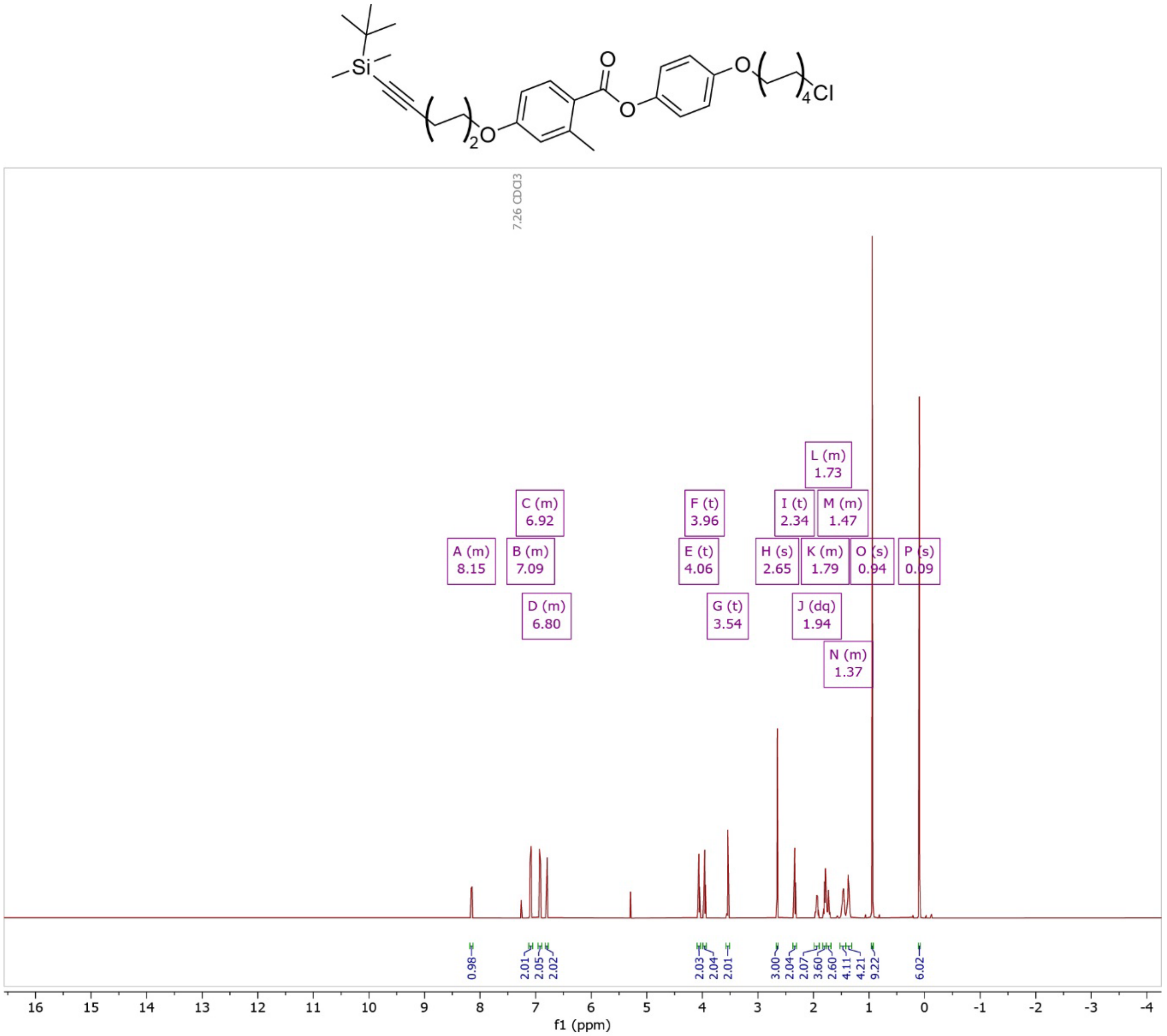

Figure S9: ^{1}H NMR of TBDMS-protected M_1

^{1}H NMR spectrum (500 MHz, $CDCl_3$) of TBDMS-protected M_1.

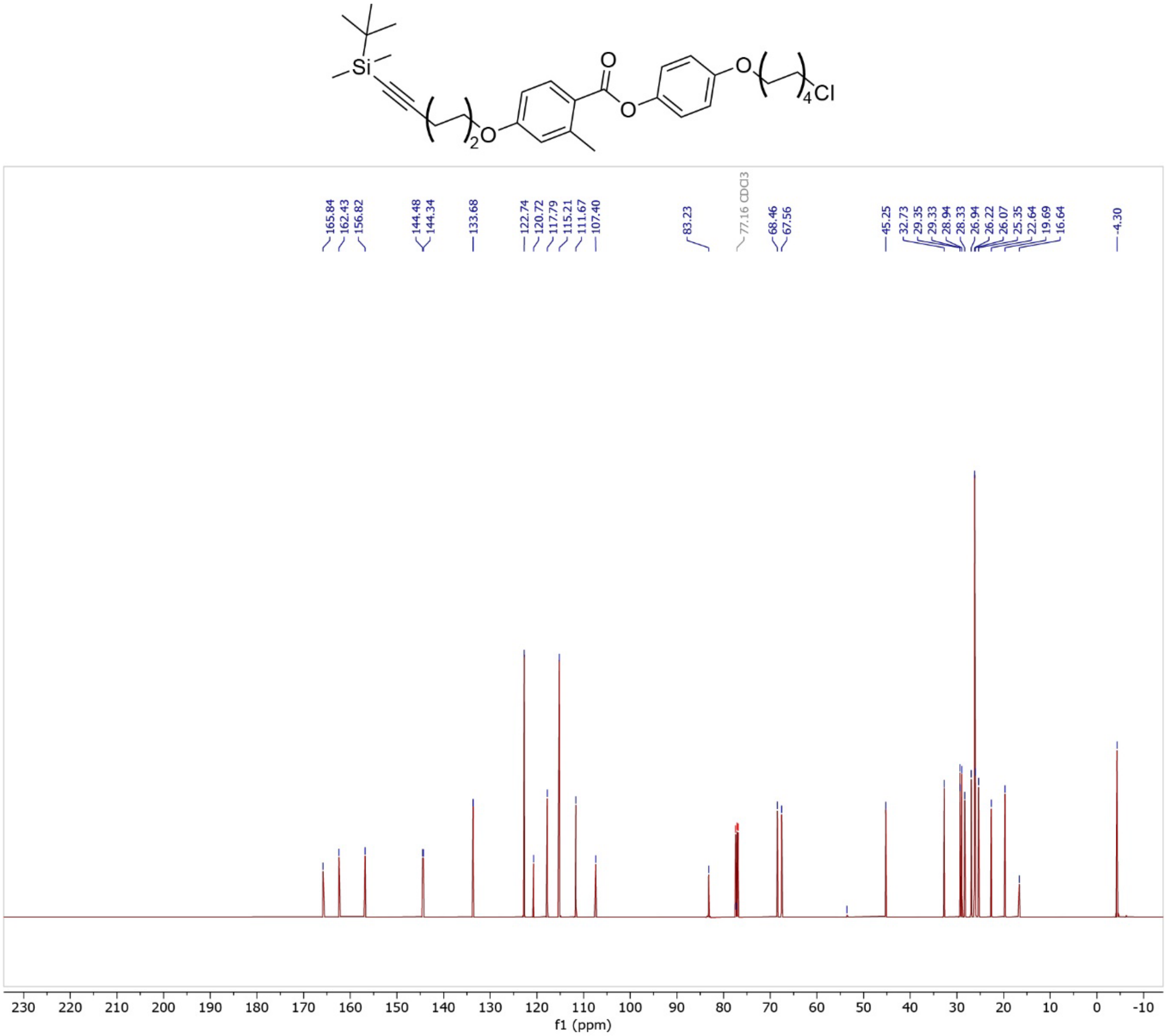

Figure S10: ^{13}C NMR of TBDMS-protected M_1

^{13}C NMR spectrum (126 MHz, $CDCl_3$) of TBDMS-protected M_1.

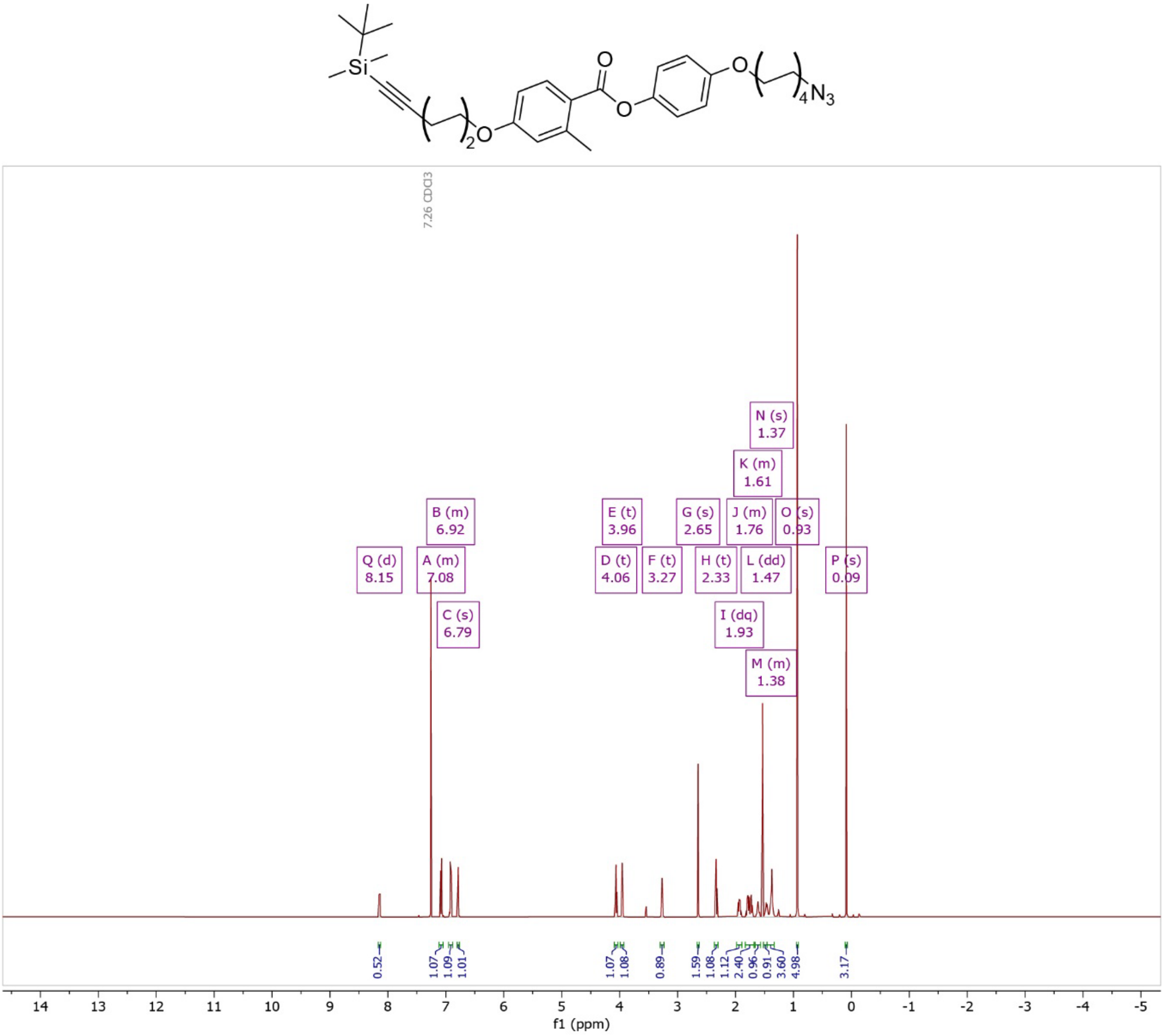

Figure S11: ¹H NMR of TBDMS-*M*₁-N₃

^{1}H NMR spectrum (500 MHz, $CDCl_3$) of TBDMS-M_1-N_3.

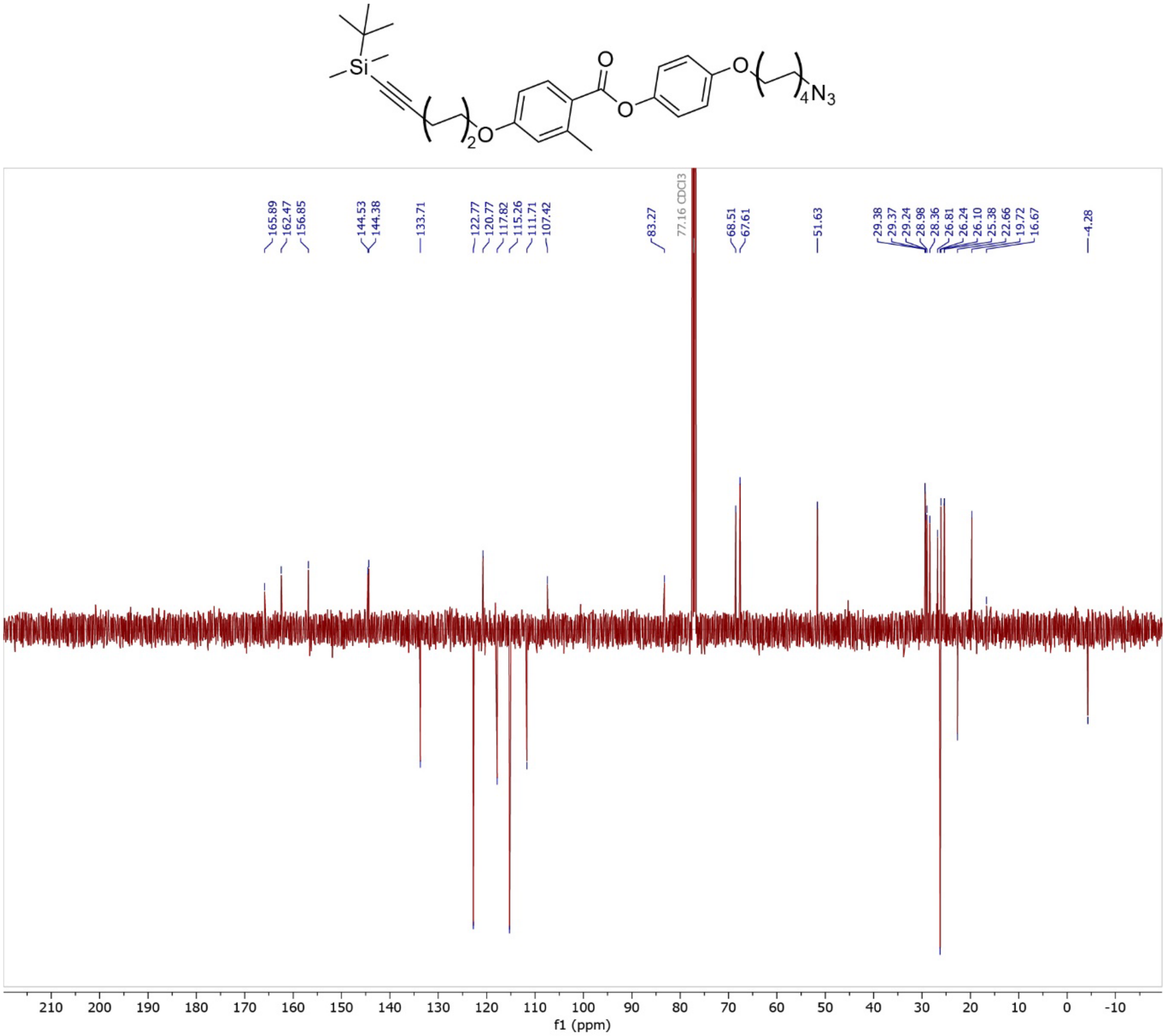

Figure S12: ^{13}C NMR of TBDMS-*M*$_1$-N$_3$

^{13}C NMR spectrum (APT, 126 MHz, $CDCl_3$) of TBDMS-*M*$_1$-N$_3$. The peak corresponding to $CDCl_3$ was truncated for clarity.

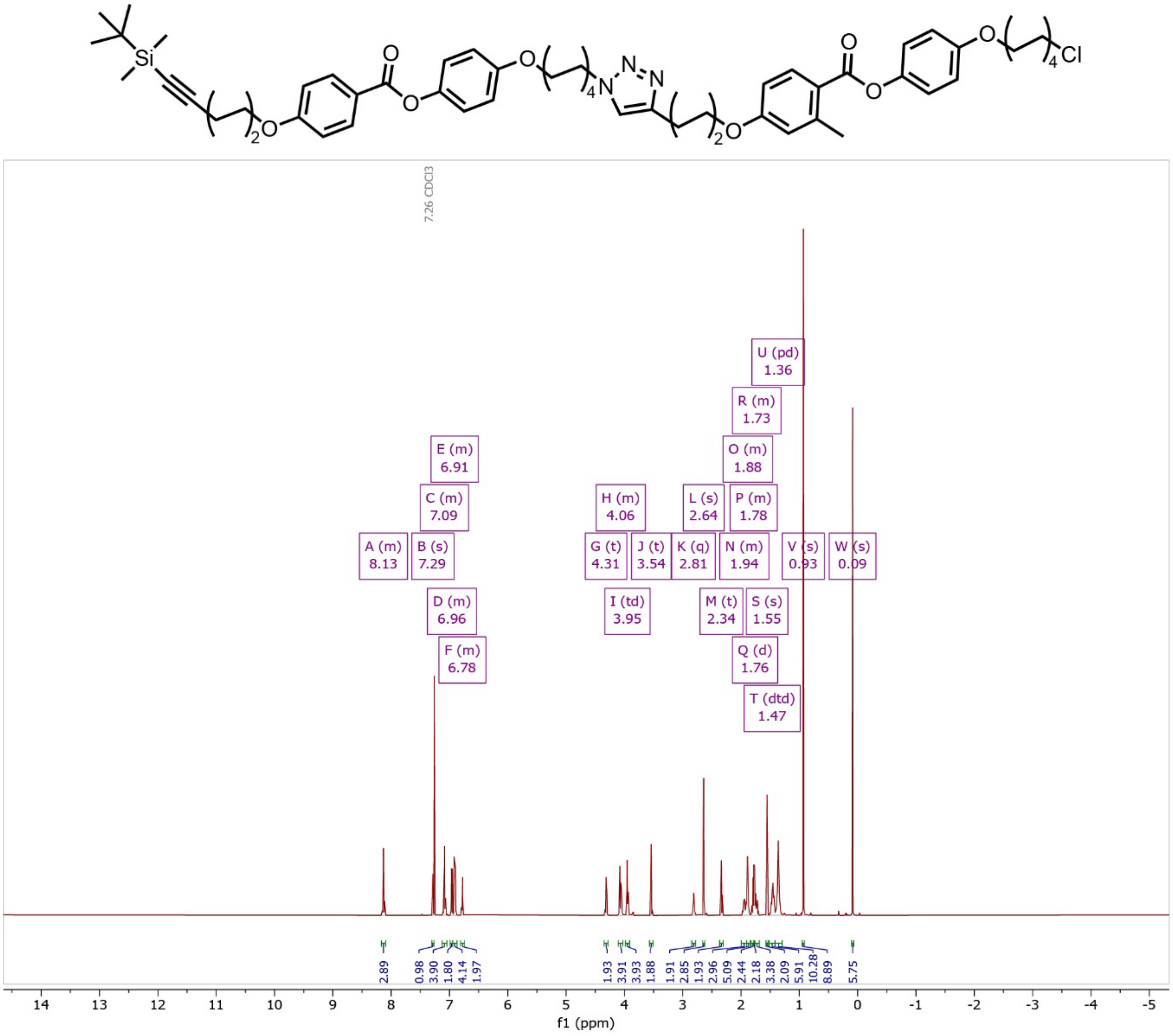

Figure S13: ^{1}H NMR of TBDMS-protected *PM*

^{1}H NMR spectrum (500 MHz, $CDCl_3$) of TBDMS-protected *PM*.

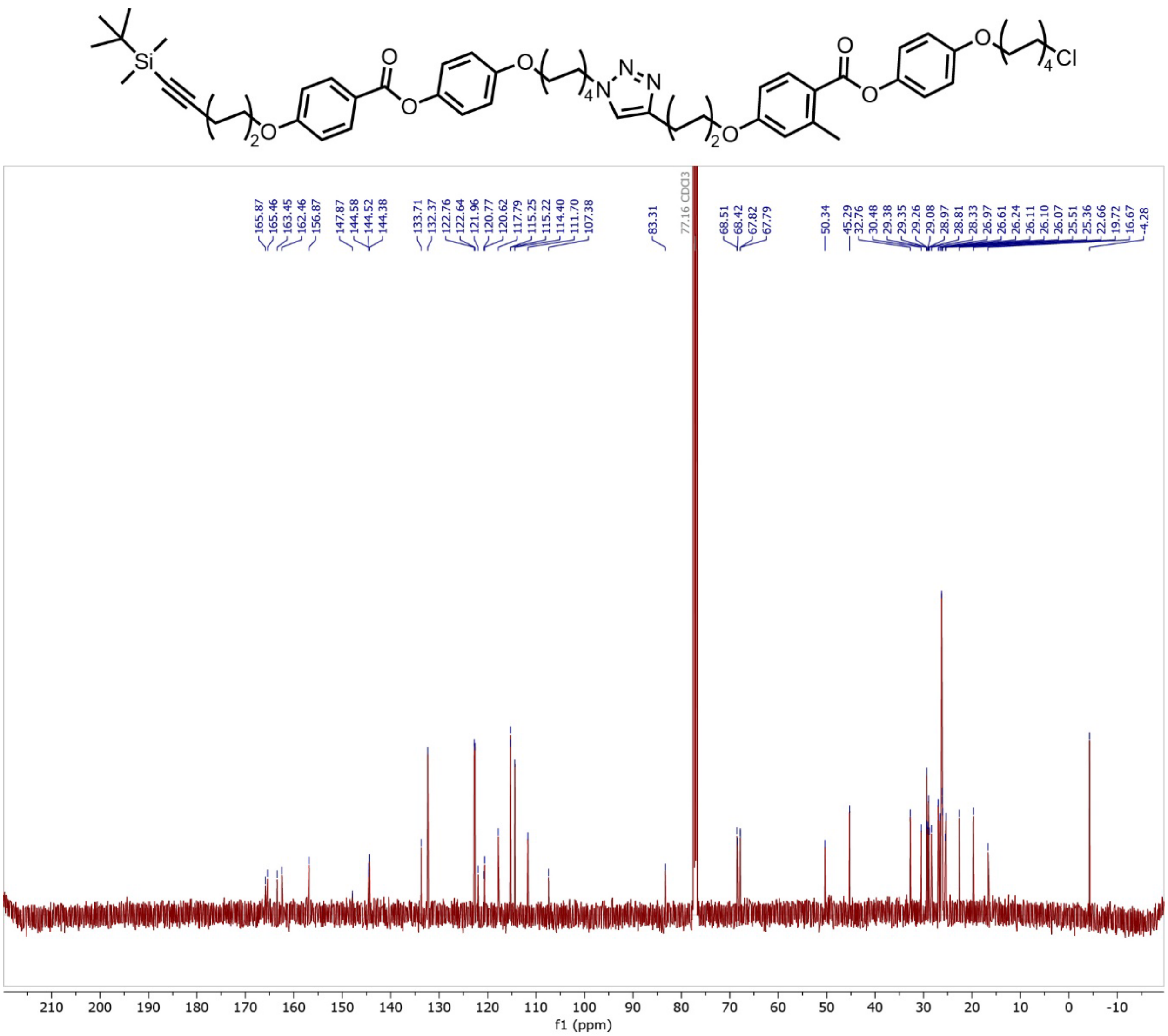

Figure S14: ^{13}C NMR of TBDMS-protected *PM*

^{13}C NMR spectrum (APT, 126 MHz, $CDCl_3$) of TBDMS-protected *PM*. The peak corresponding to $CDCl_3$ was truncated for clarity.

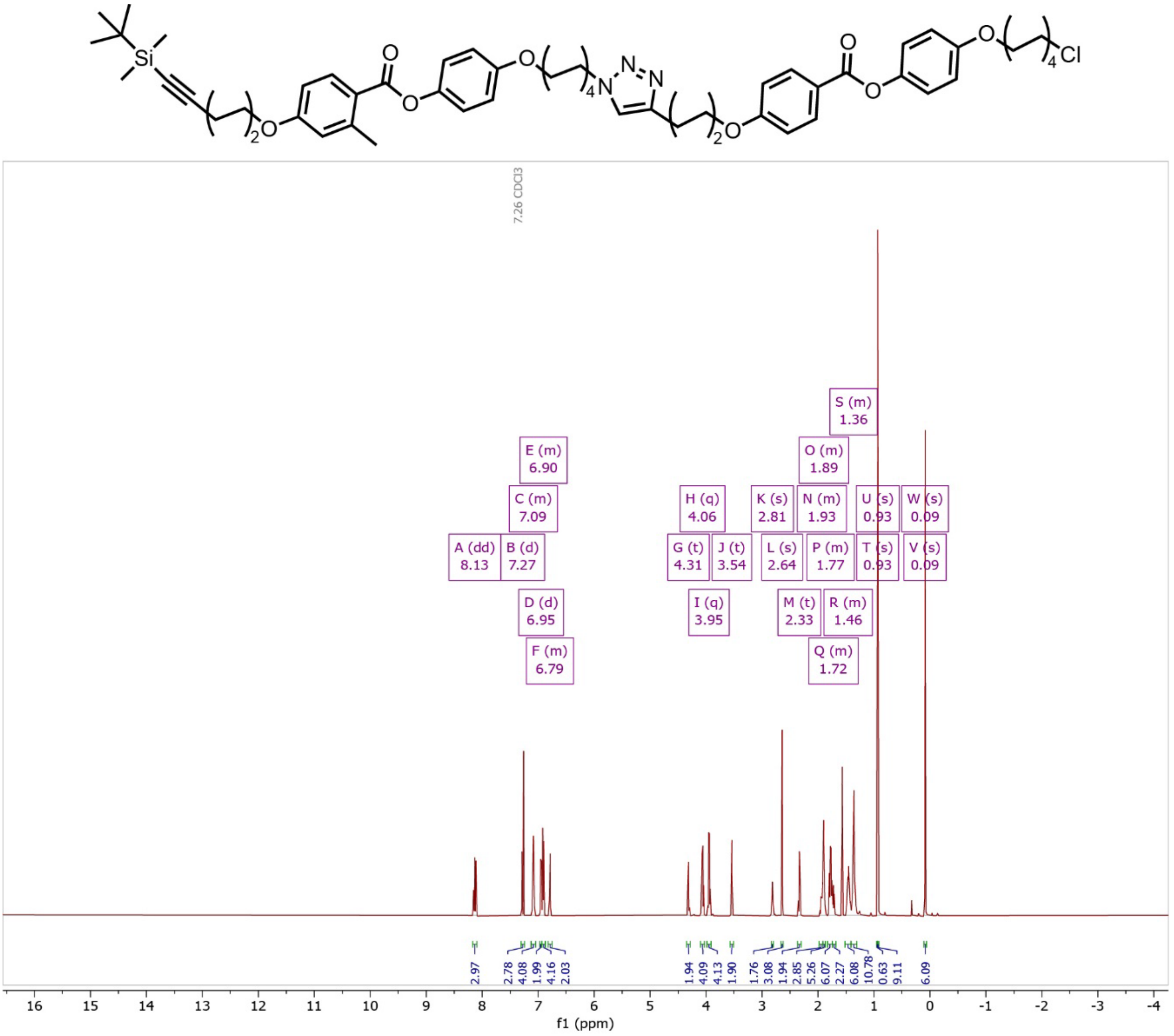

Figure S15: ^{1}H NMR of TBDMS-protected *MP*

^{1}H NMR spectrum (500 MHz, $CDCl_3$) of TBDMS-protected *MP*.

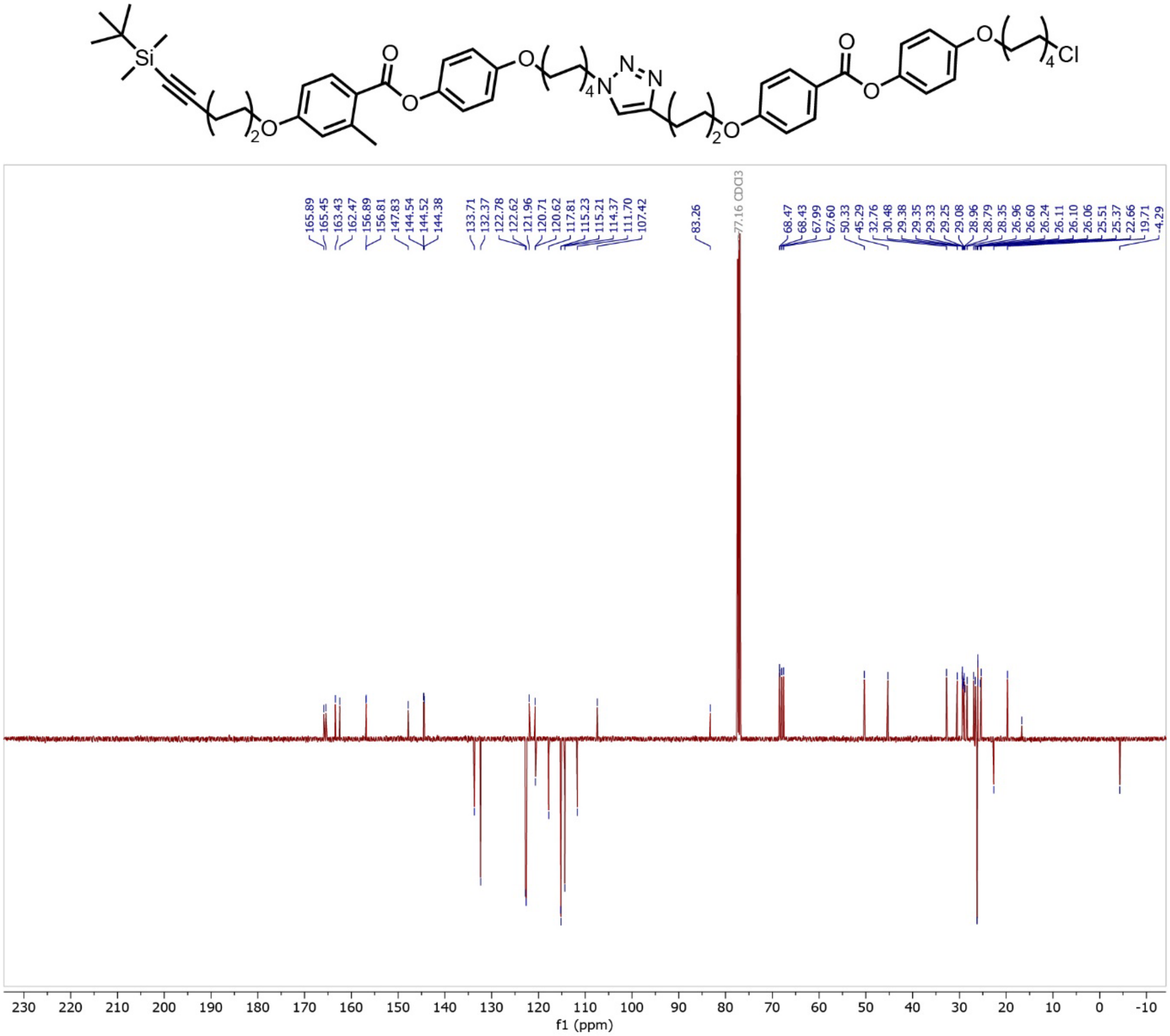

Figure S16: ^{13}C NMR of TBDMS-protected *MP*

^{13}C NMR spectrum (APT, 126 MHz, $CDCl_3$) of TBDMS-protected *MP*.

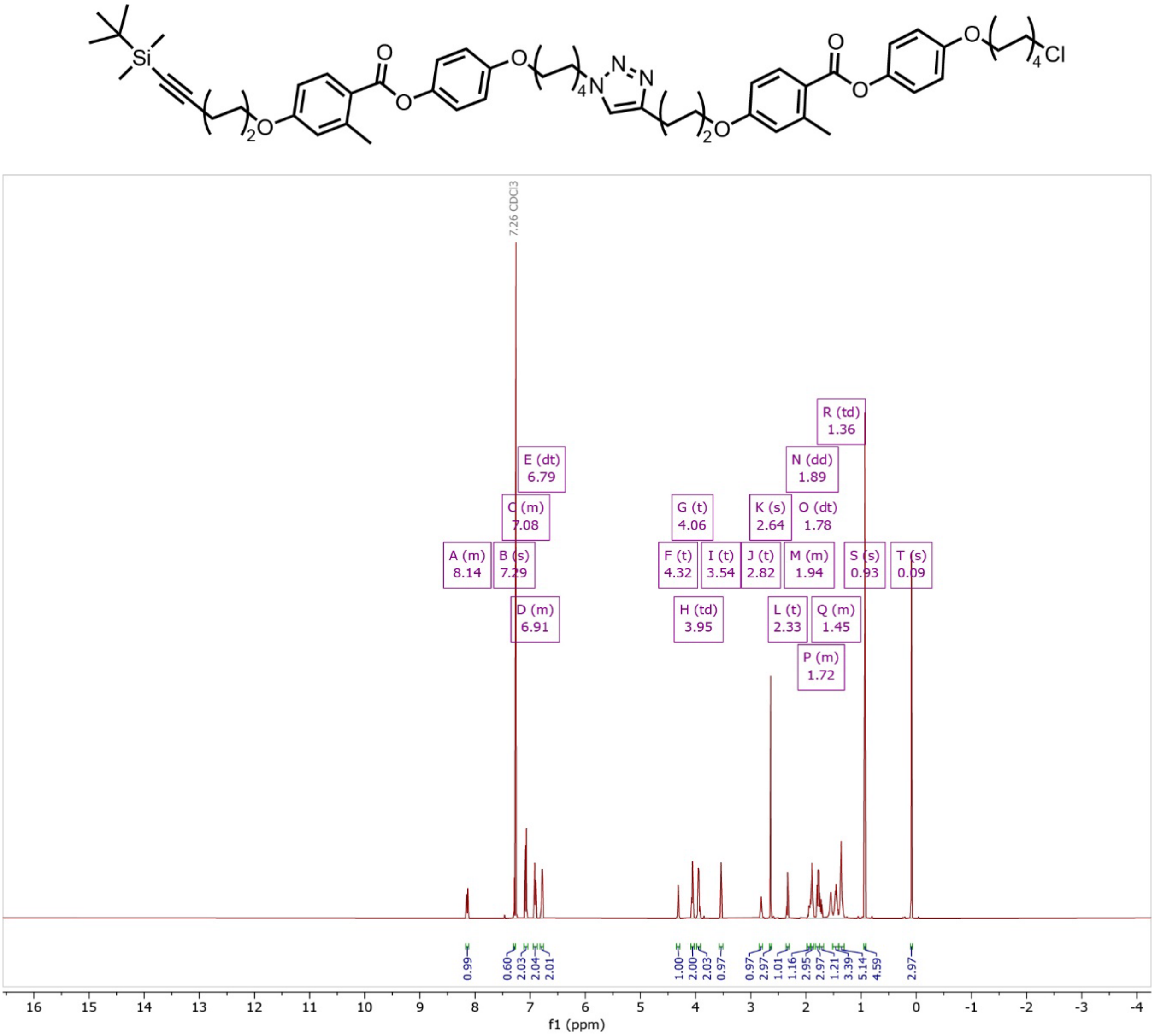

Figure S17: ^{1}H NMR of TBDMS-protected *M₂*

^{1}H NMR spectrum (500 MHz, $CDCl_3$) of TBDMS-protected M_2.

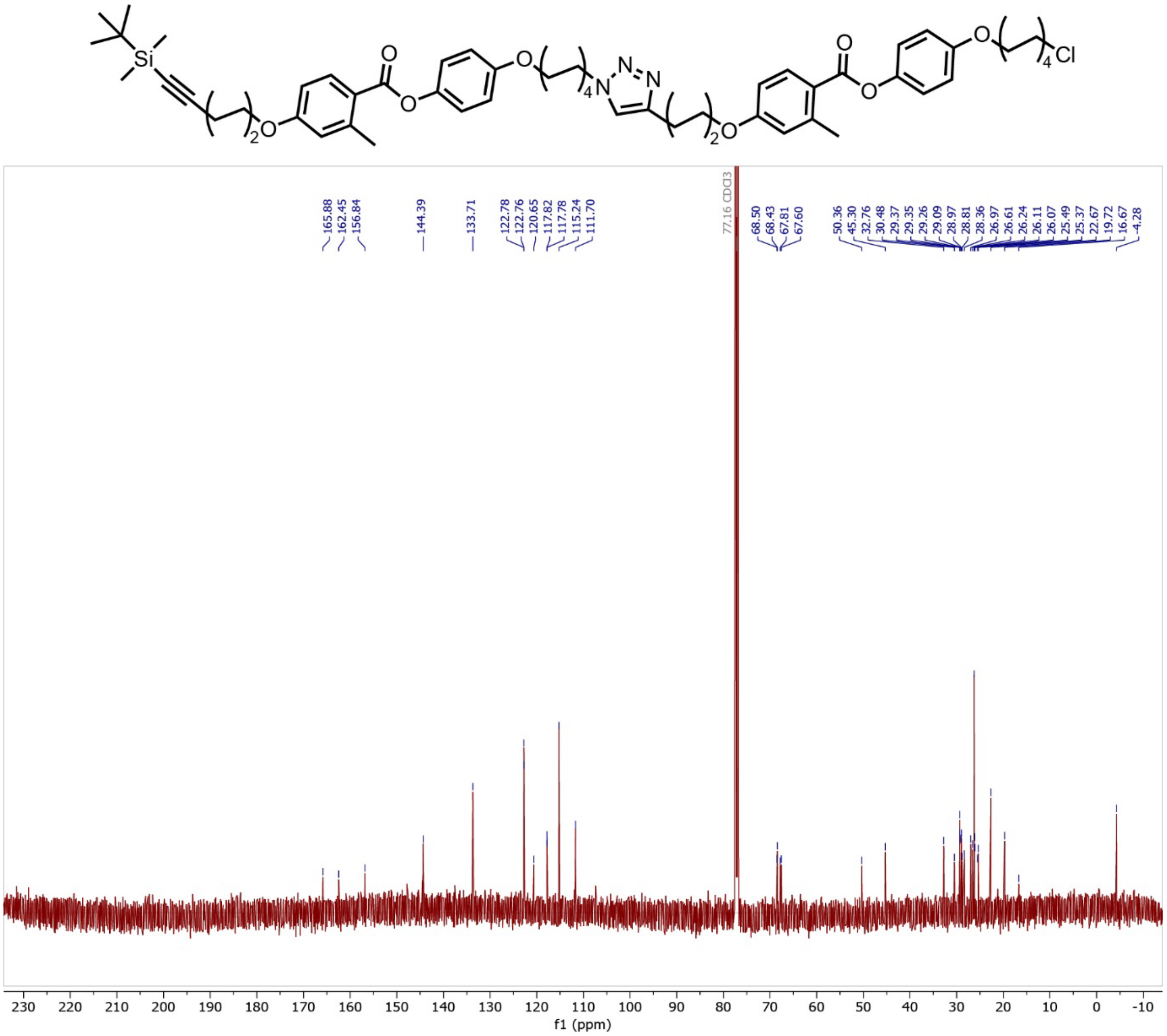

Figure S18: ^{13}C NMR of TBDMS-protected *M_2*

^{13}C NMR spectrum (126 MHz, $CDCl_3$) of TBDMS-protected M_2. The peak corresponding to $CDCl_3$ was truncated for clarity.

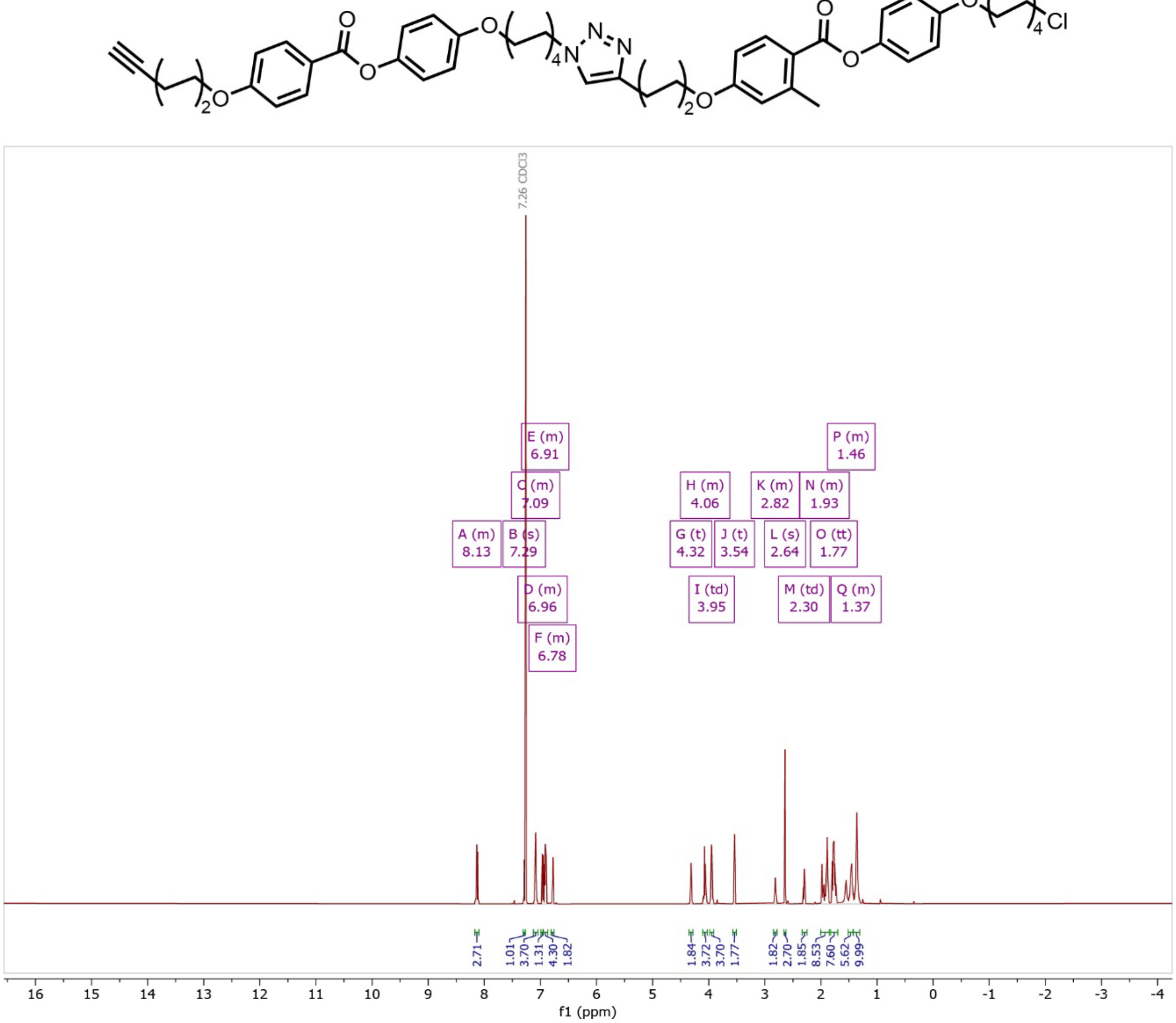

Figure S19: ^{1}H NMR of *PM*

^{1}H NMR spectrum (500 MHz, $CDCl_3$) of *PM*.

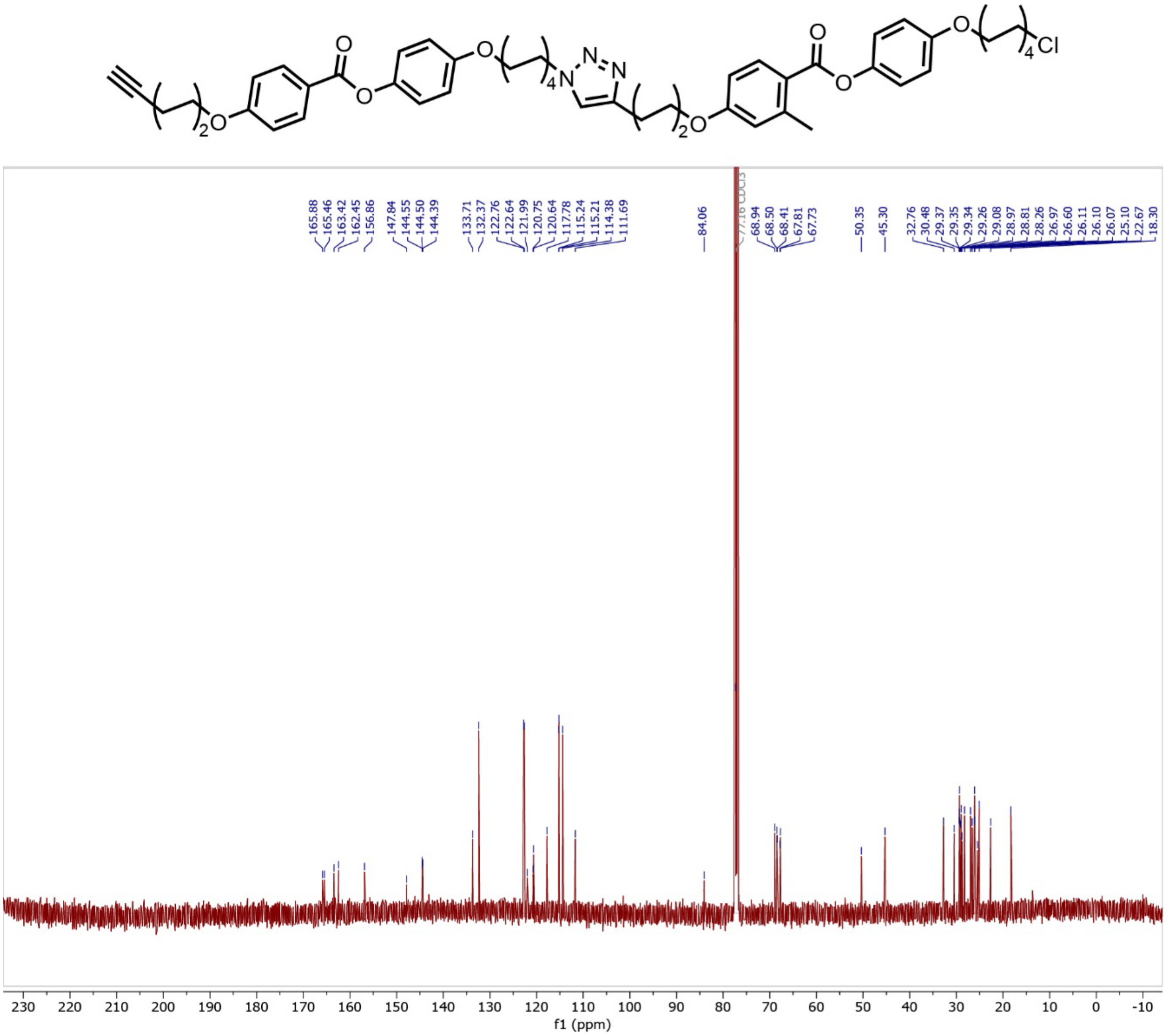

Figure S20: ^{13}C NMR of *PM*

^{13}C NMR spectrum (126 MHz, $CDCl_3$) of *PM*. The peak corresponding to $CDCl_3$ was truncated for clarity.

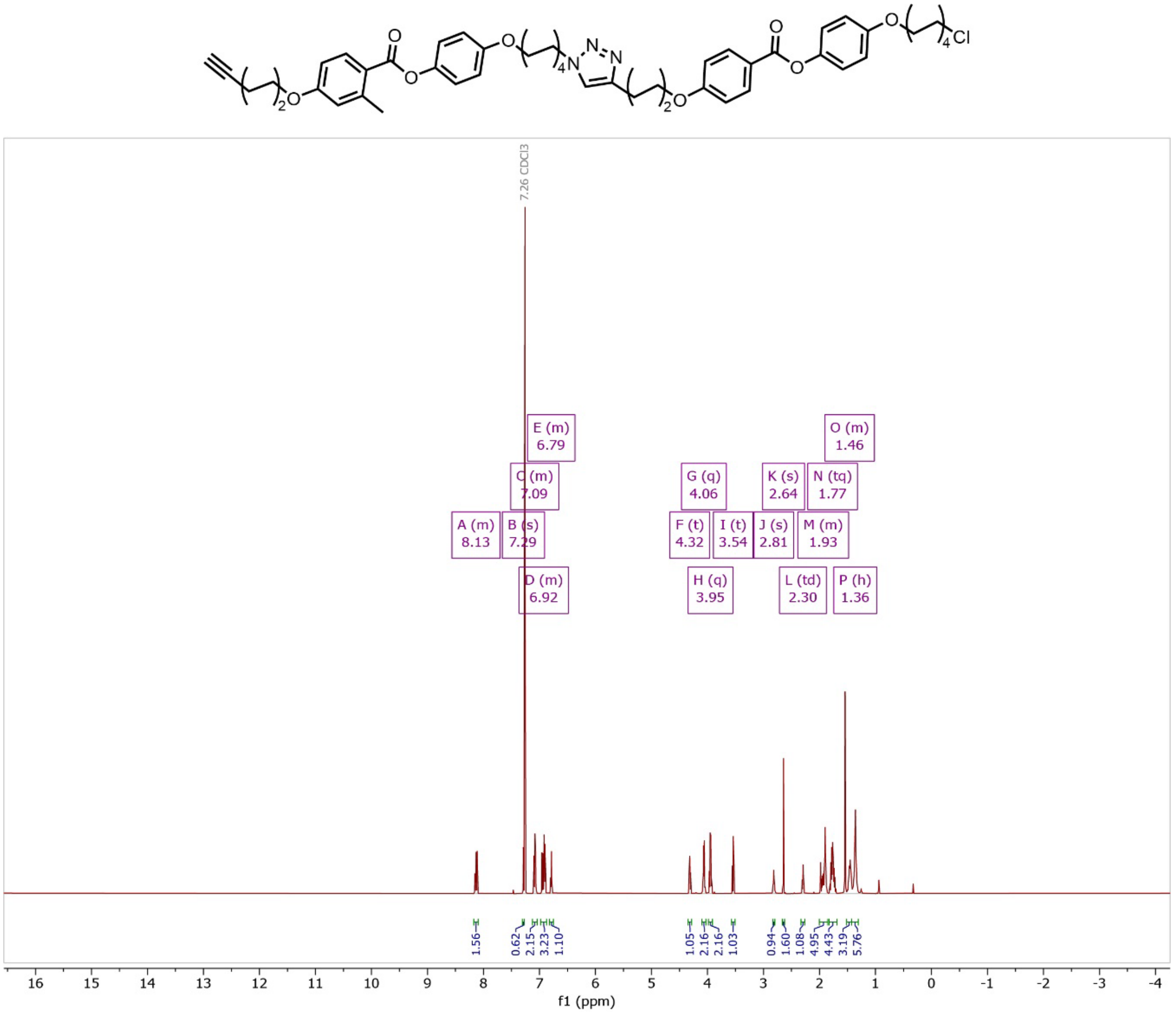

Figure S21: ^{1}H NMR of *MP*

^{1}H NMR spectrum (500 MHz, $CDCl_3$) of *MP*.

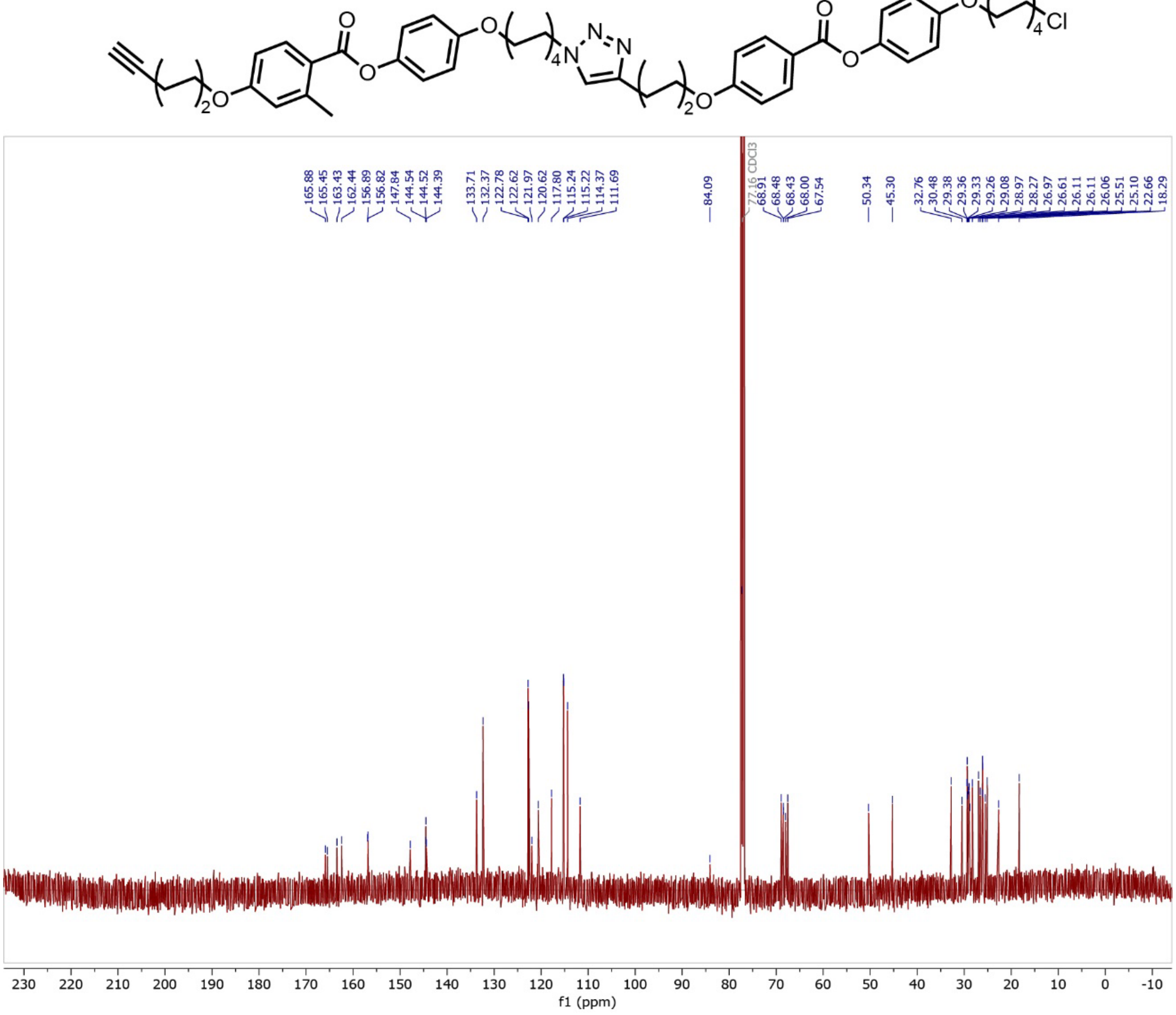

Figure S22: ^{13}C NMR of *MP*

^{13}C NMR spectrum (126 MHz, $CDCl_3$) of *MP*. The peak corresponding to $CDCl_3$ was truncated for clarity.

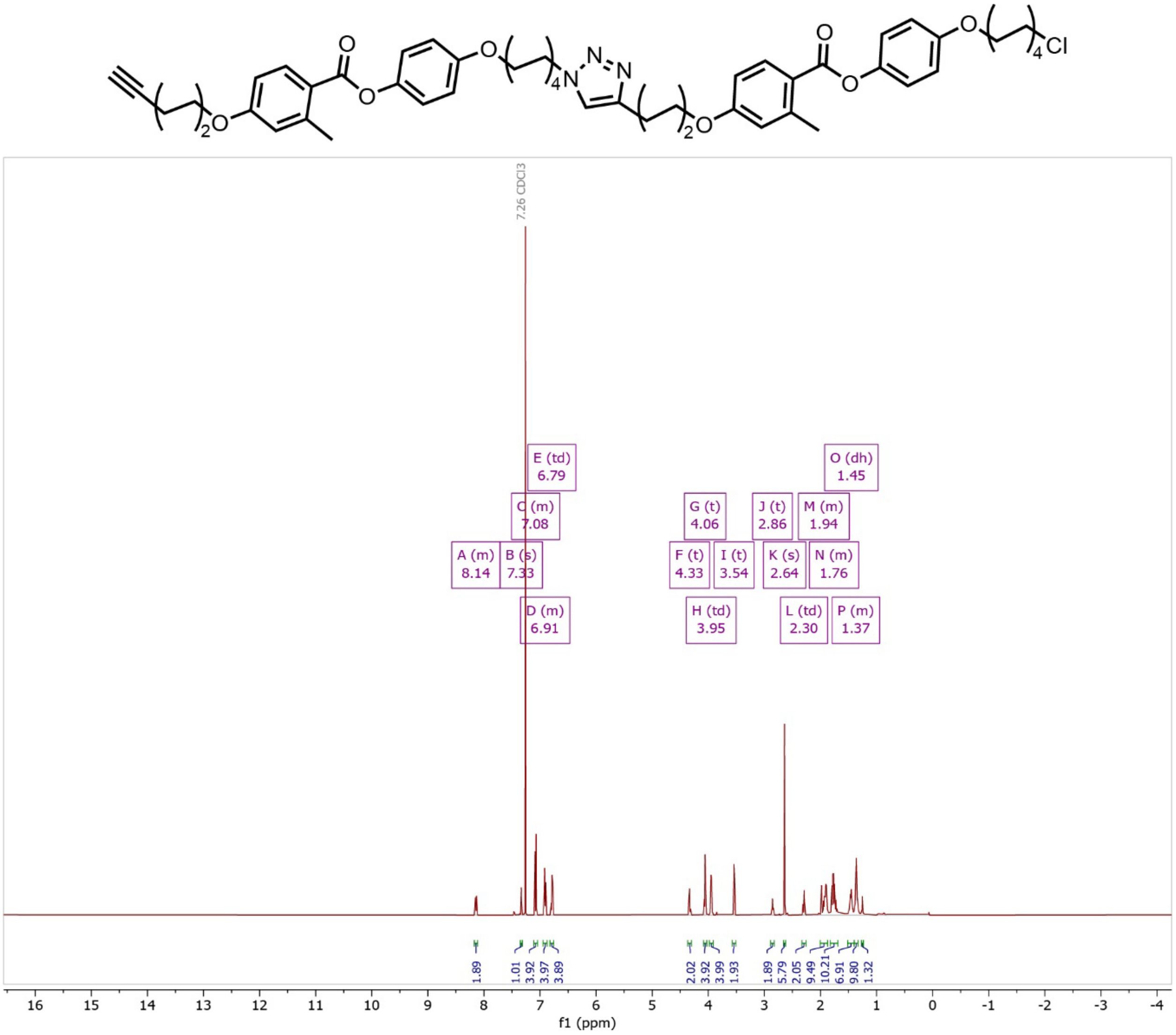

Figure S23: ^{1}H NMR of M_2

^{1}H NMR spectrum (500 MHz, $CDCl_3$) of M_2.

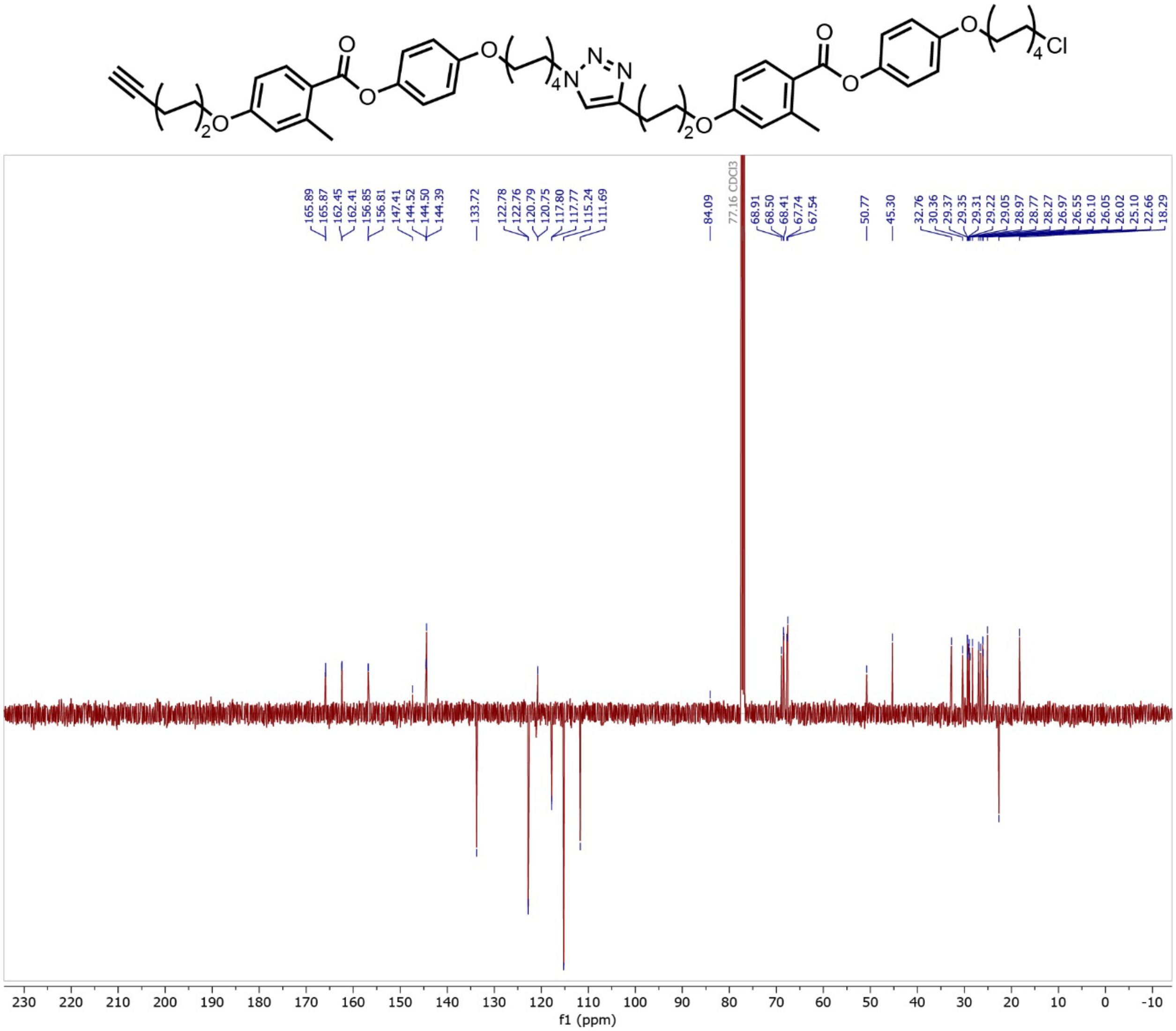

Figure S24: ^{13}C NMR of M_2

^{13}C NMR spectrum (APT, 126 MHz, $CDCl_3$) of M_2. The peak corresponding to $CDCl_3$ was truncated for clarity.

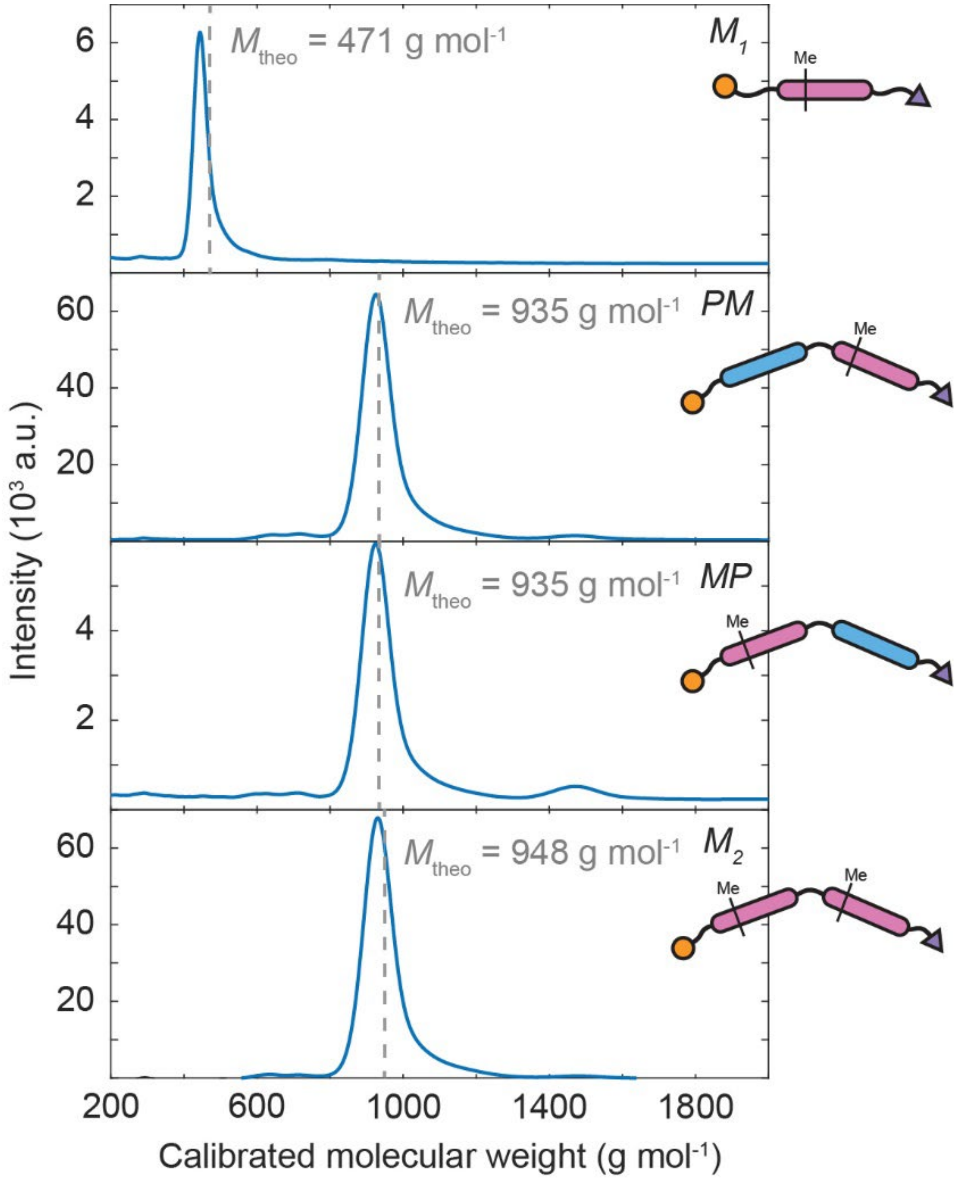

Figure S25: Gel permeation chromatography of M_1, *PM*, *MP*, and M_2

Molecular weight distributions of M_1, *PM*, *MP*, and M_2 calibrated using a fitting function ($\ln M_{peak} = 1.3287 \ln M_{theo} - 1.82303$) previously derived empirically by comparing the molecular weight relative to polystyrene standards (M_{peak}) with the theoretical molecular weight for a series of chemically similar oligomers without methyl substituents (M_{theo}).[1] The dotted lines represent the theoretical molecular weight values.

Section S3: Additional considerations for quantum chemistry calculations and conformational analysis

Bend-angle geometries were analyzed from conformational ensembles generated using density functional theory calculations and a one-dimensional rotational isomeric state (RIS) approximation to evaluate possible odd-even effects. This analysis was intended to isolate intrinsic spacer conformational preferences. In this way, the calculations provide a molecular-level reference for how linker chemistry and parity may influence accessible bend-angle distributions, rather than a direct prediction of the crystalline conformational ensemble. This is because the DFT calculations and RIS treatment do not explicitly include condensed-phase packing, mesogen-mesogen interactions, collective ordering, or correlated conformational changes between neighboring molecules, which may all influence the conformations populated in the crystalline state. We find that triazole-containing spacers access multiple low-energy conformational states that can reduce or obscure a simple odd-even pattern; however, a more definitive description of crystalline-phase conformations would require explicit treatment of packing and intermolecular interactions. This may be pursued in future work through molecular simulation or crystal-structure prediction followed by structural relaxation.

Section S4: Supplementary tables and figures

Dimer	q_{m} (Å^{-1})	d_{m} (nm)	FWHM$_{\mathrm{m}}$ (Å^{-1})	q_{d} (Å^{-1})	d_{d} (nm)	FWHM$_{\mathrm{o}}$ (Å^{-1})
MP	0.2400	2.618	0.0148	0.1174	5.352	0.0130
PM	0.2241	2.804	0.0219	0.1076	5.840	0.0198

Table S1: *q*-values and *d*-spacings corresponding to monomer-scale and dimer-scale order obtained from SAXS after 2 °C min^{-1} cooling from the *Iso* melt.

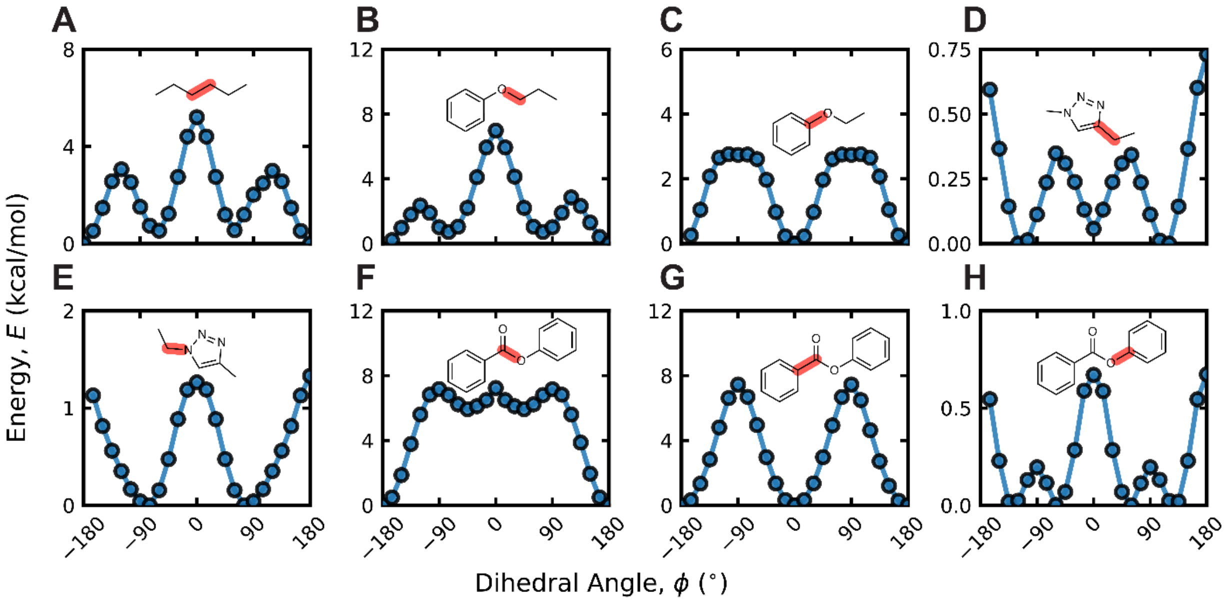

Figure S26: Dihedral scans of selected fragments

A-H) Dihedral mode scans of liquid crystal dimers generated at the ωB97XD/6-31G(d,p) level of theory using the torsiondrive procedure. Chemical structures of the fragments are shown as insets, with the bond being scanned highlighted in red.

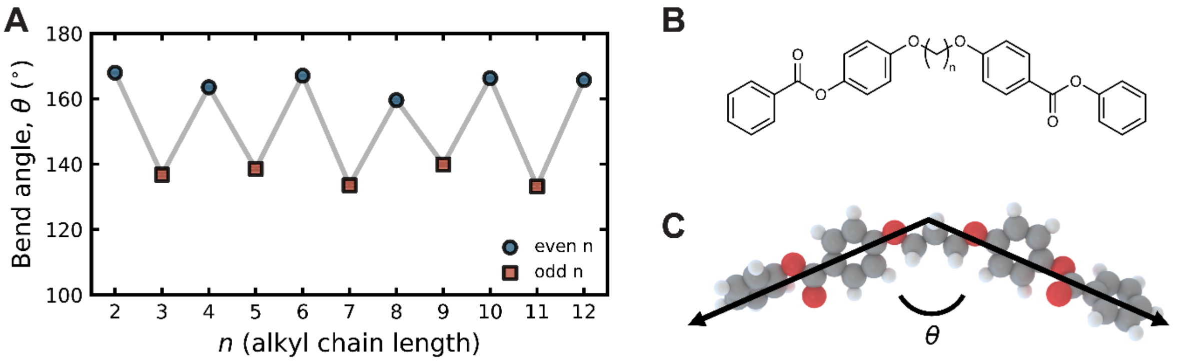

Figure S27: Odd-even effects in alkyl-linked phenylbenzoate dimers

A) Mean bend angle, θ, as a function of alkyl spacer length, n, with even-n homologues shown as blue circles and odd-n homologues as red squares. **B**) Chemical structure of the alkyl-linked phenylbenzoate dimer series with aliphatic tails removed for computational efficiency. **C**) Render illustrating the bend angle for a homologue with 3 carbon linkers at 134°, computed as the arccosine of the dot product of the two mesogen directors, each defined as the vector connecting the centers of mass of the two phenyl rings in a mesogenic core. Renders were generated using OVITO,[2] and the color scheme of the atoms used in the image is as follows: white – H; grey – C; red – O.

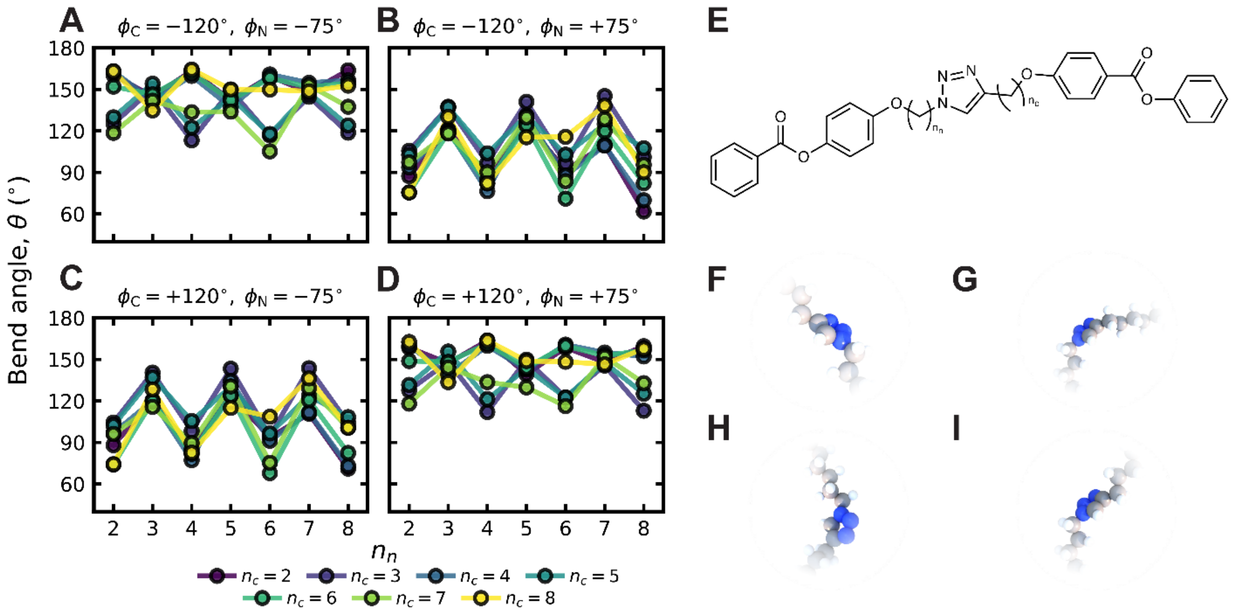

Figure S28: Odd-even effects in triazole-linked phenylbenzoate dimers

A-D) Bend angle of triazole-linked phenylbenzoate dimers as a function of nitrogen-side spacer length (n_n) at different carbon-side spacer lengths (n_c) for the different combinations of triazole rotor minima (ϕ_c and ϕ_n). **E**) Chemical structure of triazole-linked phenylbenzoate dimer series with aliphatic tails removed for computational efficiency. **F-I**) Renders of the lowest-energy conformers at each of the four ϕ_c and ϕ_n rotamer combinations shown in **A-D**, respectively. Renders were generated with OVITO,[2] and the color scheme of the atoms used in the image is as follows: white – H; grey – C; blue – N.

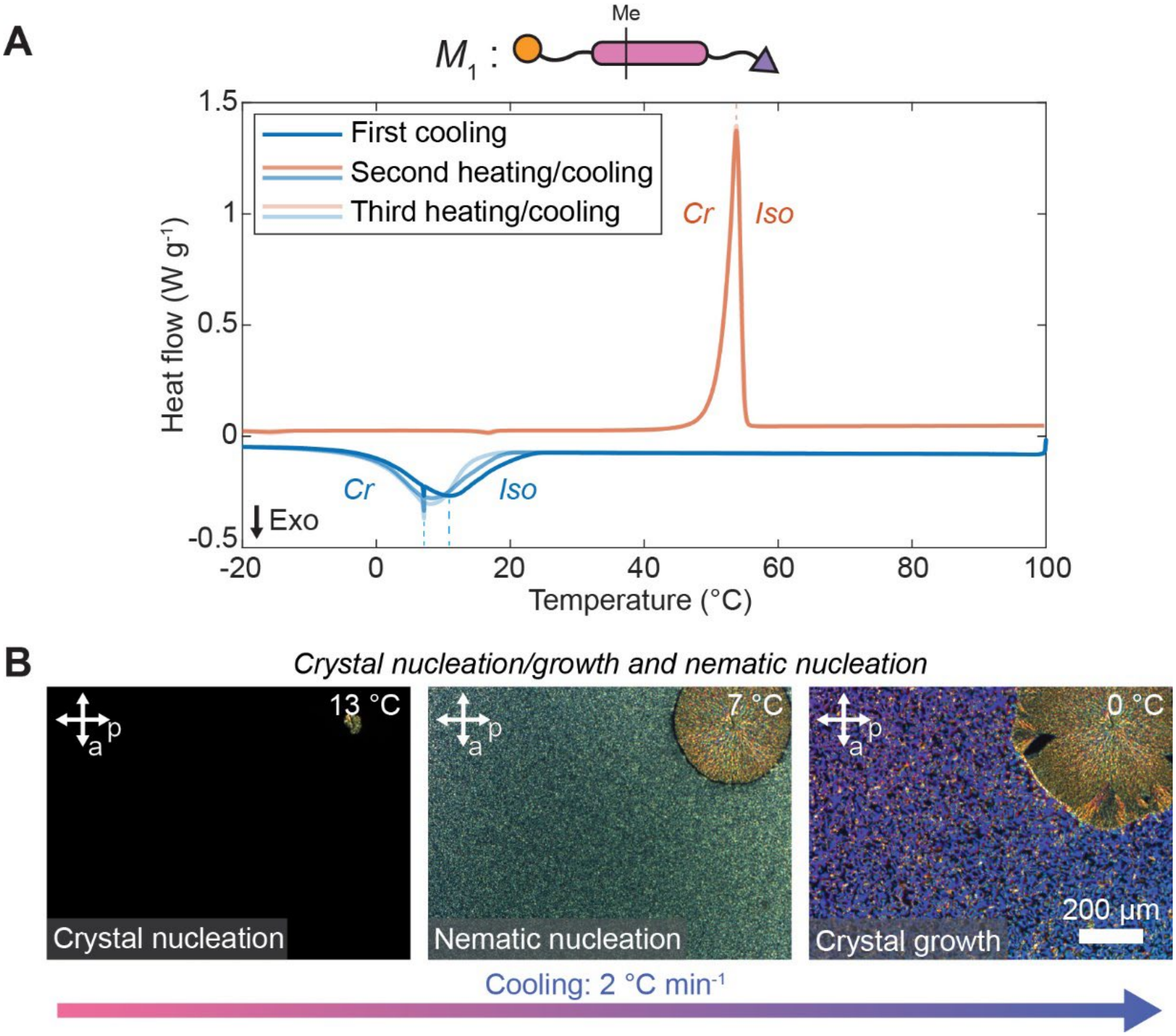

Figure S29: Phase transitions of M_1 during 2 °C min^{-1} cooling

A) DSC traces of M_1 during three heating/cooling cycles at 2 °C min^{-1} showing crystallization overlaid with the *Iso*-*N* transition on cooling followed by melting of the crystalline phase on heating. **B**) Series of micrographs recorded between crossed polarizers representing the crystallization and *Iso*-*N* transition of M_1 in a sandwich cell (~5 µm thickness) during 2 °C min^{-1} cooling from the *Iso* melt.

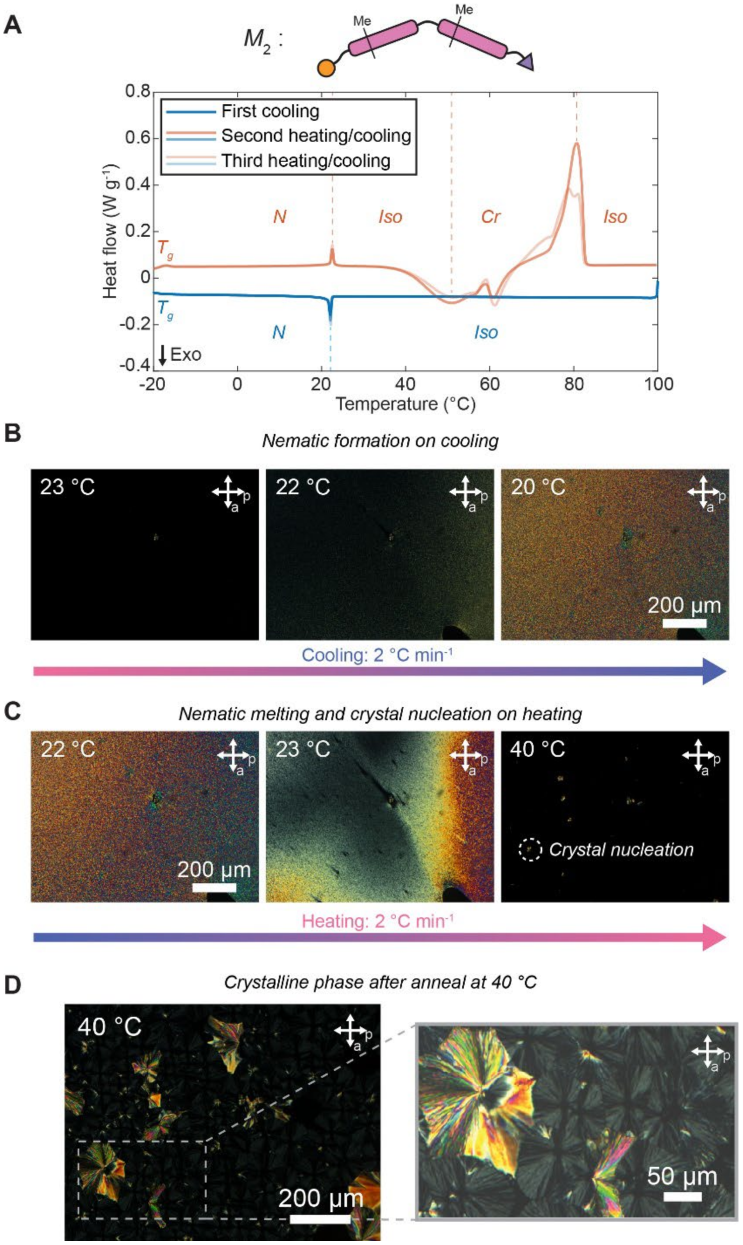

Figure S30: Phase transitions of M_2 during 2 °C min^{-1} cooling and annealing at 40 °C

A) DSC traces of M_2 during three heating/cooling cycles at 2 °C min^{-1} the *Iso*-*N* transition on cooling, then the *Iso*-*N* transition followed by cold crystallization and melting of the crystalline phase on heating. A low-temperature feature possibly corresponding to a glass transition (T_g) of the *N* is labeled on heating and cooling. **B-C**) Series of micrographs recorded between crossed polarizers representing **B**) the transition from the *Iso* to metastable *N* of M_2 during 2 °C min^{-1} cooling from the *Iso* melt and **C**) the *N*-*Iso* transition followed by crystallization during 2 °C min^{-1} heating to 40 °C (immediately after the cooling ramp in **B**). **D**) Micrographs recorded between crossed polarizers of the crystalline phase of M_2 after annealing at 40 °C (immediately after the heating ramp in **C**). Brightness and contrast have been enhanced for the magnified region (right). All micrographs are recorded using a sandwich cell (~5 μm thickness).

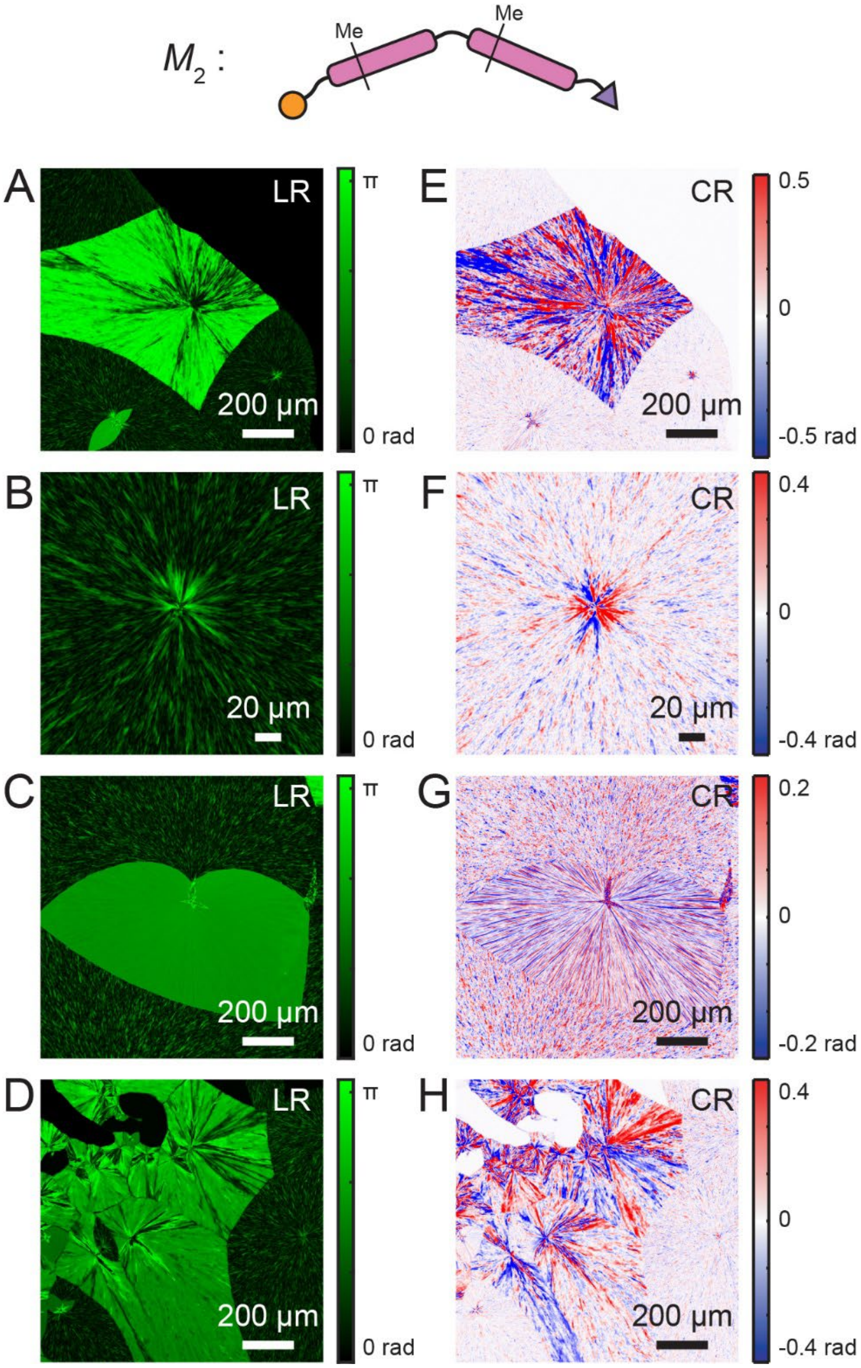

Figure S31: Linear and circular retardance of M_2

A-D) Linear retardance and **E-H**) circular retardance (right) micrographs of a variety of spherulite types formed in the crystalline phase of M_2 after cooling at a rate of 2 °C min^{-1} from the *Iso* melt to 20 °C, directly followed by heating at a rate of 2 °C min^{-1} to 40 °C and an isothermal hold at 40 °C.

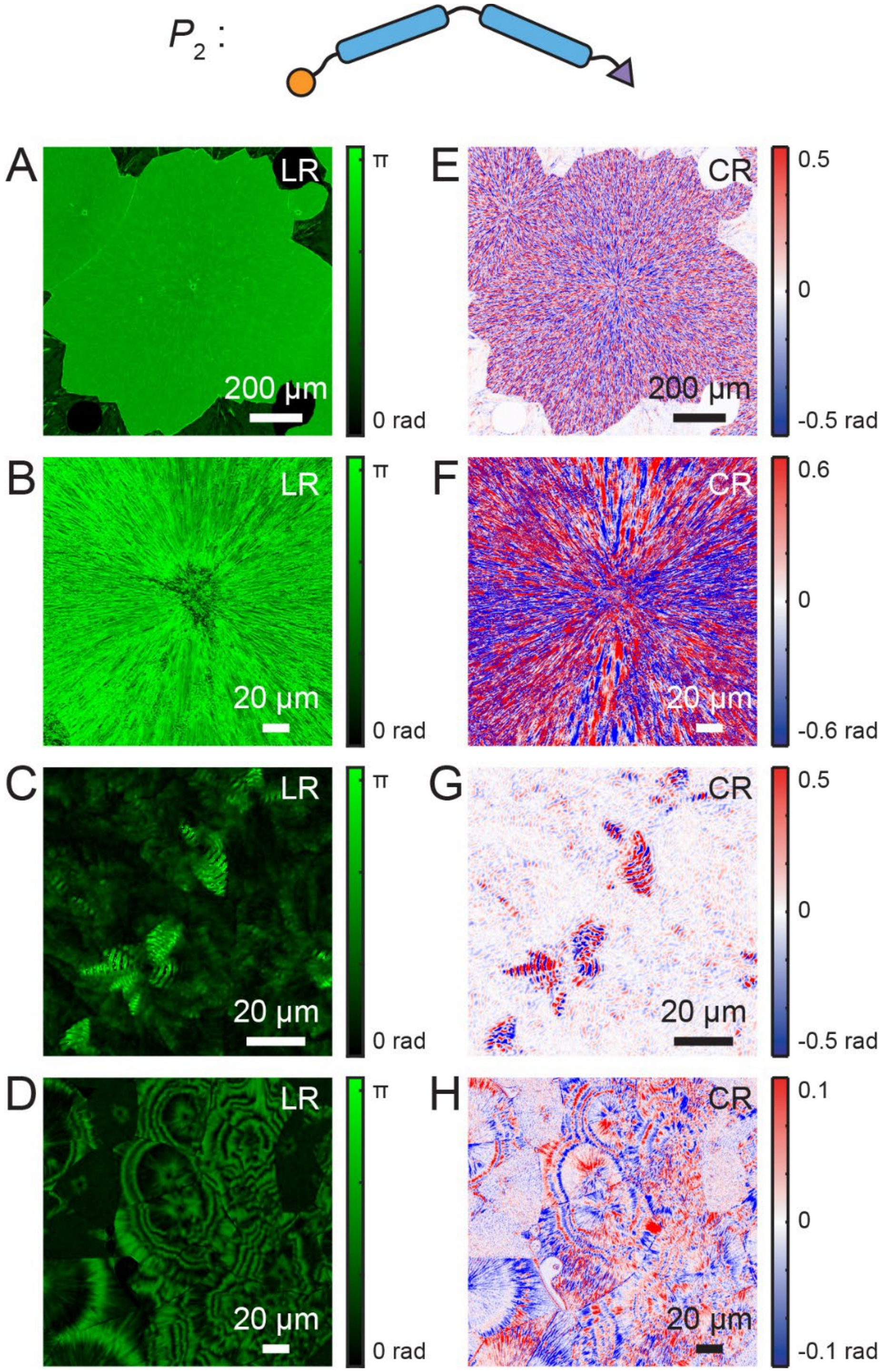

Figure S32: Linear and circular retardance of P_2

A-D) Linear retardance and **E-H**) circular retardance micrographs of P_2. The spherulites in **A-B** and **E-F** were cooled from the *Iso* melt at 2 °C min^{-1}. The periodic banded spherulites in **C** and **G** and the aperiodic banded spherulites in **D** and **H** are formed by isothermal crystallization at 52 °C.

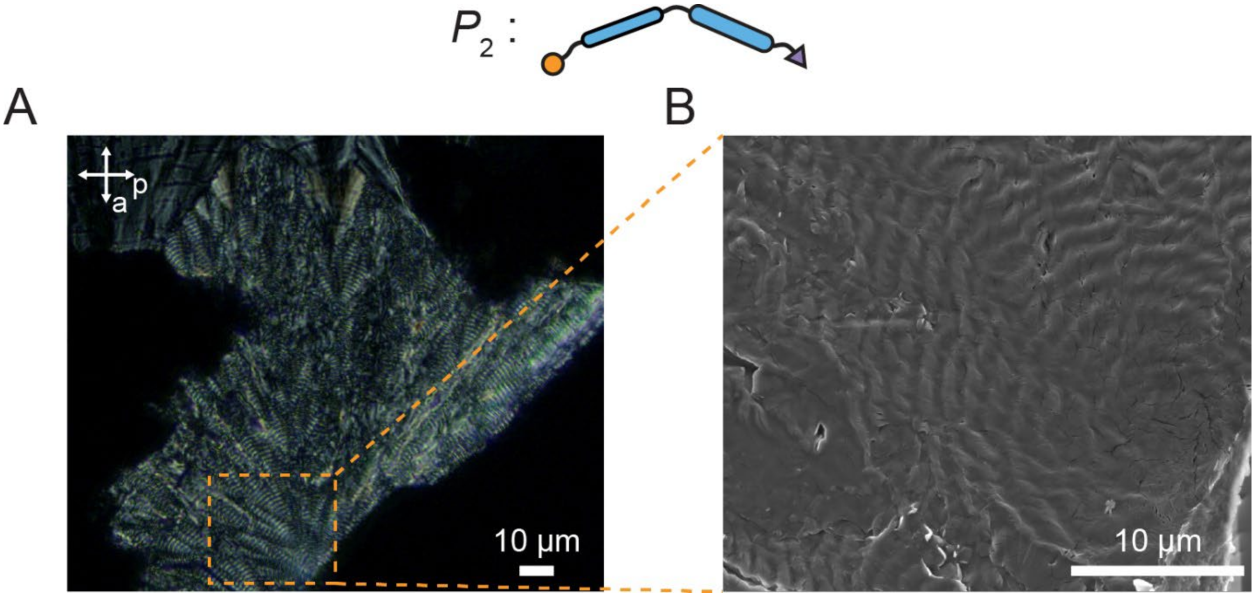

Figure S33: Twisted ribbon microstructure in P_2

A) Polarized optical micrograph showing the banded optical texture and **B**) scanning electron micrograph showing the twisted ribbon microstructure of a banded spherulite formed by P_2 during cooling at a rate of 2 °C min^{-1} from the isothermal melt. The sample was freeze fractured prior to imaging. While limited growth of chiral microstructures can occur in P_2, these morphologies correspond to a different mode of smectic layer curvature (twist) from the preferred smectic layer curvature (bend) of these dimers.

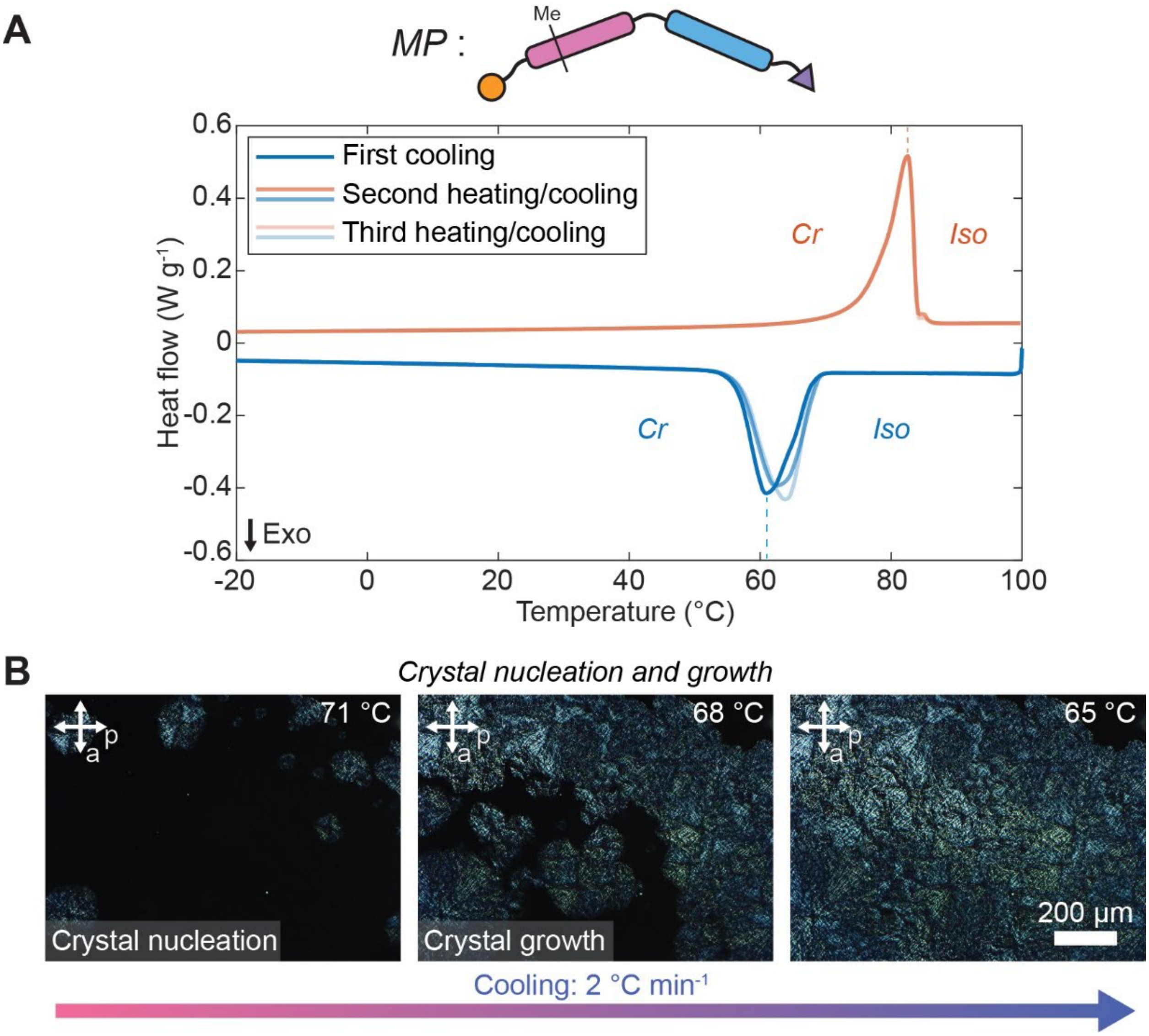

Figure S34: Phase transitions of *MP* during 2 °C min^{-1} cooling

A) DSC traces of *MP* during three heating/cooling cycles at 2 °C min^{-1} showing crystallization on cooling followed by melting on heating. **B**) Series of micrographs recorded between crossed polarizers representing the crystallization of *MP* in a sandwich cell (~5 µm thickness) during 2 °C min^{-1} cooling from the *Iso* melt. Due to the low linear retardance, the brightness and contrast have been adjusted for improved visibility.

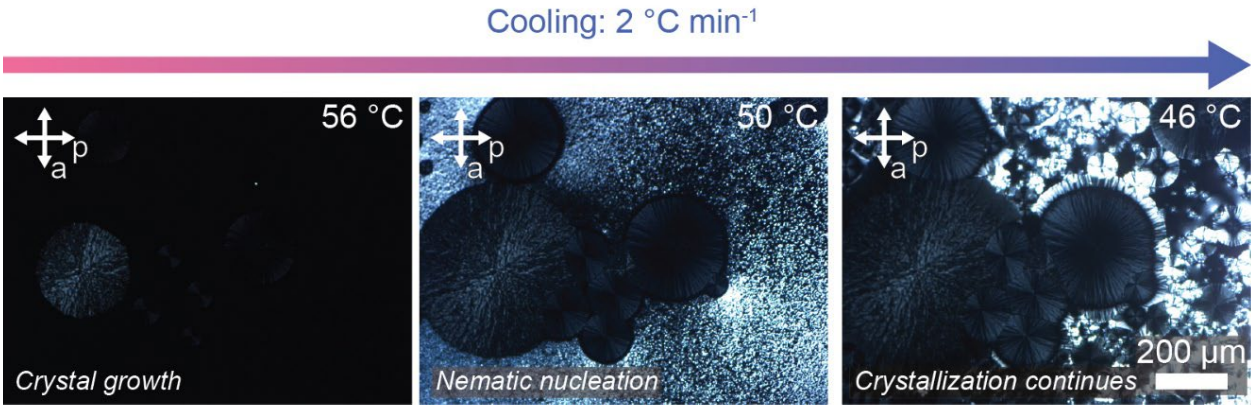

Figure S35: *Iso-N* transition of *MP* during 2 °C min^{-1} cooling

Series of micrographs recorded between crossed polarizers used to determine the T_{ni} of *MP* during 2 °C min^{-1} cooling from the *Iso* melt in a sandwich cell.

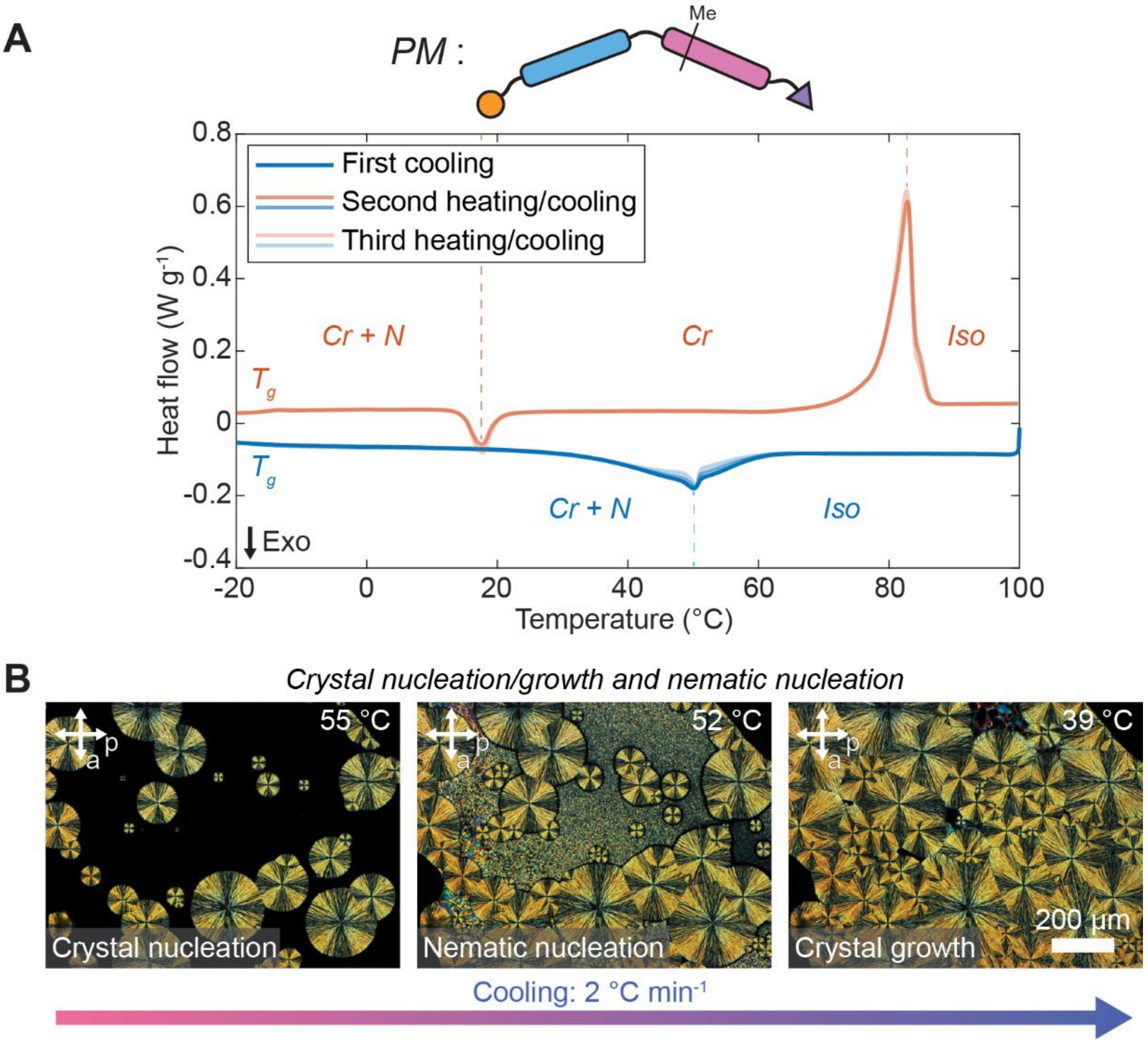

Figure S36: Phase transitions of *PM* during 2 °C min^{-1} cooling

A) DSC traces of *PM* during three heating/cooling cycles at 2 °C min^{-1} showing crystallization overlaid with the *Iso*-*N* transition on cooling, then cold crystallization followed by melting of the crystalline phase on heating. A low-temperature feature possibly corresponding to a glass transition (T_g) of the remaining *N* is labeled on heating and cooling. **B**) Series of micrographs recorded between crossed polarizers representing the crystallization and *Iso*-*N* transition of *PM* in a sandwich cell (~5 µm thickness) during 2 °C min^{-1} cooling from the *Iso* melt.

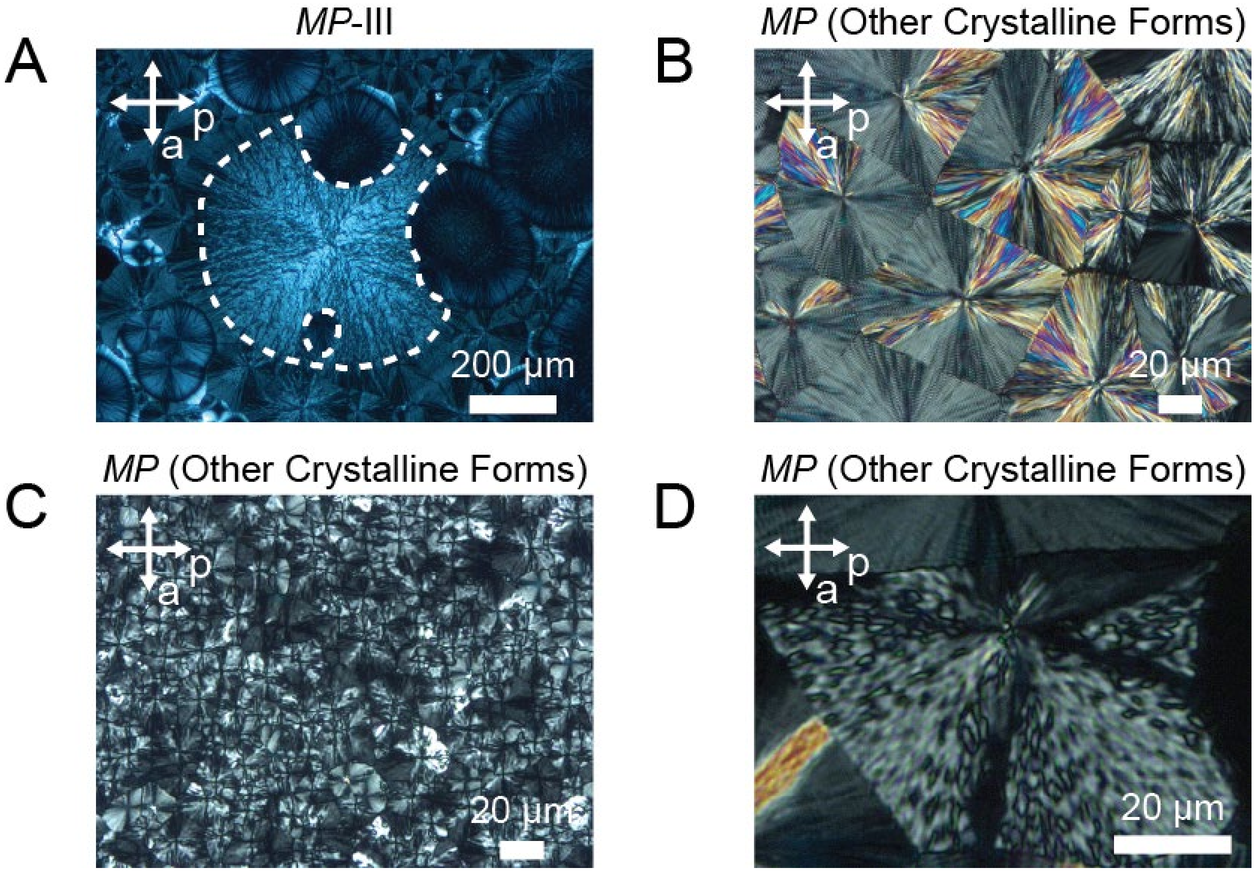

Figure S37: Higher linear retardance crystalline forms exhibited by *MP*

Micrographs recorded between crossed polarizers of **A**) *MP*-III (between the two sets of white dotted lines) and **B-D**) the multiple spherulite types observed in other *MP* crystalline forms. In **A**, the sample was sandwiched between indium tin oxide coated glass (uncontrolled thickness) and cooled 2 °C min^{-1} from the *Iso* melt. The gamma value was 0.45 rather than the typical value of 1. In **B-D**, the sample was isothermally crystallized at 39 °C in a sandwich cell (~5 µm thickness).

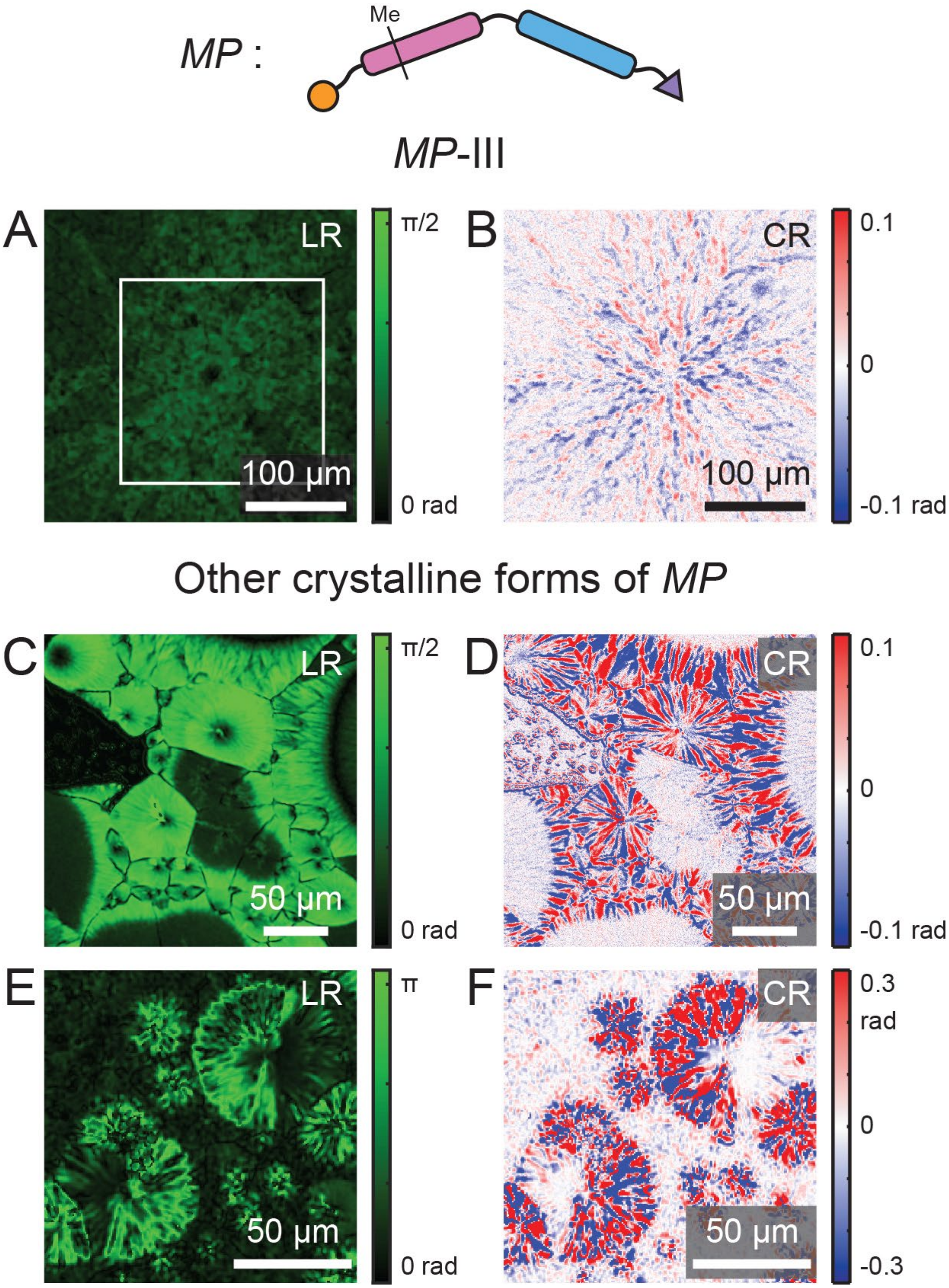

Figure S38: Linear and circular retardance of *MP*-III and other *MP* crystalline forms

A) Linear retardance (LR) and **B**) circular retardance (CR) micrographs of *MP*-III. **C-F**) Micrographs of the multiple spherulite types observed in the other *MP* crystalline forms showing LR (**C**, **E**) and CR (**D**, **F**). The area used to calculate the average LR for *MP*-III is shown by the white box in **A**. The sample was cooled at a rate of 2 °C min^{-1} from the *Iso* melt.

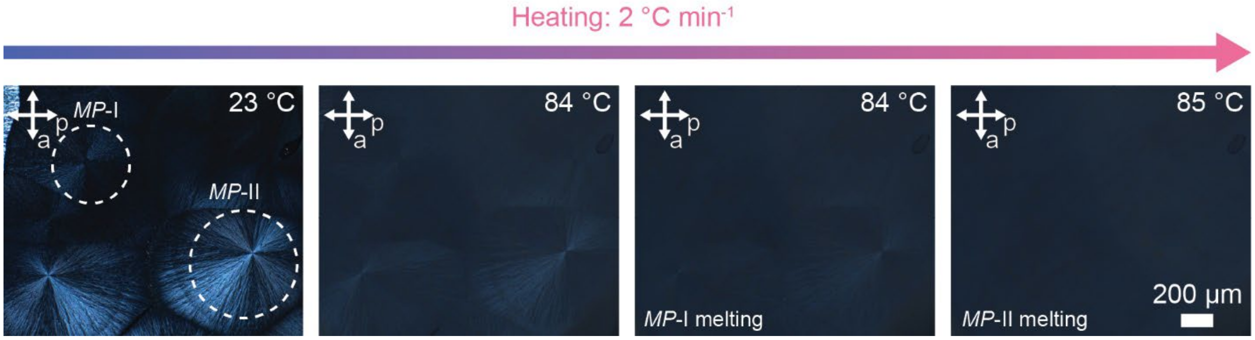

Figure S39: Melting of *MP*-I and *MP*-II during 2 °C min^{-1} heating

Micrographs recorded between crossed polarizers representing the similar melting temperatures of *MP*-I and *MP*-II during 2 °C min^{-1} heating from room temperature in a sandwich cell (~5 µm thickness). Before the heating ramp shown here, the sample had been cooled 2 °C min^{-1} from the *Iso* melt. Upon heating, *MP*-I melts first at 84 °C, followed by *MP*-II at 85 °C. Due to the low linear retardance near the melting point, brightness and contrast have been adjusted for the micrographs at 84 °C and 85 °C for increased visibility of the spherulites.

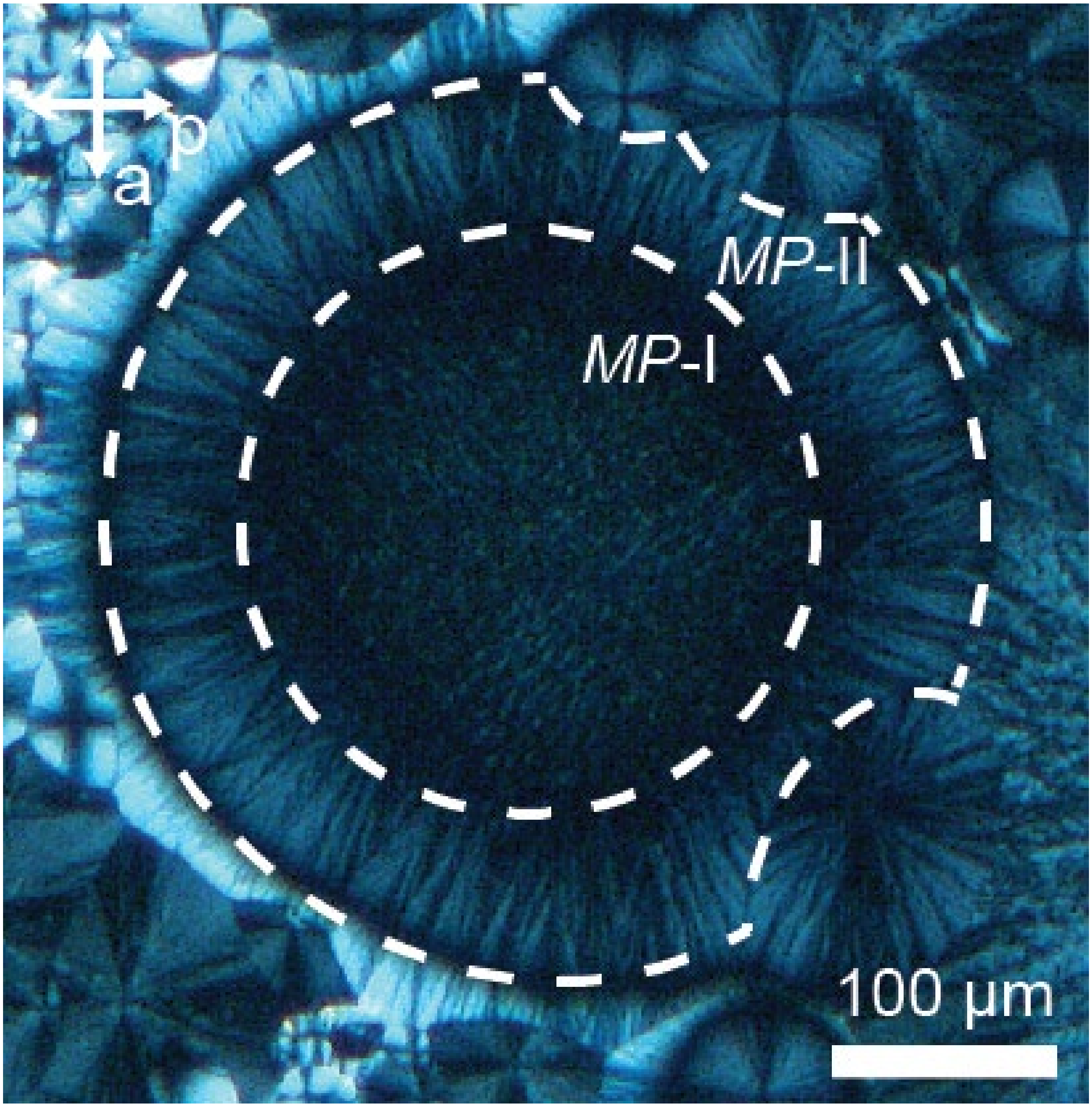

Figure S40: Spherulite with *MP*-I core and *MP*-II outer ring.

Micrographs recorded between crossed polarizers of *MP* following 2 °C min^{-1} from the *Iso* melt. The lower LB inner region of the largest spherulite in the center is the *MP*-I crystalline form (surrounded by the innermost white dotted circle), and a higher LB ring of *MP*-II surrounds it (between the two sets of white dotted lines). This spherulite is surrounded by smaller spherulites of *MP*-II, *MP*-III, and other *MP* crystalline forms. Unlike other sandwich cells, this sample was sandwiched between indium tin oxide-coated glass (uncontrolled thickness). The gamma value during imaging was 0.45 rather than the typical value of 1.

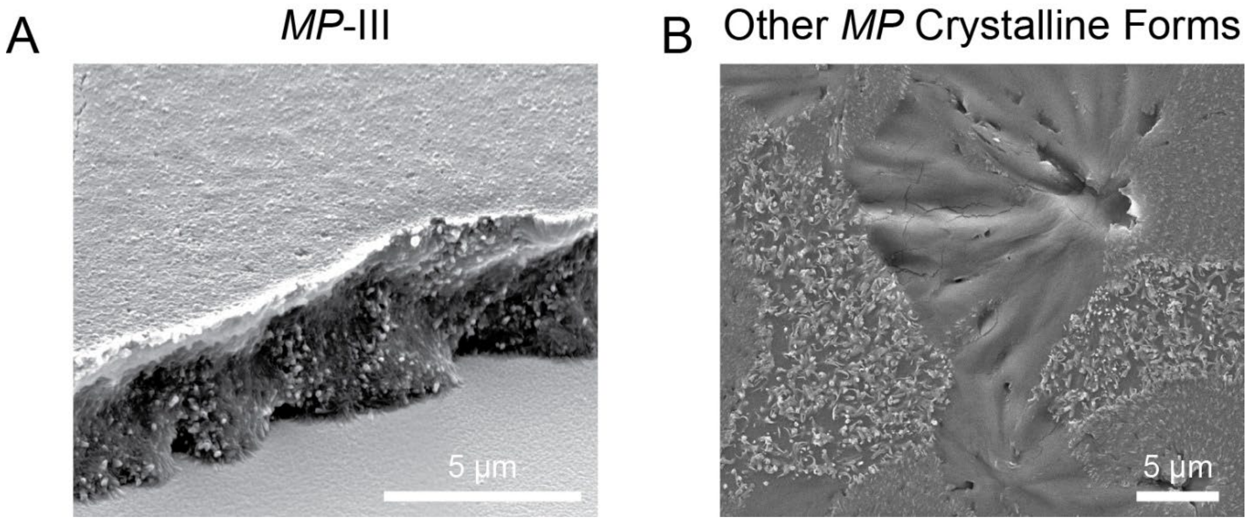

Figure S41: Scanning electron micrographs of *MP*-III and other *MP* crystalline forms

Scanning electron micrograph of **A**) a cross section of a *MP*-III spherulite collected at a 54° tilt with respect to the detector and **B**) a top-down view of multiple spherulite types included in the other *MP* crystalline forms. The sample was prepared by freeze fracturing of a sandwich cell using indium tin oxide-coated glass (uncontrolled thickness) after cooling 2 °C min^{-1} from the *Iso* melt.

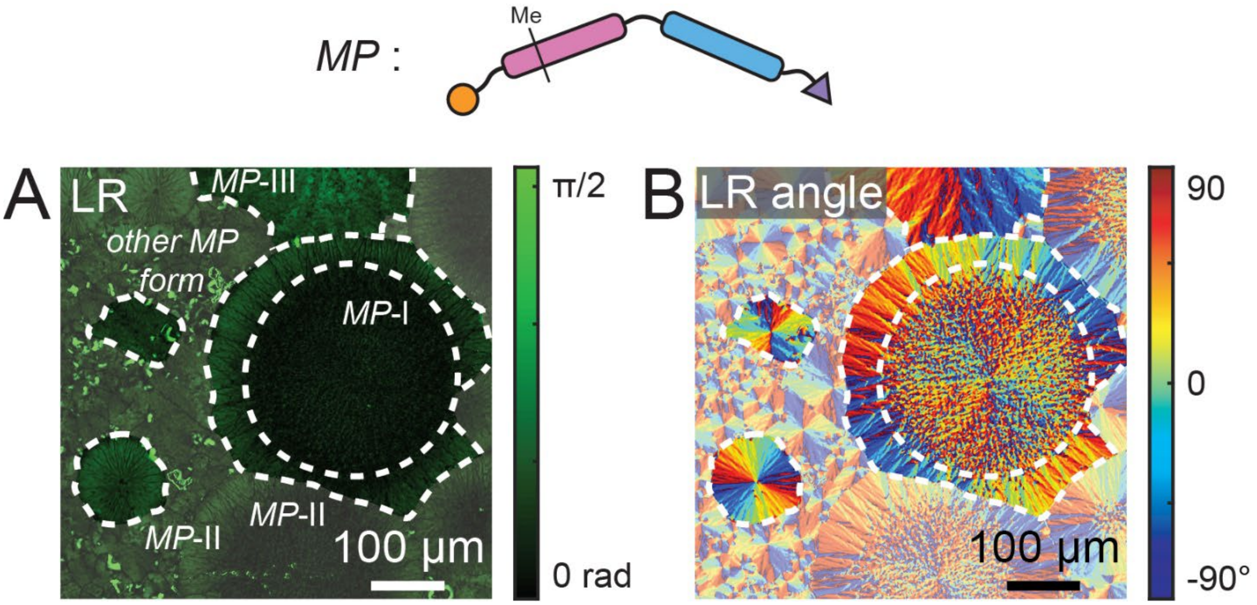

Figure S42: Linear retardance azimuthal orientation of *MP*

A) Linear retardance (LR) and **B**) LR azimuthal orientation micrographs of *MP* after cooling 2 °C min^{-1} from the *Iso* melt. In **B** the larger refractive index is plotted clockwise from the horizon. Regions corresponding to *MP*-I, *MP*-II, *MP*-III, and one other *MP* form are indicated by dotted white lines (with labels shown in **A**), and all other regions are faded for clarity. *MP*-I, *MP*-III, and certain other *MP* forms are negative spherulites, while *MP*-II crystallizes into positive spherulites.

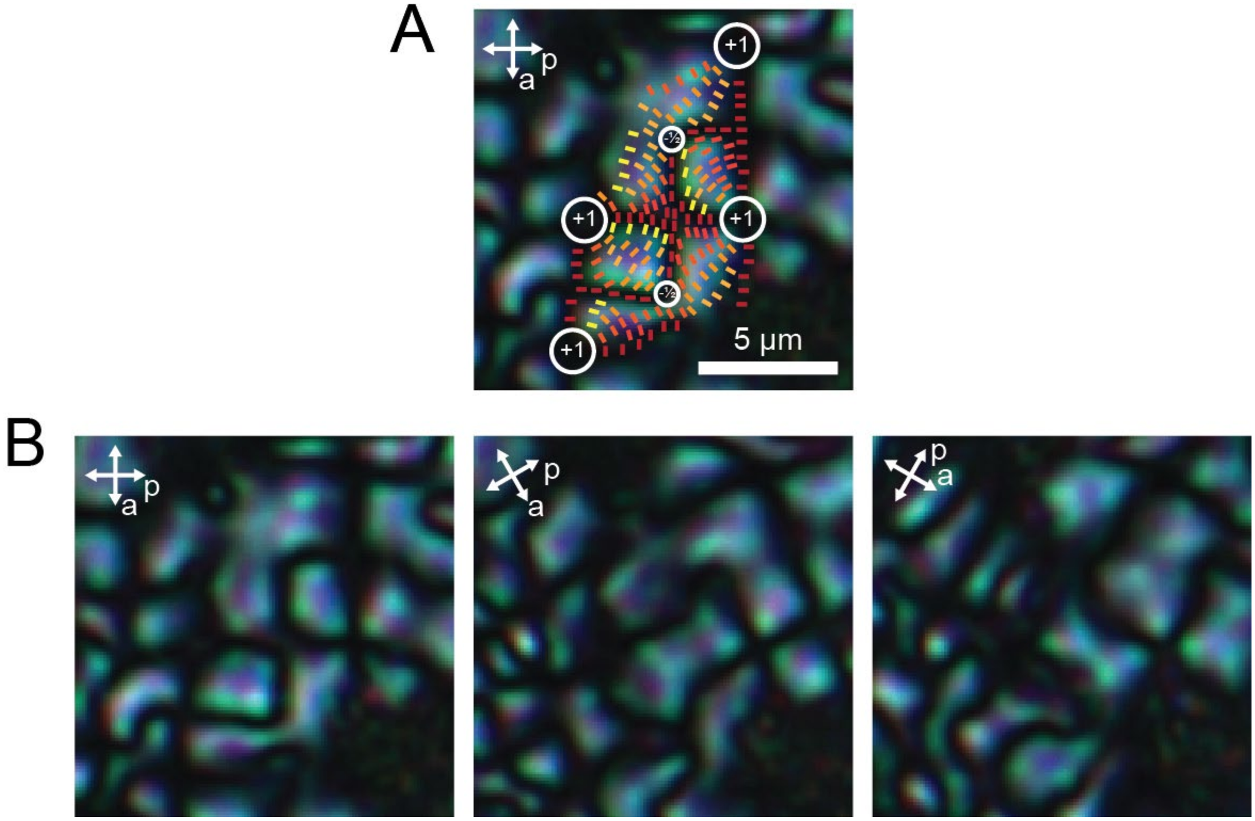

Figure S43: Defect pattern of *PM*-II under crossed polarizers

A) Micrograph recorded between crossed polarizers of *PM*-II overlaid with the defect pattern, where each line represents the orientation of the extraordinary refractive index of the material. **B**) Series of micrographs of the region of *PM*-II shown in **A** between crossed polarizers, after 30° rotation, and after 60° rotation of the crossed polarizers. The spherulites do not exhibit domain boundaries that remain fixed upon rotation of the polarizers, instead forming dark brushes with right-angle "elbow" shapes that depend on the orientation of the crossed polarizers. The sample was prepared by cooling at a rate of 2 °C min^{-1} from the *Iso* melt in a sandwich cell (~5 µm thickness).

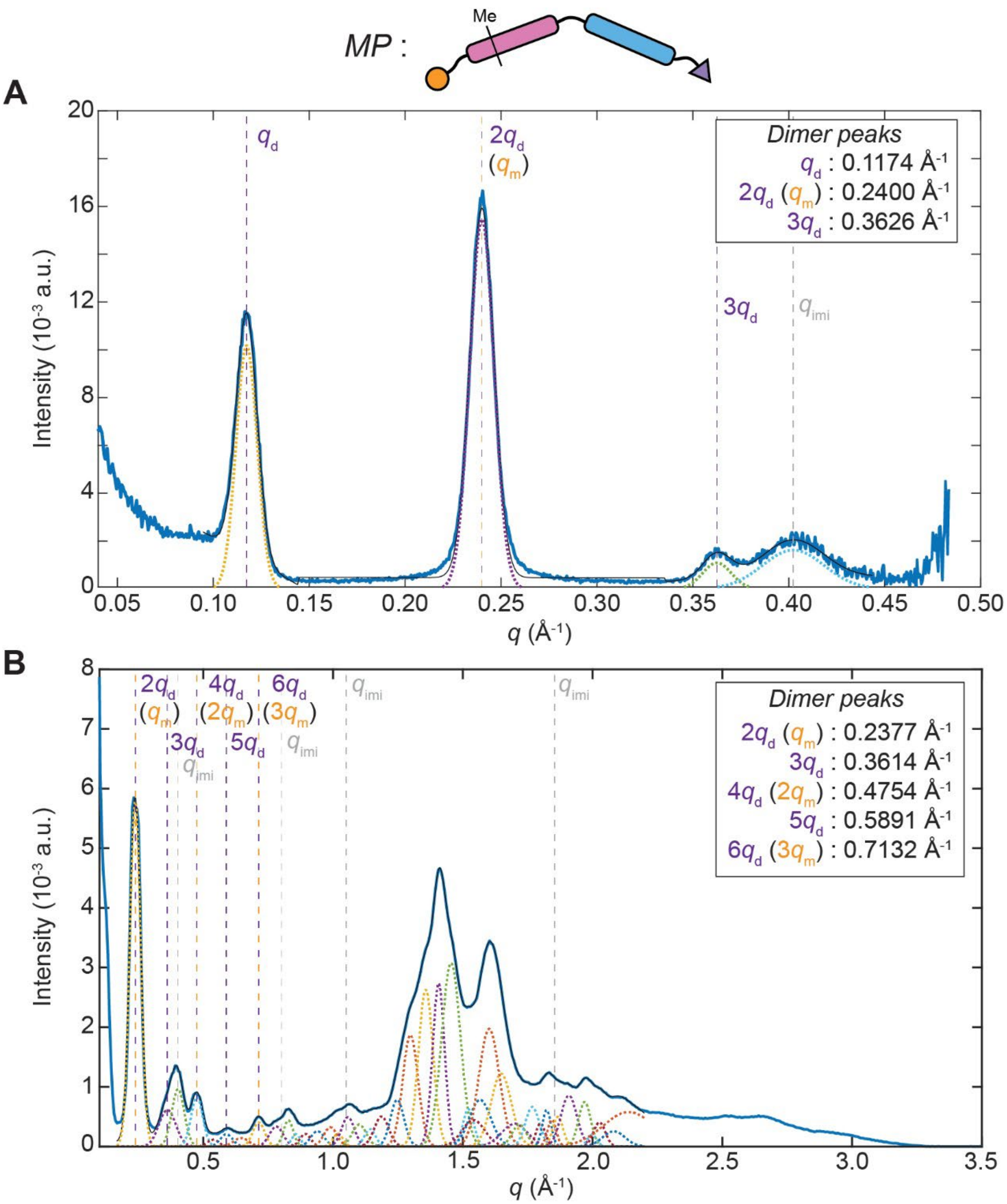

Figure S44: X-ray scattering spectrum and peak fitting for bulk *MP*

Azimuthally integrated 1D X-ray scattering patterns of a bulk *MP* sample (uncontrolled mixture of *MP*-I, *MP*-II, and *MP*-III) with the detector in the **A**) SAXS and **B**) WAXS position. The measurement was taken at room temperature following isothermal crystallization at 67 °C. The dotted lines represent each Gaussian curve, which each contribute to the fitted curve shown in black. The positions of the peaks associated with the length of the monomer (q_m) and dimer (q_d) are shown in orange and purple, respectively. The positions of the peaks associated with the polyimide substrate are shown in grey.

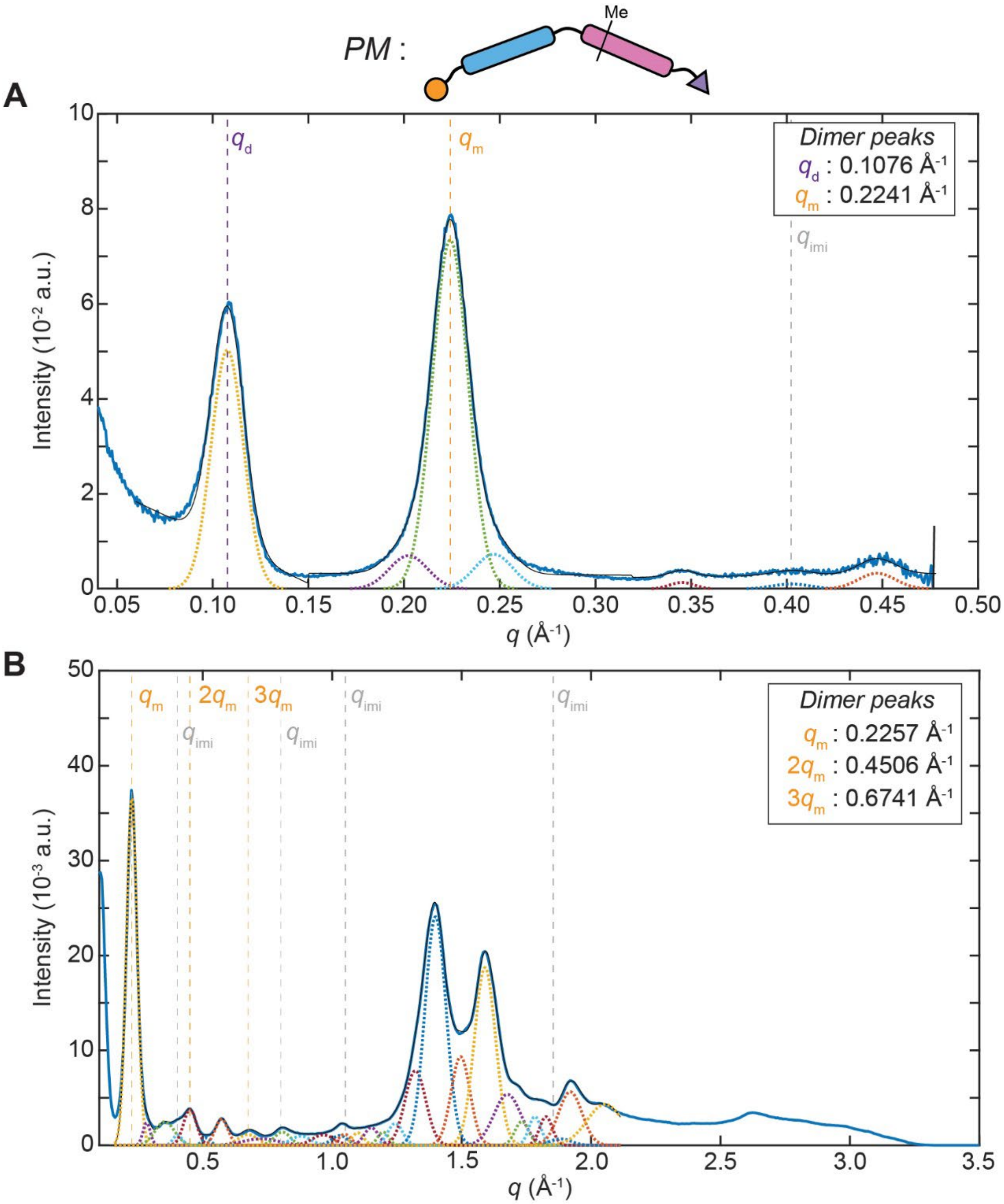

Figure S45: X-ray scattering spectrum and peak fitting for bulk *PM*

Azimuthally integrated 1D X-ray scattering patterns of a bulk *PM* sample (expected to be primarily composed of *PM*-I) with the detector in the **A**) SAXS and **B**) WAXS position. The measurement was taken at room temperature following isothermal crystallization at 58 °C. The dotted lines represent each Gaussian curve, which each contribute to the fitted curve shown in black. The positions of the peaks associated with the length of the monomer (q_m) and dimer (q_d) are shown in orange and purple, respectively. The positions of the peaks associated with the polyimide substrate are shown in grey.

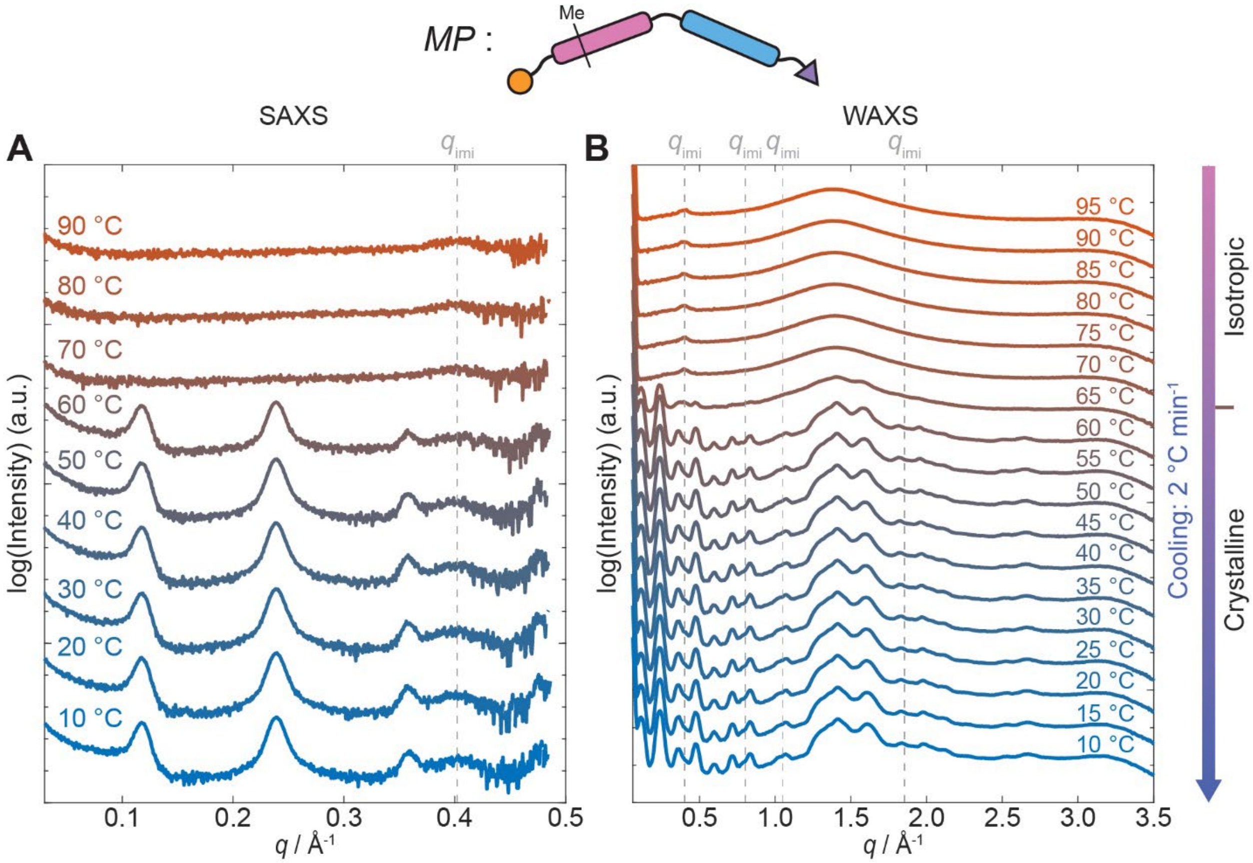

Figure S46: Temperature-dependent X-ray scattering spectrum for bulk *MP*

Azimuthally integrated 1D X-ray scattering patterns of a bulk *MP* sample with the detector in the **A**) SAXS and **B**) WAXS position. The *in-situ* measurement was collected during cooling at 2 °C min^{-1}. The positions of the peaks associated with the polyimide substrate are shown in grey.

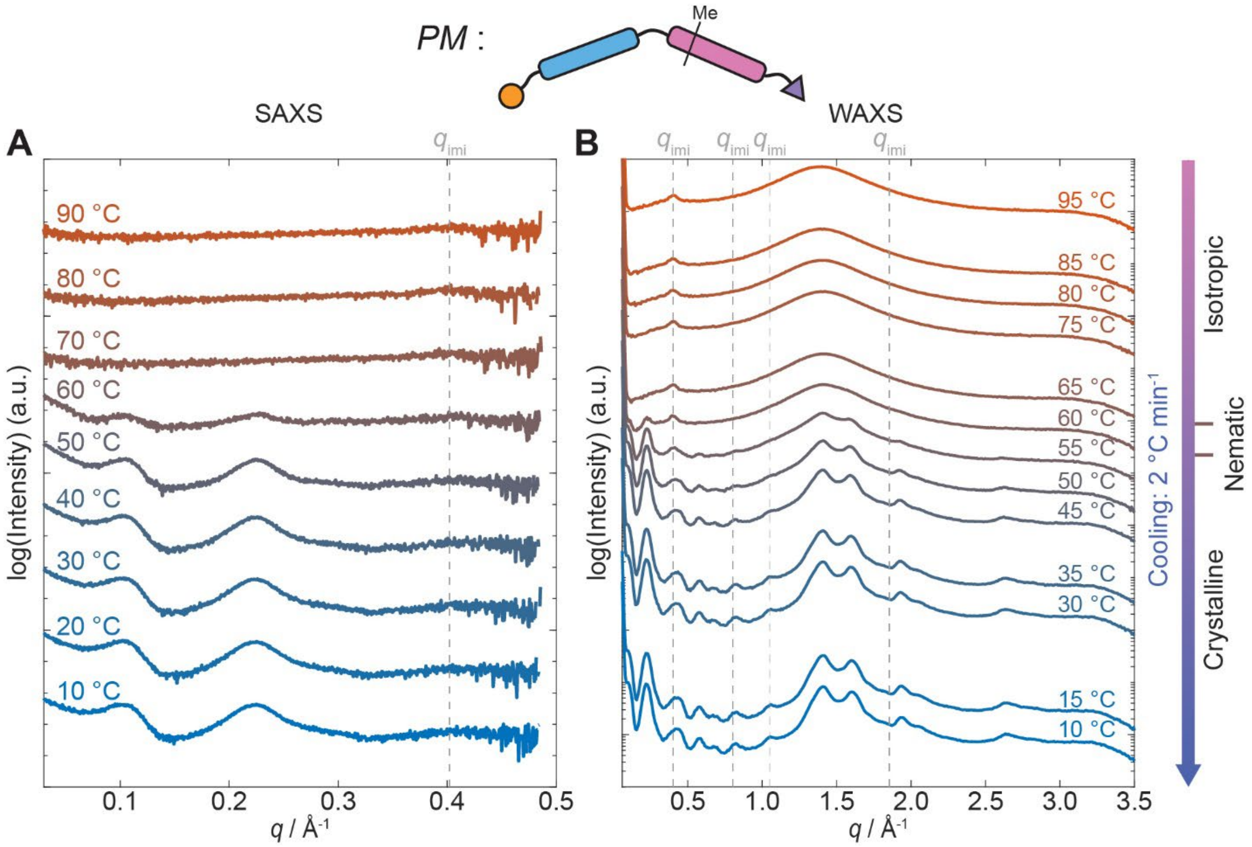

Figure S47: Temperature-dependent X-ray scattering spectrum for bulk *PM*

Azimuthally integrated 1D X-ray scattering patterns of a bulk *PM* sample with the detector in the **A**) SAXS and **B**) WAXS position. The *in-situ* measurement was collected during cooling at 2 °C min^{-1}. The positions of the peaks associated with the polyimide substrate are shown in grey.

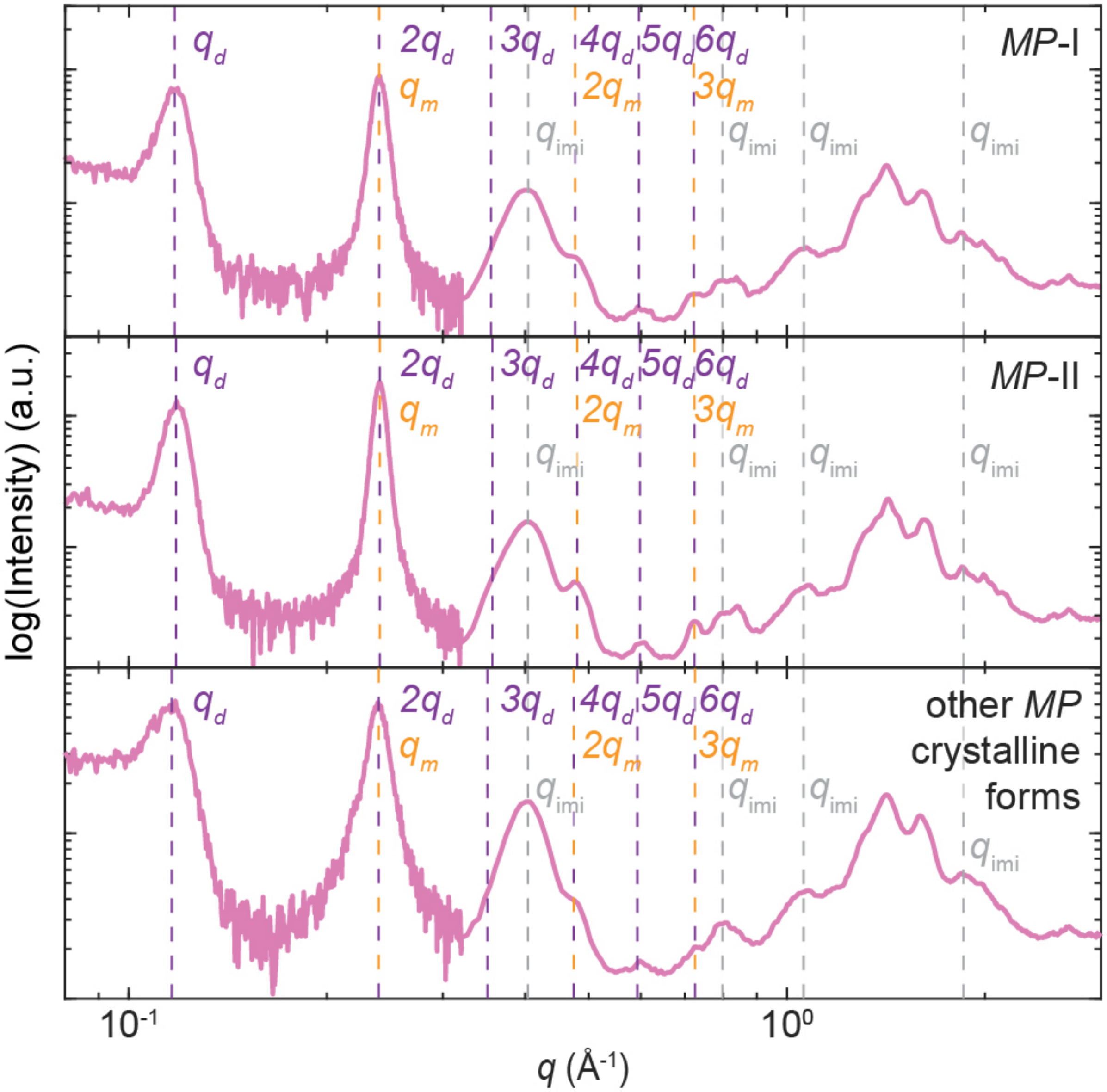

Figure S48: X-ray scattering spectra of *MP*-I, *MP*-II, and other *MP* crystalline forms

Azimuthally integrated 1D X-ray scattering patterns of *MP*-I (top), *MP*-II (middle), and *MP*'s other crystalline forms (bottom). The spectra consist of two separate measurements with the detector in the SAXS and WAXS positions. The measurement was taken at room temperature following isothermal crystallization between polyimide substrates at 67 °C for *MP*-I, 55 °C for *MP*-II, and 39 °C for *MP*'s other crystalline forms, highlighting their similar molecular packing despite disparate long-range order in their microstructures. Although *MP*-I and *MP*-II cannot be fully isolated by isothermal crystallization temperatures, the spherulite type corresponding to the region in which the X-ray measurement was taken was verified by optical microscopy. The positions of the peaks associated with the length of the monomer (q_m) and dimer (q_d) are shown in orange and purple, respectively. The positions of the peaks associated with the polyimide substrate are shown in grey.

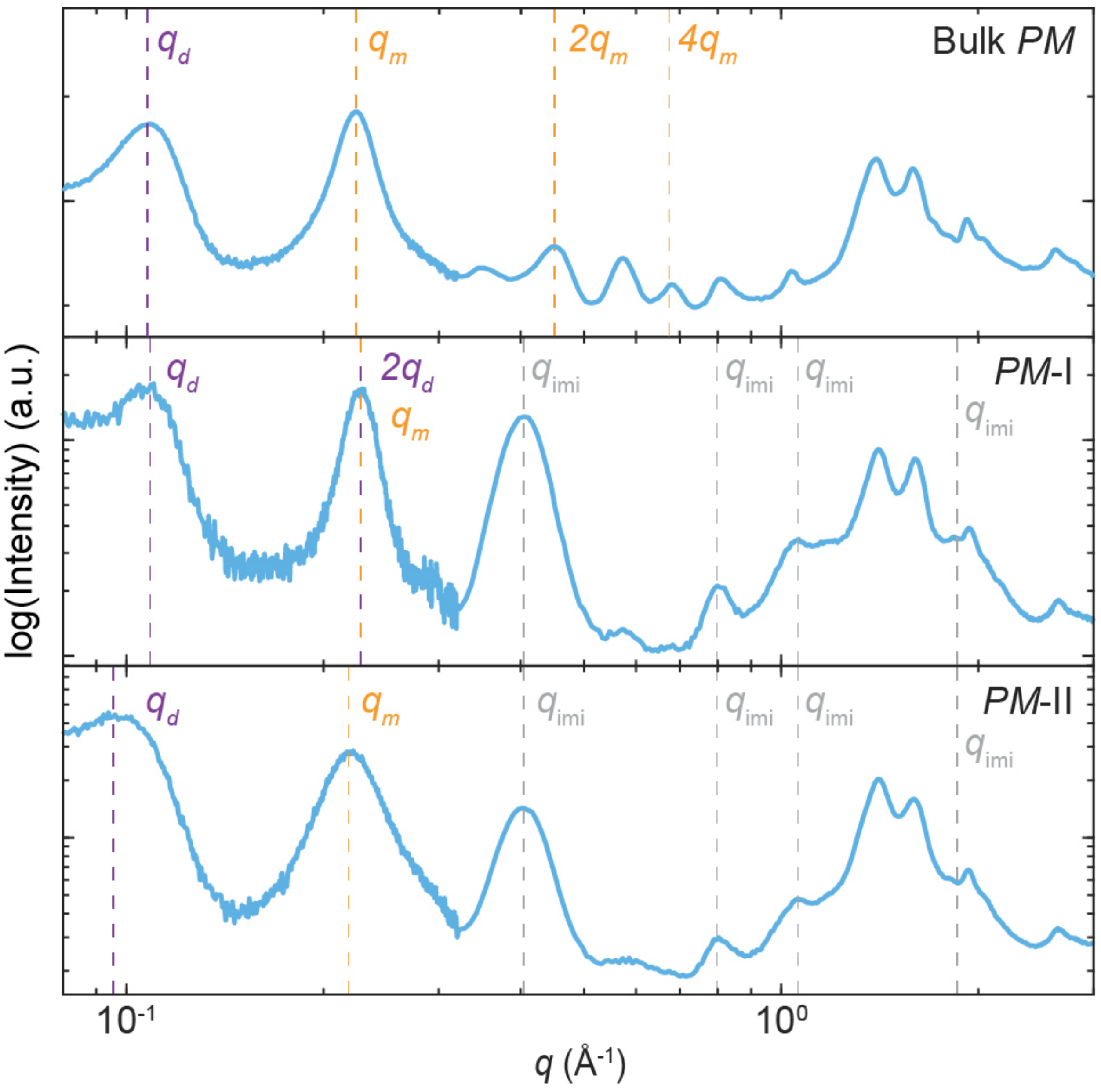

Figure S49: X-ray scattering spectra of bulk *PM*, *PM*-I and *PM*-II

Azimuthally integrated 1D X-ray scattering patterns of bulk *PM* (top), *PM*-I (middle) and *PM*-II (bottom). The bulk *PM* sample is likely primarily composed of *PM*-I due to their similar molecular packing, while in *PM*-II the peak associated with the length of the dimer is shifted to a lower *q*-value. The spectra consist of two separate measurements with the detector in the SAXS and WAXS positions. The measurement was taken at room temperature between polyimide substrates following isothermal crystallization at 58 °C for bulk *PM* and *PM*-I and a quench from the *Iso* melt to room temperature for *PM*-II. The positions of the peaks associated with the length of the monomer (q_m) and dimer (q_d) are shown in orange and purple, respectively. The positions of the peaks associated with the polyimide substrate are shown in grey. The background scattering from the polyimide substrate has been subtracted for bulk *PM*.

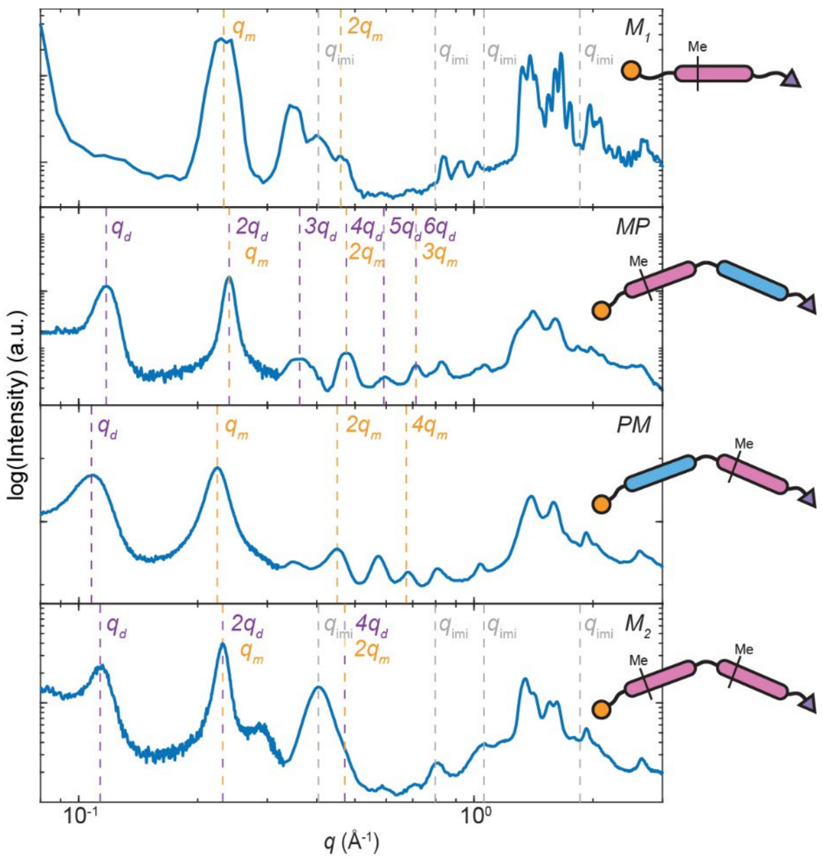

Figure S50: X-ray scattering spectra of monomers and dimers

Azimuthally integrated 1D X-ray scattering patterns of (from top to bottom) monomer M_1 and dimers *MP*, *PM*, and M_2. The spectra consist of two separate measurements with the detector in the SAXS and WAXS positions. The measurement was taken between polyimide substrates during in situ cooling at a rate of 2 °C min^{-1} at 20 °C for M_1. For the other materials, isothermal crystallization was performed at 67 °C for *MP*, 58 °C for *PM*, and 40 °C for M_2. The positions of the peaks associated with the length of the monomer (q_m) and dimer (q_d) are shown in orange and purple, respectively. The positions of the peaks associated with the polyimide substrate are shown in grey. The background scattering from the polyimide substrate has been subtracted for all except M_1 and M_2. The spectra of P_1 and P_2 can be found in a previous publication.[1]

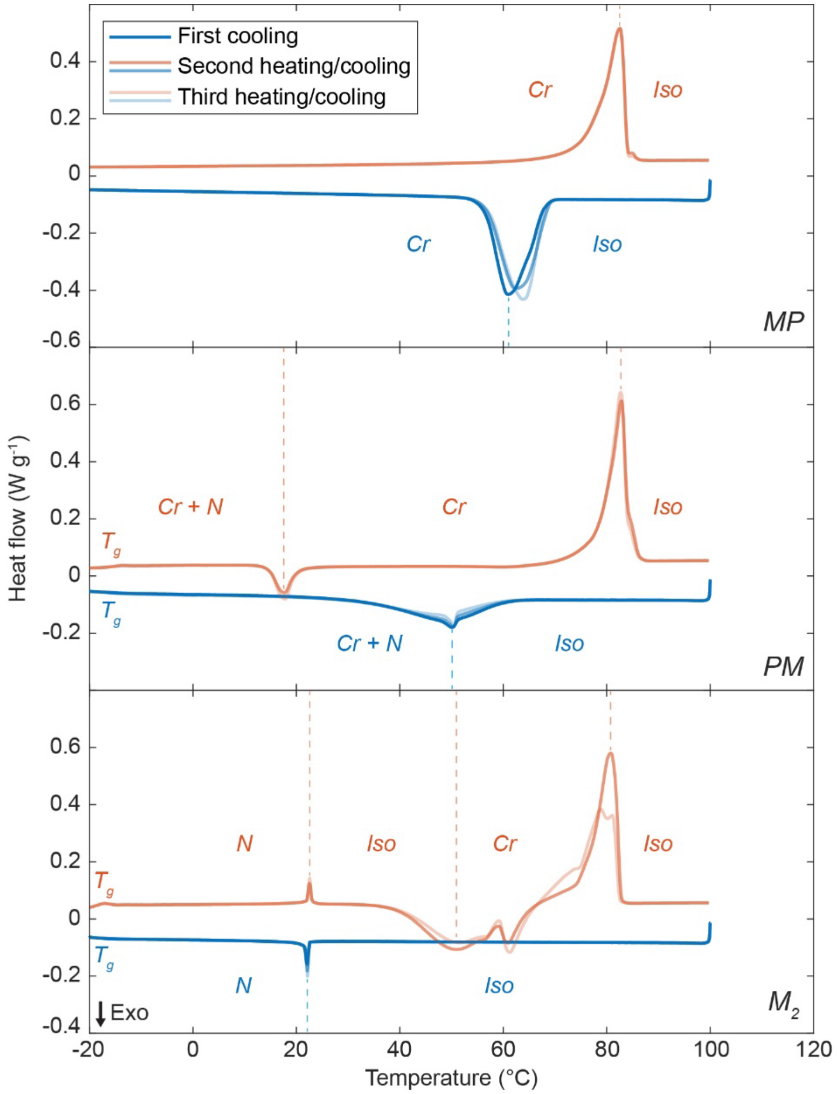

Figure S51: Differential scanning calorimetry of dimers

DSC traces of (from top to bottom) *MP*, *PM*, and M_2 during three heating/cooling cycles at 2 °C min^{-1}. The melting temperature decreases as the number of methyl substituents increases, with P_2 having the highest T_m (DSC of P_2 can be found in a previous publication),[1] the heterodimers having intermediate T_m values, and M_2 having the lowest T_m.

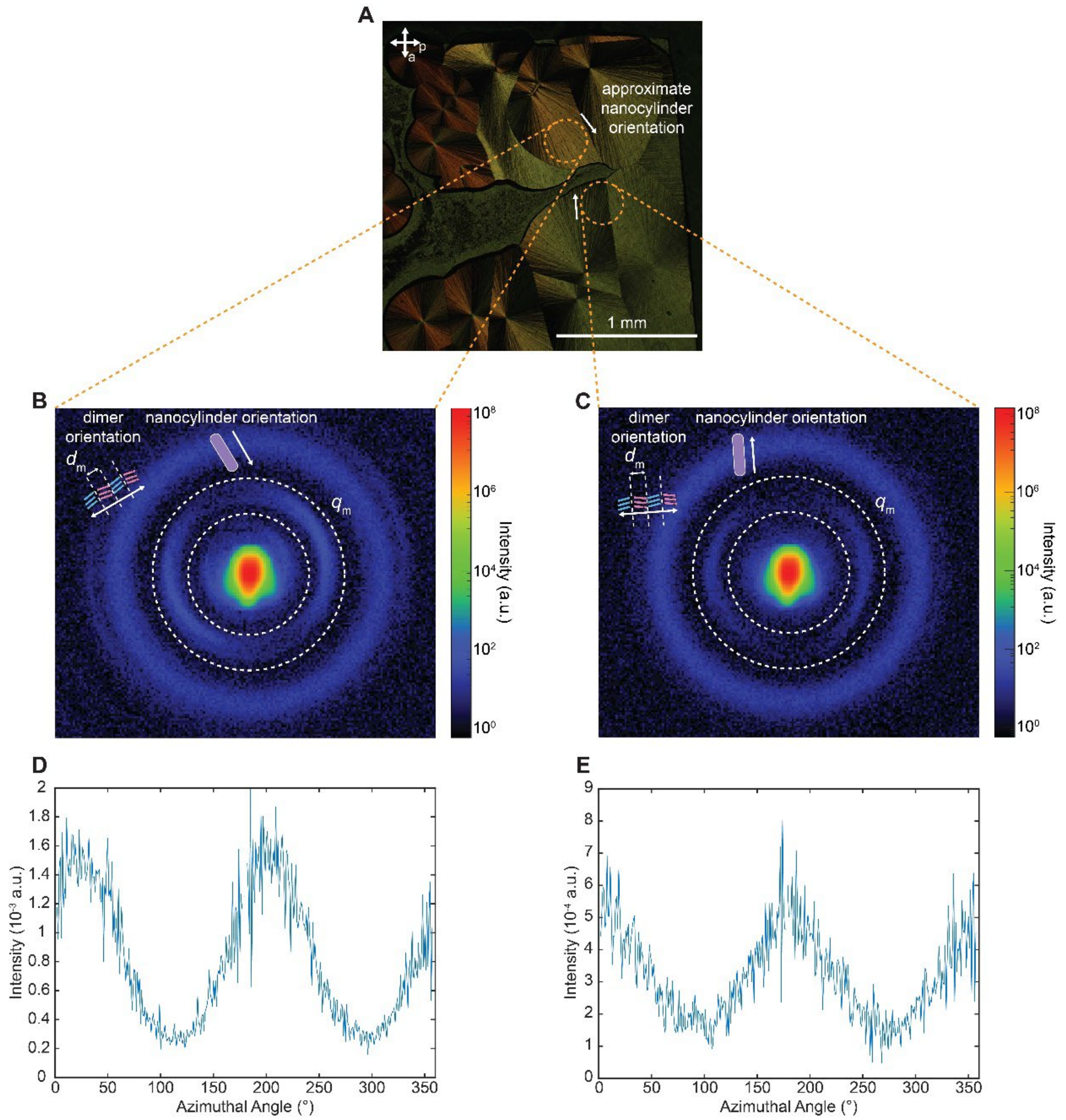

Figure S52: Determination of mesogen orientation by X-ray scattering

A) Micrograph recorded between crossed polarizers of *MP*-II isothermally crystallized at 67 °C between polyimide sheets. In *MP*-II, the nanocylinders tend to orient along the radial growth direction of the spherulite. This approximate nanocylinder orientation direction is indicated by white arrows for two regions marked by orange circles. **B-C**) 2D X-ray scattering spectra collected with the detector in the WAXS position of the **B**) top and **C**) bottom regions shown in **A** (orange circles). The peak corresponding to the length of the monomer (q_m) is marked by white circles. The 2D X-ray scattering spectra exhibit anisotropy perpendicular to the approximate nanocylinder orientation, indicating that within each nanocylinder, the dimers are aligned perpendicular to the long axis of the nanocylinder. **D-E**) Azimuthal profiles calculated for a q-value range corresponding to the q_m peak (0.20 – 0.28 Å^{-1}) from the 2D X-ray scattering spectra in **B** and **C**, respectively, showing anisotropy of the scattering intensity.

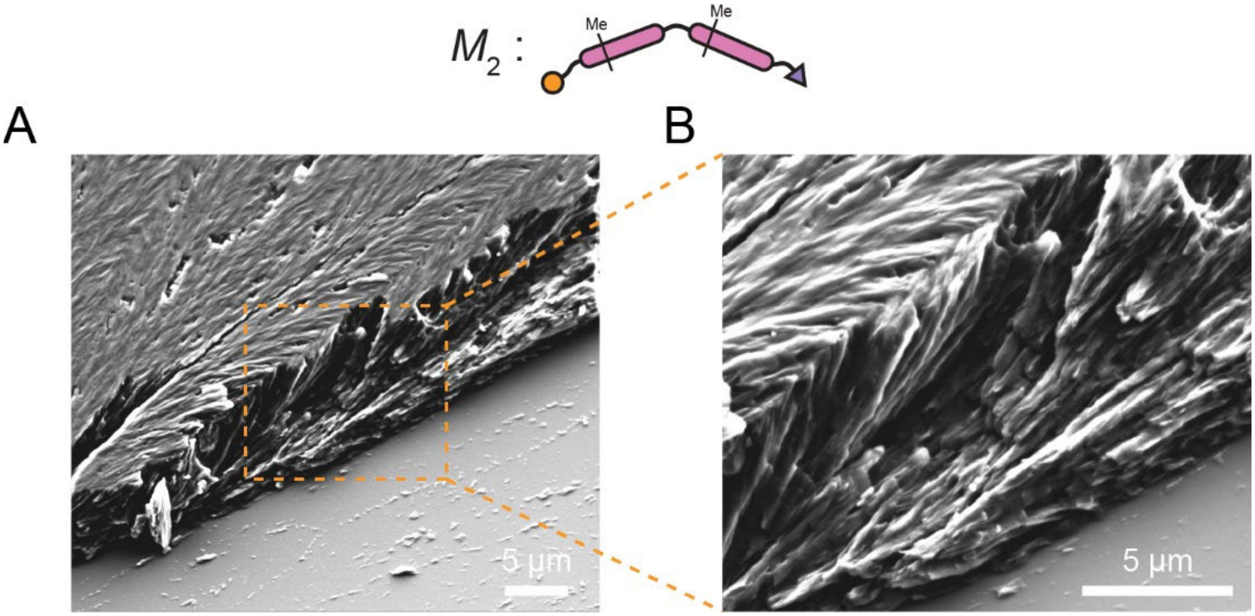

Figure S53: Scanning electron micrographs of M_2

Scanning electron micrograph showing **A**) the crystalline phase of M_2 and **B**) a magnified region of **A**. The sample was prepared by freeze fracturing of a sandwich cell after isothermal crystallization at 48 °C.

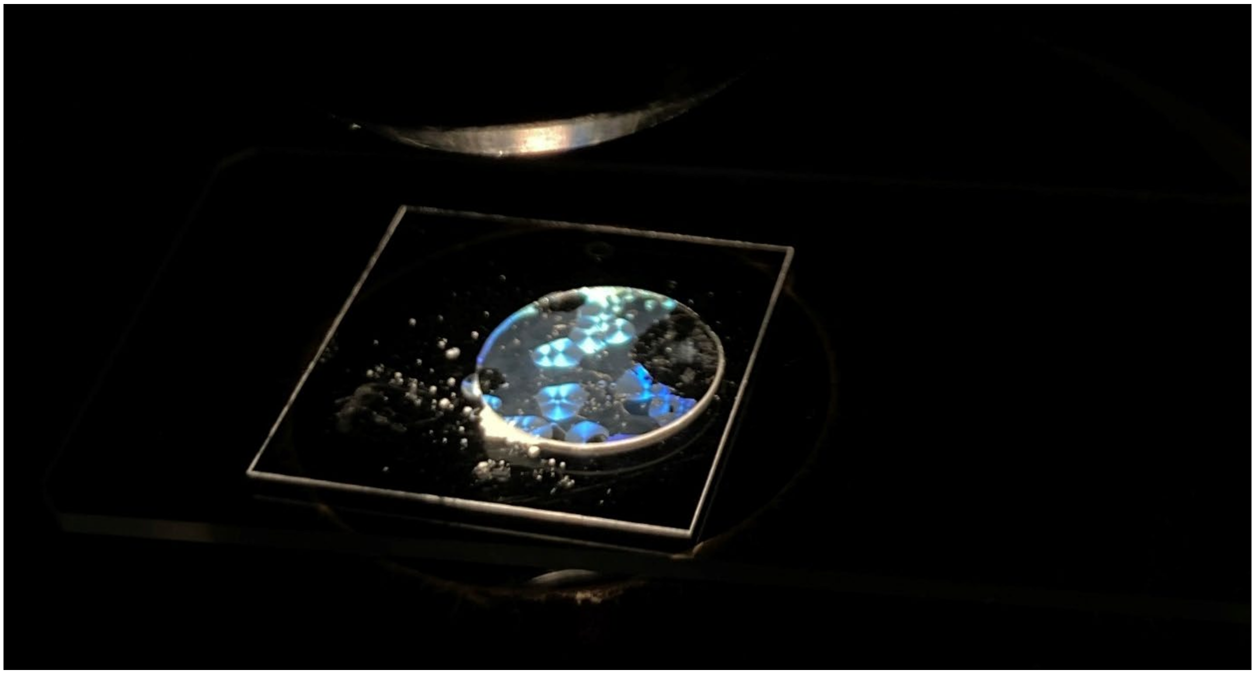

Figure S54: Scattering of blue wavelengths by *MP*-I and *MP*-II

Sample of *MP* illuminated by a microscope in transmission mode with a single linear polarizer, where *MP*-I and *MP*-II spherulites are distinguished by their scattering of blue wavelengths of light, while *MP*-III and other *MP* crystalline forms do not.

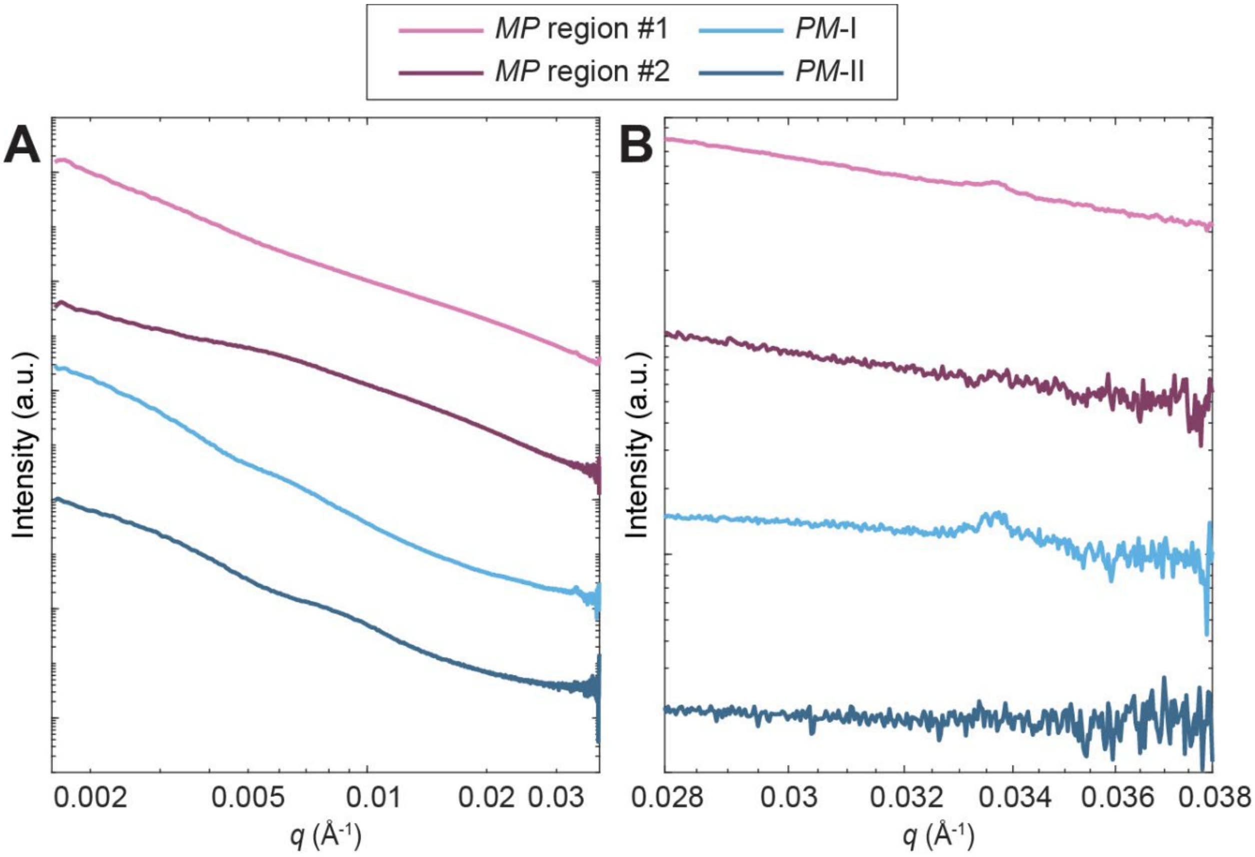

Figure S55: Ultra-small angle X-ray scattering of *MP* and *PM*

Azimuthally integrated 1D X-ray scattering patterns collected with the detector in an ultra-small angle position for bulk *MP* and *PM* samples, showing broad low-*q* peaks indicative of possible long-range order. One bulk *PM* sample was isothermally crystallized at 58 °C and is likely primarily composed of *PM*-I, while the other bulk *PM* sample was quenched to room temperature and likely consists of *PM*-II. The bulk *MP* sample was isothermally crystallized at 67 °C and measured in two locations. The measurement was taken at room temperature between polyimide substrates. The background scattering from the polyimide substrate has been subtracted. The full collected spectrum is shown in **A**, while **B** focuses on the higher-*q* region where a small peak is observed in *PM*-I and one region of *MP*, corresponding to a *d*-spacing of approximately 18 nm.

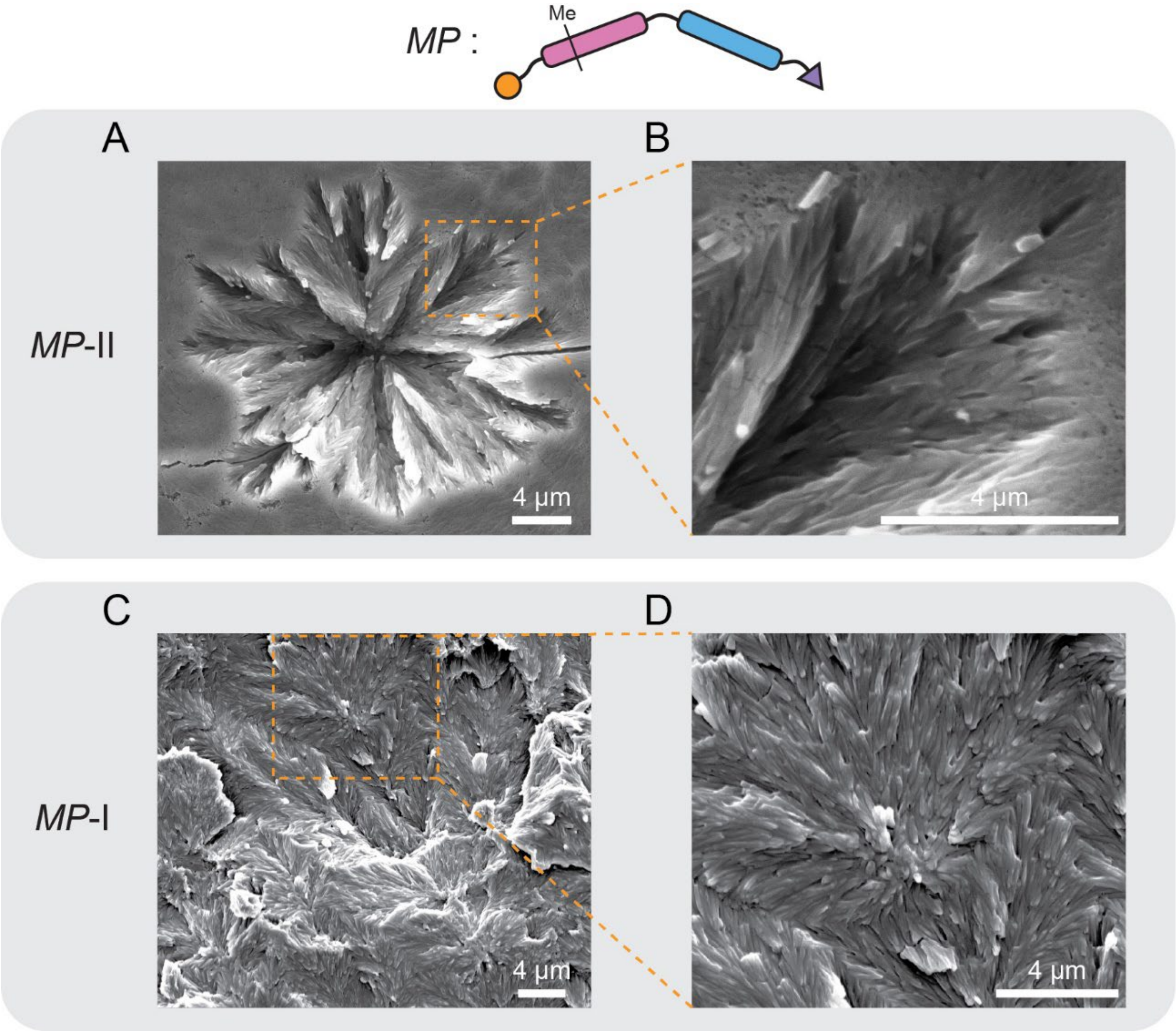

Figure S56: Scanning electron micrographs of *MP*-II and *MP*-I

Scanning electron micrograph showing **A**) a top-down view of a spherulite center of *MP*-II, **B**) a magnified region of **A**, **C**) a top-down view of *MP*-I, and **D**) a magnified region of **C**. The sample was prepared by freeze fracturing a sandwich cell between indium tin oxide coated glass slides after cooling 2 °C min^{-1} from the *Iso* melt.

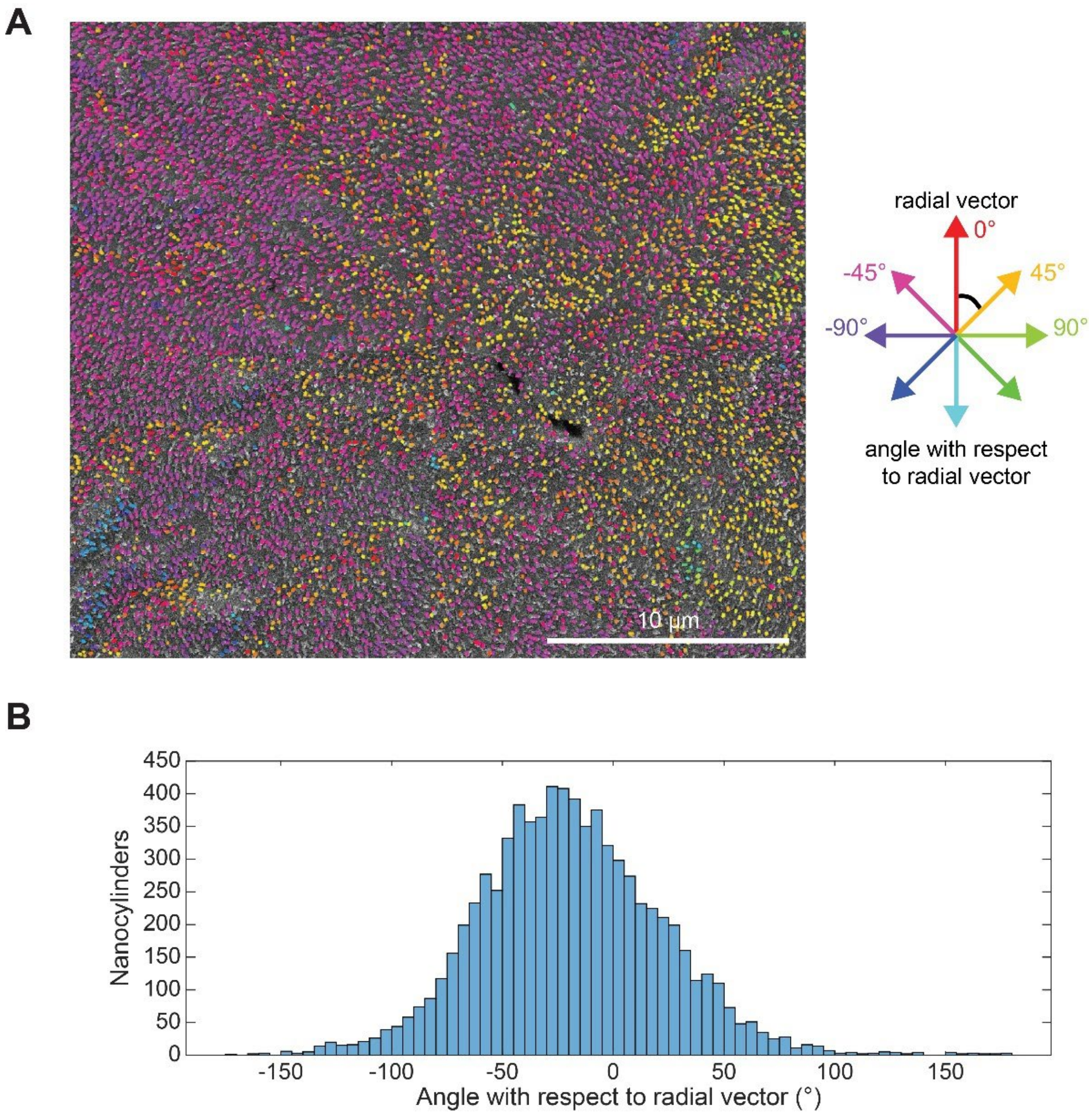

Figure S57: Cyclic sense measurement for *PM*-I spherulite center with anticlockwise orientation

A) Scanning electron micrograph (left) of the surface of a *PM*-I spherulite (the same as in **Fig. 5F**) after cooling 2 °C min^{-1} from the *Iso* melt overlaid with a heatmap in which the color represents the angle between the nanocylinder orientation and the radial vector corresponding to the nanocylinder position relative to the spherulite center (color key shown on right). The average relative orientation angle is 19° ± 41° in the anticlockwise direction. **B**) Histogram plotting the number of nanocylinders corresponding to each angle made with respect to the radial vector, where negative angles correspond to the anticlockwise direction. The average absolute value of this angle (representing the overall difference of the nanocylinder orientation from the radial vector regardless of clockwise or anticlockwise directionality) is 37° ± 27°.

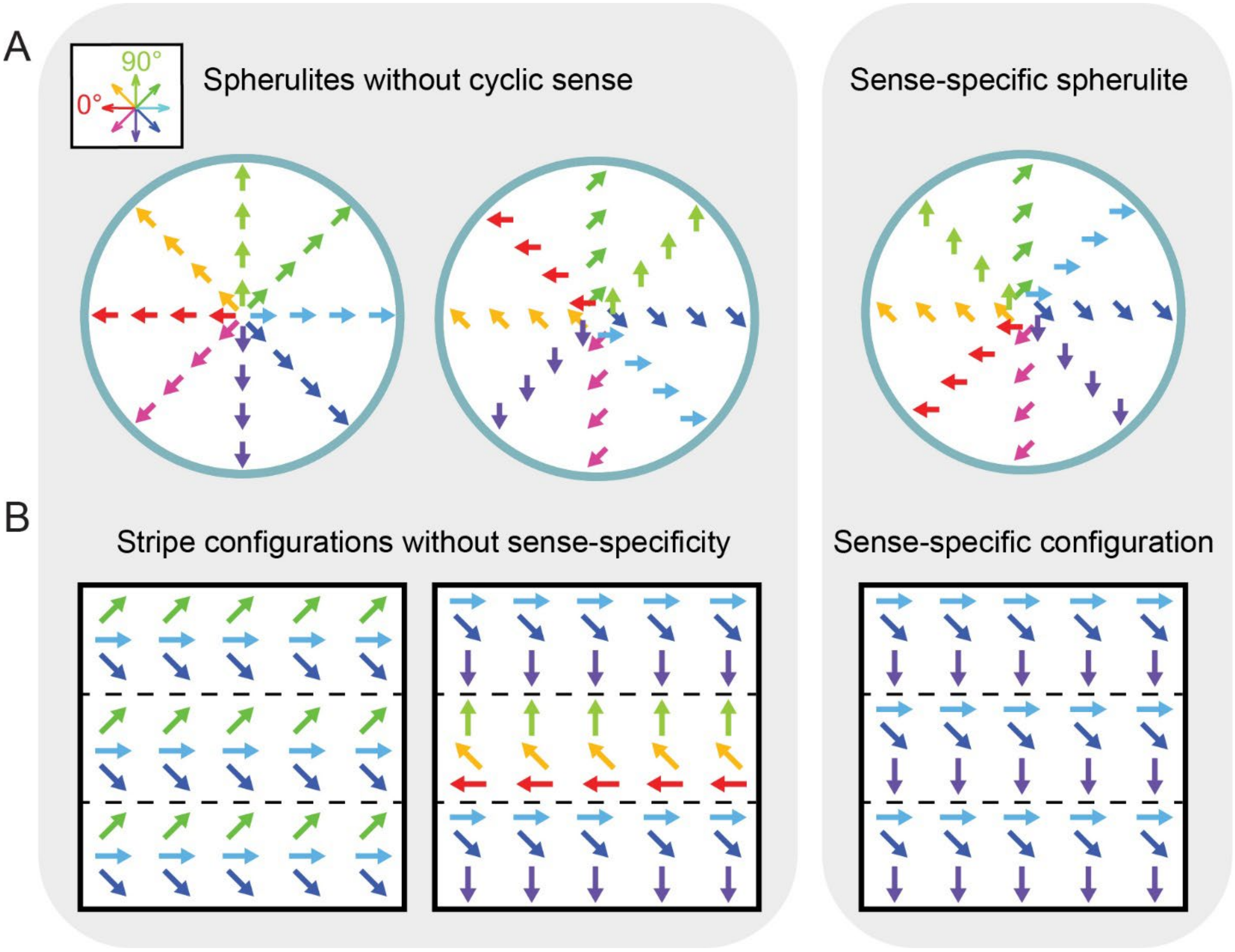

Figure S58: Sense-specific configurations of director fields

A) Schematics of director fields around a singularity without (left) and with (right) sense-specificity. In the case without a cyclic sense, the director field aligns with the radial vector or alternates orienting with a clockwise or anticlockwise angle to the radial vector. In the sense-specific case, each director is oriented at a constant angle with respect to the radial vector. **B**) Schematics of configurations of director fields in bands of domains separated by defect lines without (left, middle) and with (right) sense-specificity. In the case without sense-specificity, either each band possesses no net directionality (when considering directions perpendicular to the domain lines) (left), or each adjacent band possesses the opposite net directionality (middle). In the sense-specific case, each band possesses the same net directionality (right).

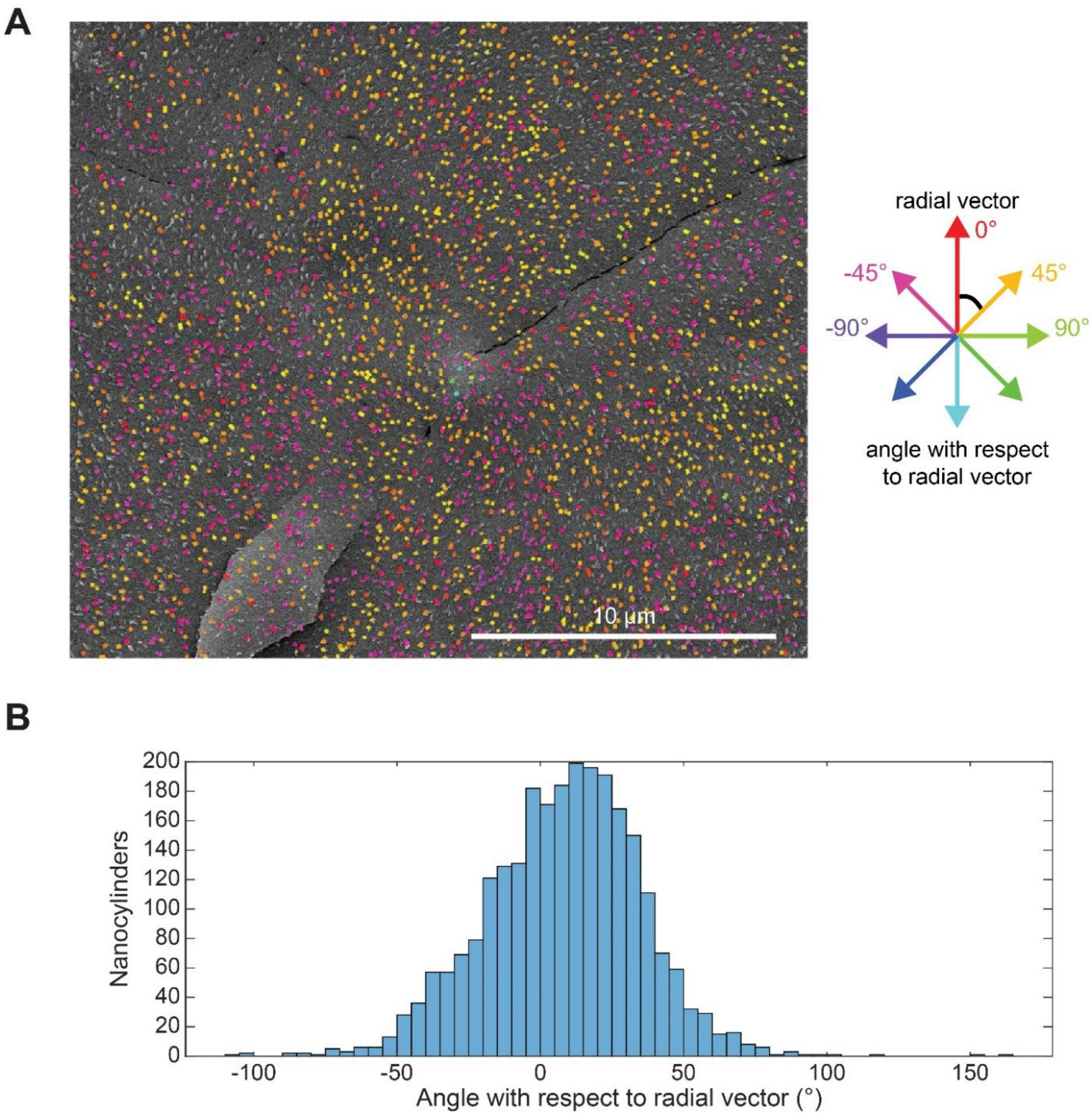

Figure S59: Cyclic sense measurement for *PM*-I spherulite center with clockwise orientation **A**) Scanning electron micrograph (left) of the surface of a *PM*-I spherulite after cooling 2 °C min^{-1} from the *Iso* melt overlaid with a heatmap in which the color represents the angle between the nanocylinder orientation and the radial vector corresponding to the nanocylinder position relative to the spherulite center (color key shown on right). The average relative orientation angle is 8° ± 27° in the clockwise direction. **B**) Histogram plotting the number of nanocylinders corresponding to each angle made with respect to the radial vector, where negative angles correspond to the anticlockwise direction. The average absolute value of this angle (representing the overall difference of the nanocylinder orientation from the radial vector regardless of clockwise or anticlockwise directionality) is 23° ± 17°.

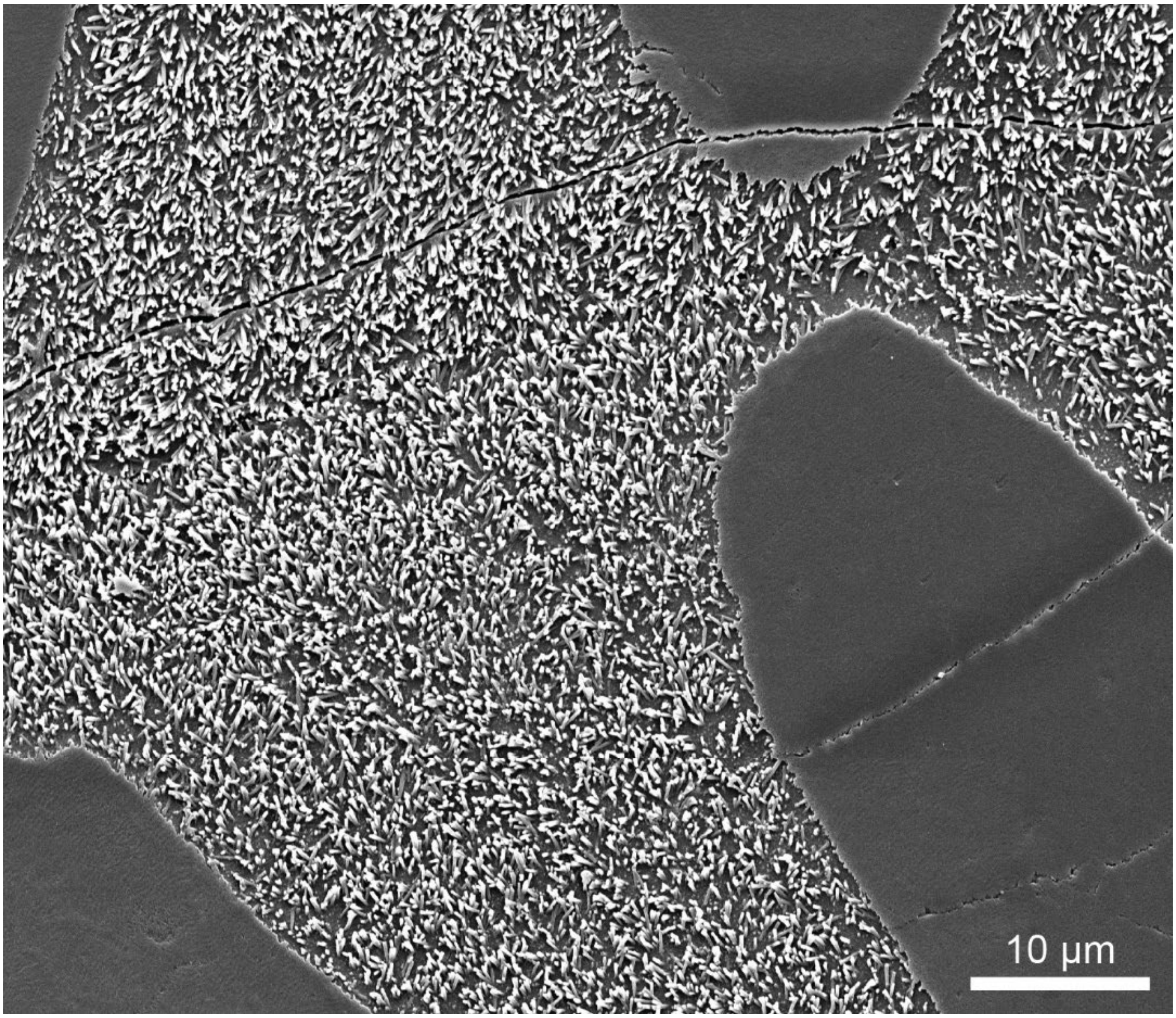

Figure S60: Dependence of nanocylinder visibility on fracture planes in *PM*-I

Top-down scanning electron micrographs of *PM*-I after isothermal crystallization at 58 °C. When the sample fractures far enough into the bulk (away from the substrate interface), nanocylinders are revealed and their orientations can be mapped.

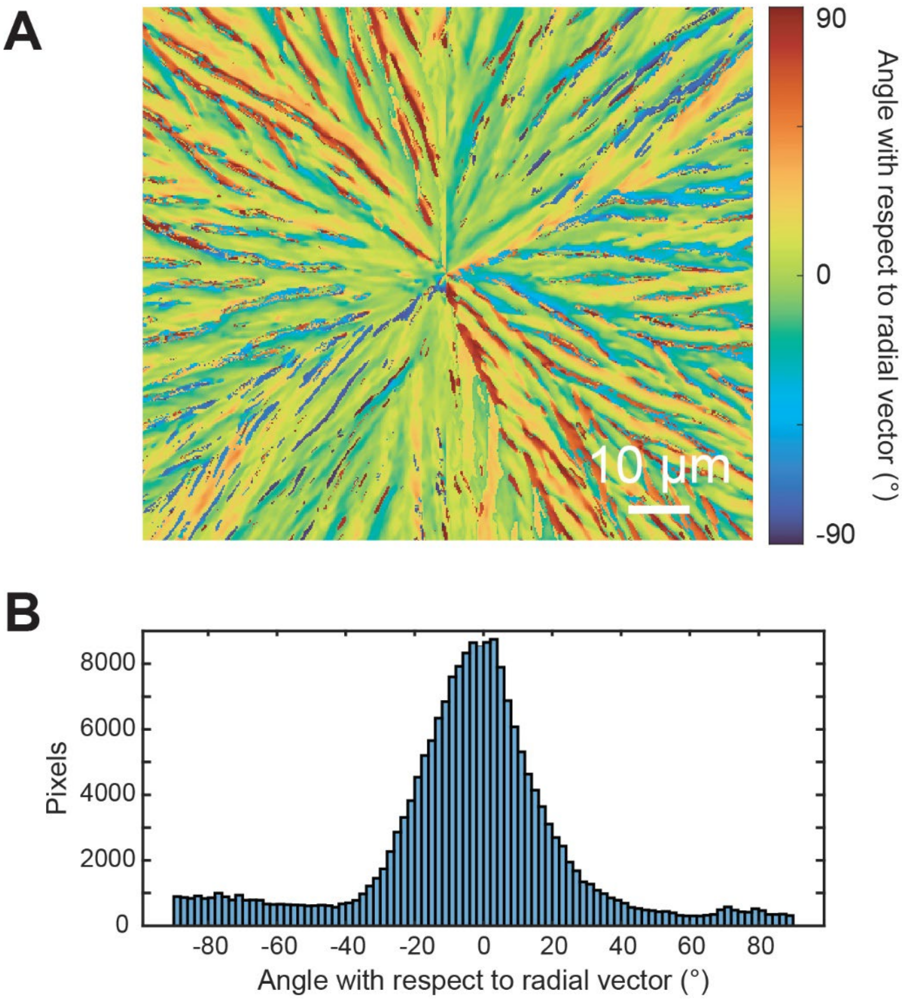

Figure S61: Cyclic sense measurement for *PM*-I using orientation of optical azimuth

A) Offset between linear retardance (LR) optical azimuth and the radial vector determined at each pixel of the micrograph of the spherulite center of *PM*-I in **Fig. 5H**. The LR azimuthal orientation is defined by plotting the larger refractive index clockwise from the horizon. The radial vector is defined as the expected orientation of the larger refractive index for a negative spherulite. The average offset angle between the LR azimuthal orientation and the radial vector is 4° ± 30° in the anticlockwise direction. **B**) Histogram plotting the number of pixels corresponding to each angle of the optical azimuth made with respect to the radial vector, where negative angles correspond to the anticlockwise direction. The average absolute value of this angle (representing the overall offset of the optical azimuth orientation from the radial vector regardless of clockwise or anticlockwise directionality) is 20° ± 22°. The sample was cooled at a rate of 2 °C min^{-1} from the *Iso* melt.

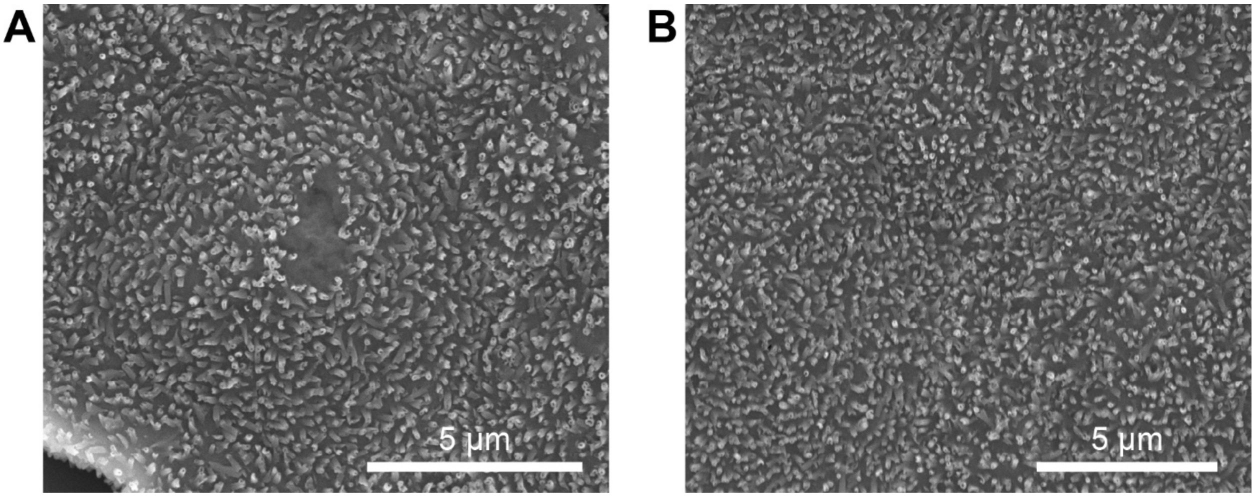

Figure S62: Nanocylinder arrangements in *PM*-II

Top-down scanning electron micrographs of *PM*-II after cooling 2 °C min^{-1} from the *Iso* melt. **A**) In some regions, circular arrangements of nanocylinders can be observed. **B**) In other regions, the nanocylinders arrange with weak long-range order.

References

(1) Chan, C. L. C.; Ostermann, E. C.; Maguire, S. M.; Schmidt, Z.; Votava, J. S.; Wąsik, P.; Webb, M. A.; Davidson, E. C. Supramolecular Bending and Twisting in the Hierarchical Self-Assembly of Monodisperse Mesogenic Oligomers. *Science Advances* **2025**, *11* (34), eadw5327. https://doi.org/10.1126/sciadv.adw5327.

(2) Stukowski, A. Visualization and Analysis of Atomistic Simulation Data with OVITO–the Open Visualization Tool. *Modelling Simul. Mater. Sci. Eng.* **2010**, *18* (1), 015012. https://doi.org/10.1088/0965-0393/18/1/015012.
